# Supplementary material for: Fingerprinting organic molecules for the inverse design of two-dimensional hybrid perovskites with target energetics
Source: Sci Adv. 2026 Apr 17;12(16):eaeb4144. doi: 10.1126/sciadv.aeb4144 (PMC13089347; doi:10.1126/sciadv.aeb4144)
Supplement: Supplementary file 1 — Supplementary Text Figs. S1 to S37 Tables S1 to S7 References [file sciadv.aeb4144_sm.pdf]

Science Advances
AAAS

Supplementary Materials for

Fingerprinting organic molecules for the inverse design of two-dimensional hybrid perovskites with target energetics

Yongxin Lyu *et al.*

Corresponding author: Tom Wu, tom-tao.wu@polyu.edu.hk; Jun Yin, jun.yin@polyu.edu.hk

Sci. Adv. **12**, eaeb4144 (2026)
DOI: 10.1126/sciadv.aeb4144

This PDF file includes:

Supplementary Text
Figs. S1 to S37
Tables S1 to S7
References

Supplementary Text

Molecular fingerprinting

All organic spacer structures in this study were stored in the Simplified Molecular Input Line Entry System (SMILES) format, a widely used textual representation of molecular structure. Molecular fingerprinting was performed to extract key structural and chemical descriptors.

For high-throughput purposes, fingerprinting was automated using the RDKit library in Python. The workflow took the SMILES representation of the organic spacer as input and returned a set of 12 organic descriptors, categorized as follows:

(1) Conjugated backbone descriptors: number of rings; percentage of ring linkages; percentage of six-membered rings

(2) Tethering ammonium descriptors: number of primary ammonium groups (-NH3+); linker length (distance between ammonium groups and backbone); and ammonium position on the backbone

(3) Heteroatom substitution descriptors: number of nitrogen atoms (pyridine-type); number of fluorine atoms, number of oxygen atoms (furan-type); number of nitrogen atoms (pyrrole-type)

(4) Side chain descriptors: number of side chain attached to linkers; number of side chains attached to the backbone

Descriptors were computed using SMARTS (SMiles ARbitrary Target Specification) patterns, enabling the identification and counting of specific functional groups. A new descriptor, the ammonium position on the backbone, was developed specifically for this work. This descriptor quantifies the relative position of tethering ammonium groups on the conjugated backbone using a distance matrix approach (see Figure S4).

High-throughput calculation

Molecular morphing. Molecular morphing was implemented as a systematic method to explore chemical space by generating variants of molecular structure. The process used reaction SMARTS patterns implemented in the RDKit library to iteratively apply predefined chemical transformations. This approach performs stepwise modifications on a starting molecular structure, generating new variants while adhering to defined chemical constraints.

The molecular morphing process began with PDMA, a well-characterized prototype molecule, represented in SMILES format. As shown in Figure S6, a set of 13 morphing operators, encoded as 17 unique SMARTS patterns, was defined to ensure that each transformation adhered to established chemical constraints. These morphing operation include: increasing number of rings, substituting heteroatoms on aromatic rings, modifying linker lengths, etc.

Each operator was applied iteratively to the starting molecule to generate new molecular structures. The newly generated molecules were stored as SMILES strings, ensuring compatibility with downstream fingerprinting and modelling workflows.

Frontier level calculation of organic spacers. The 3D molecular structures of organic spacers were generated from SMILES string using the RDKit library, which efficiently converts the molecular graph representations into 3D coordinates.

Conformational sampling was performed assuming isolated, gas-phase configurations of the spacers, independent of their incorporation into the 2D perovskite lattice.

For each spacer molecule, an ensemble of 3D conformers was generated with the Experimental-Torsion-Knowledge Distance Geometry (ETKDGv3) algorithm, which reproduces experimental torsion-angle distributions and accurately models small-ring and macrocyclic systems, followed by energy minimization using the Merck Molecular Force Field (MMFF94s).

The lowest-energy conformer from the optimized ensemble was selected for subsequent quantum-chemical calculations.

DFT calculations were then performed using Gaussian 16 package with the B3LYP functional and 6-31G** basis set to calculate the energy levels of the HOMO and LUMO.

Hybrid Perovskite Structure Generation. The hybrid perovskite structures were constructed by inserting the organic spacers into a PbI_4-based inorganic framework. Initial conformations of the organic spacers were visualized in Avogadro and adjusted according to literature-reported configurations to reflect their likely conformations within the 2D perovskites. Specific modifications include:

(1) Ensuring the linearity of linker groups to reduce aggregation effects observed in isolated forms.

(2) Constraining rotations of linked aromatic rings to reflect dihedral angles typically found in hybrid perovskites, which are smaller than those in the isolated gas phase. For instance, benzene-benzene linkages were assigned a dihedral angle of 20°; thiophene-thiophene and thiophene-benzene linkages were constrained to 0°.

A $2 \times 2 \times 1$ supercell of PbI_6 octahedra was employed (each unit cell containing four organic spacers and four PbI_4 units), starting with an ideal cubic configuration in inorganic layers. Organic spacers are aligned along the lattice c direction, with two ammonium tethering groups intercalated within the cavities formed by the PbI_6 octahedra. Organic spacers are arranged in herringbone (out-of-phase) configuration (see Figure S10). This configuration was chosen as it is frequently observed in experimental studies, with minimal influence on the electronic structure compared to other configurations. Interlayer distances between neighbouring PbI_4 layers were modulated according to spacer length, ensuring realistic structural representation. The framework was built programmatically using a combination of RDKit and pymatgen.

Perovskite Structure Relaxation. DFT-based structure relaxations were performed using the Vienna Ab initio Simulation Package (VASP) 5.4.4 with the Perdew-Burke-Ernzerhof (PBE) functional and projector augmented wave (PAW) pseudopotentials. Grimme's DFT-D3 dispersion correction with zero damping was included to account for van der Waals interactions critical to layered perovskites. Relaxation was conducted in two steps:

(1) A preliminary relaxation with a loose reciprocal density of 64 (resulting in k-point grids such as $1 \times 1 \times 1$ or $1 \times 1 \times 2$, depending on the lattice parameters along the c-axis).

(2) A tighter relaxation with a reciprocal density of 300 (resulting in k-point grids such as $3 \times 3 \times 2$, $3 \times 3 \times 3$, or $3 \times 3 \times 4$). Convergence criteria required an energy difference per atom below 5×10^{-6} eV.

Electronic Structure Calculations. To investigate the electronic properties of 2D perovskites, spin-orbit coupling (SOC) was included due to the significant relativistic effects in Pb-based compounds. The following workflow addressed the known limitations of commonly used functionals in predicting bandgap values and ensure alignment with experimental data:

1. Band structure shape identification: The band structure across the Brillouin zone was initially

calculated using the PBE+SOC functional. This functional effectively captures the qualitative shape of the band structure but is known to underestimate the bandgap for perovskite materials. The results provided a foundational map of the electronic band dispersion.

2. Accurate bandgap calculation at representative points: To obtain accurate bandgap values, the HSE06+SOC hybrid functional was applied at critical points in the Brillouin zone (Γ and Z). This method corrects the bandgap underestimation of PBE+SOC, ensuring quantitative agreement with experimental values. For structures with interlayer distances below 5 Å, calculations were performed at both Γ and Z points to capture the dispersion of inorganic bands from Γ to Z. For larger interlayer distances (greater than 5 Å), calculations were focused on the Γ point only, as the dispersion becomes negligible. A Hartree-Fock exchange (HF) percentage of 40% was used in the HSE06+SOC calculations, yielding bandgap values with a deviation of only 0.05 eV from experimental results.

   Figure S37 shows the impact of mixing factor on the organic frontier levels in DJ perovskite. A range of mixing factors (25% to 45%) has been employed in earlier studies to match experimental bandgap values. In this work, we select a mixing factor of 40%, as it yields the smallest average error of 0.05 eV. The mixing factor impacts the organic levels slightly more than the inorganic levels, though both follow very similar trends. This suggests that the energy level alignment type is unlikely to vary significantly for most proposed DJ perovskites.

   Table S1 and S2 shows the bandgap of several existing DJ perovskites with different mixing factors, confirming the mixing factor of 40% yield the smallest error compared to experimental value.

3. Band structure across the Brillouin zone: Band structure was calculated using the HSE+SOC method for the three representative candidates shown in Figure 6c–e. To reduce computational cost, only five k-points were sampled along each k-path.

High-Throughput Framework. The high-throughput DFT calculations for both organic spacers and hybrid perovskite structures were conducted on the Gadi supercomputer, utilizing a workflow based on the Materials Project (MP) input parameter template. The MP standards are widely recognized as the minimal benchmark in the field of DFT calculations, ensuring reproducibility

and consistency across studies. These parameters are particularly suitable for capturing the general structural and electronic properties of a wide range of materials.

To meet the specific demands of hybrid perovskite systems, which are characterized by strong spin-orbit coupling, van der Waals interactions, and low-symmetry structures, we further refined and tightened the computational parameters. Key refinements included:

- Energy Convergence Criterion: The MP default for electronic energy convergence (EDIFF) is 5×10^{-5} eV per calculation. For our study, we set a stricter threshold of 5×10^{-6} eV per atom to ensure reliable energy differences for low-symmetry perovskite structures.

- Plane-Wave Cutoff Energy: While the MP standard cutoff energy is typically 520 eV, we adjusted this to 480 eV, as testing showed that this value-maintained accuracy for perovskite systems while optimizing computational efficiency.

- Reciprocal Space Sampling: For initial structural relaxation, we used the default MP reciprocal density of 64 (resulting in k-point grids such as $1 \times 1 \times 1$ or $1 \times 1 \times 2$, depending on the c-axis lattice parameter). For final relaxation and electronic property calculations, we increased the reciprocal density to 300, corresponding to dense k-point grids such as $3 \times 3 \times 3$, $3 \times 3 \times 2$, or $3 \times 3 \times 4$. This stricter k-point sampling ensured accurate modelling of structural distortions and electronic properties in the layered perovskites.

- Dispersion Corrections: To account for van der Waals interactions in layered systems, Grimme's DFT-D3 dispersion correction with zero damping was included. This refinement is critical for accurately describing interlayer interactions in 2D perovskites.

The high-throughput framework was automated using the pymatgen library to generate VASP input files and parse outputs, enabling systematic and efficient exploration of $\sim$ 3,000 organic spacers and $\sim$ 400 hybrid perovskite structures. By building on the minimal standards established by the Materials Project and incorporating additional refinements specific to perovskites, we ensured that our computational results met the highest standards of accuracy and reliability for this material system.

Machine Learning

All machine learning tasks in this work were conducted using the Scikit-learn library in Python. This included data preprocessing, dimensionality reduction, training of regression and classification models, and extraction of feature coefficients for model interpretation.

Dimensional reduction for chemical space visualization. To visualize the chemical space of organic spacers, we employed unsupervised learning techniques for dimensionality reduction. We compared Principal Component Analysis (PCA), a linear dimensionality reduction method, with t-distributed stochastic neighbour embedding (t-SNE), a nonlinear technique designed to capture complex high-dimensional data structures in a lower-dimensional space. While PCA provided an initial overview, the resulting plots exhibited significant overlap between data points, limiting its ability to distinguish between structurally diverse spacers. Consequently, we selected t-SNE for its superior capability in preserving local and global relationships within the data.

Each spacer was represented numerically using a 12-dimensional fingerprint vector that encodes key structural and chemical features relevant to our analysis. The dataset, comprising $\sim 20,000$ fingerprints corresponding to $\sim 4 \times 10^6$ spacers generated across generations G_0 to G_6, was subjected to t-SNE analysis. We utilized a perplexity of 40 to balance the consideration of local and global data structures, optimizing the algorithm's sensitivity to both densely and sparsely populated regions of the chemical space.

The output of this process was a set of two-dimensional coordinates that effectively represent the high-dimensional chemical space of the spacers, enabling clear visualization of structural similarities and differences across spacer generations.

Data Collection and Input Features. The dataset comprised high-throughput computational data on 3,239 organic molecules across generations G_0 to G_4 with varying structural and electronic properties. The target properties for prediction were the highest occupied molecular orbital (HOMO) and lowest unoccupied molecular orbital (LUMO) energies of isolated organic spacers.

The input features were 12-digit organic fingerprints, which are highly relevant to the target properties due to their comprehensive representation of molecular descriptors. These fingerprints capture the chemical, electronic, and structural characteristics of the organic molecules, providing

a robust basis for predictive modelling. Correlation analysis revealed minimal redundancy among the features, negating the need for additional feature selection methods.

To ensure comparability across features, all input data were normalized to have zero mean and unit variance using the StandardScaler module in the Scikit-learn library. This normalization step mitigates bias arising from differences in feature scales, thereby optimizing model performance.

Model Training and Validation. The data was partitioned into training and test sets using an 80:20 random split, ensuring an unbiased evaluation of model performance. The training data was further subjected to five-fold cross-validation to ensure robustness and to avoid overfitting.

We evaluated the performance of several machine learning models implemented in the Scikit-learn library. Grid search with cross-validation was used to identify optimal hyperparameters for each model, as summarized in the following table.

**Method**	**HOMO**	**LUMO**
Linear regression	no hyperparameter	no hyperparameter
Lasso regression	$\alpha = 0.001$	$\alpha = 0.001$
Ridge regression	$\alpha = 1.0$	$\alpha = 5.0$
Elastic net regression	$\alpha = 0.001$	$\alpha = 0.001$
SVM (kernel = linear)	$C = 20, \epsilon = 0.1$	$C = 10, \epsilon = 0.1$
SVM (kernel = rbf)	$C = 10, \epsilon = 0.1$	$C = 10, \epsilon = 0.1$
SVM (kernel = poly)	$C = 1, \epsilon = 0.1$	$C = 1, \epsilon = 0.1$
K neighbours regressor	$n\_neighbours = 7$	$n\_neighbours = 7$
Random forest regressor	$n\_estimators = 100$	$n\_estimators = 100$

Performance metrics. The models were evaluated based on two key metrics:

(1) R^2 Score. Quantifying the proportion of variance explained by the model, with higher values indicating better fit.

(2) Root Mean Squared Error (RMSE): Providing an absolute measure of predictive accuracy, with lower values indicating smaller residual errors.

Both metrics were calculated for the training and test datasets. The results demonstrated excellent predictive performance for both linear (~ 0.95) and non-linear models (~ 0.99). This level of performance suggests that the relationships in the data are well captured by the chosen models, obviating the need for more advanced techniques, such as deep learning, for this study.

LASSO regressions were selected as the optimal model due to their decent predictive accuracy and interpretability. The importance of each input feature, both normalized coefficient and unnormalized coefficient, was analysed to verify the contribution of the organic fingerprint descriptors to the target predictions.

SHAP value analysis. To interpret the feature importance and contribution of individual organic descriptors in predicting HOMO/LUMO, we employed SHapley Additive exPlanations (SHAP) values. SHAP values provide a game-theoretic approach to quantify each feature's impact on the model's output.

Unlike the conventional approach of using the average feature value as the reference point, we calibrated all SHAP values using a baseline molecule from Generation 0. This calibration allowed us to directly compare feature contributions relative to a chemically meaningful reference, facilitating more insightful interpretations.

The SHAP values were computed and visualized using the SHAP library in Python.

Synthesis feasibility

Synthetic accessibility The presence of an organic spacer in the PubChem database was used as a proxy for synthetic accessibility. PubChem is a comprehensive chemical information repository, widely used to assess the availability and feasibility of molecular synthesis.

The neural form of organic spacers is converted to SMILES format to ensure compatibility with PubChem's search algorithms. The neutral SMILES string was used to retrieve chemical information via the pubchempy library, which interacts with the PubChem API. Key identifiers, including the compound identifier (CID) and International Union of Pure and Applied Chemistry (IUPAC) name, were extracted for each molecule.

The SA score is calculated using RDKit from organic molecules in their neutral form. These scores typically range from 1 to 10, with lower scores indicating better synthetic accessibility.

Formability topological molecular descriptors. Formability score was calculated based on five topological molecular descriptors. The distance matrix of each organic spacer is obtained using RDKit library. All hydrogen-donor nitrogen atoms was identified in the distance matrix, and their corresponding distance matrix was calculated according to the equation below.

1. Steric hindrance index (STEI): This descriptor measures the steric hindrance around a target nitrogen atom, calculated as the inverse sum of the cubed distances to all other atoms in the molecule:
$$\mathrm{STEI}_{N_i} = \sum_{j=1}^{n} \frac{1}{\left(d_{N_i-\mathrm{Atom}_j}\right)^3} \tag{S1}$$
A larger STEI indicates a higher density of nearby atoms, increasing steric hindrance and potentially reducing the likelihood of forming hydrogen bonds with the inorganic framework.

2. Eccentricity: Eccentricity quantifies the molecular shape with respect to a nitrogen atom, measuring the longest distance between the nitrogen and any other atom in the molecule:
$$\mathrm{Eccentricity}_{N_i} = \max\left(d_{N_i-\mathrm{Atom}_j}\right) \tag{S2}$$
Larger eccentricity values correspond to more elongated molecules, which are favorable for forming 2D perovskite layers.

3. Number of rotatable bonds (NumRot): This descriptor reflects the flexibility of the ammonium group tethered to the nitrogen atom, calculated as the minimum distance from the nitrogen to the nearest atom in the conjugated backbone:
$$\mathrm{Num\_Rot}_{N_i} = \min\left(d_{N_i-\mathrm{RingAtom}_j}\right) \tag{S3}$$
A higher number of rotatable bonds improves flexibility, facilitating the anchoring of the organic spacer to the inorganic framework.

4. N-N pair distance (Dis_{NN}): This descriptor measures the spatial separation between two nitrogen atoms in a molecule:
$$\mathrm{Dis}_{N_i-N_j} = \frac{1}{\left(d_{N_i-N_j}\right)^2} \tag{S4}$$

A smaller value indicates a larger distance, reducing repulsive interactions and enhancing structural stability in 2D perovskite layers.

5. Ammonium position in backbone: this descriptor is one of the 12 digit in fingerprinting. See Figure S4 for more details.

$$Ammonium\_position_{N_i-N_j} = \frac{(d_{Ni-Nj}) - d_{\text{primary ammonium}}}{\text{Max}(d_{\pi\text{Atom } i-\pi\text{Atom } j})} \tag{S5}$$

To translate raw descriptor values into a unified formability metric, each was mapped onto a probablistic scale using a sigmoid transformation. The sigmoid parameters were empirically fit based on reported organic spacers and their formed dimensions, i.e., 2D perovskite-forming (positive) and non-2D perovskite forming (negative) organic spacers. To ensure smooth gradation in score values and avoid overfitting to binary labels, a smearning factor was introduced into the sigmoid function. The overall formability score was calculated as the average of the transformed values, providing a continous score that reflects the likelihood of successful 2D perovskite formation.

Formability score calculation. The formability score is computed by averaging five logistic regression outputs, each based on a molecular descriptor x_i, with a smearing factor σ introduced to control the sharpness of the sigmoid transition:

$$\text{score}_i = \frac{1}{1 + \exp\left(-\left(\sigma \cdot a_i \cdot x_i + b_i\right)\right)}$$

where a_i and b_i are the fitted parameters, and $\sigma = 0.15$ is the smearing factor (shared across all descriptors). The final formability score is:

$$\text{Formability Score} = \frac{1}{5} \sum_{i=1}^{5} \text{score}_i$$

With descriptor-specific parameters:

$$
\begin{aligned}
\text{STEI:} &\quad a = -156.259, \quad b = -2.060 \\
\text{Eccentricity:} &\quad a = 117.730, \quad b = 1.112 \\
\text{Linker position:} &\quad a = 54.558, \quad b = 1.643 \\
\text{Number of rotatable bonds in tail:} &\quad a = 43.249, \quad b = 1.529 \\
\text{Nitrogen-nitrogen distance (disNN):} &\quad a = -22.249, \quad b = -2.097
\end{aligned}
$$

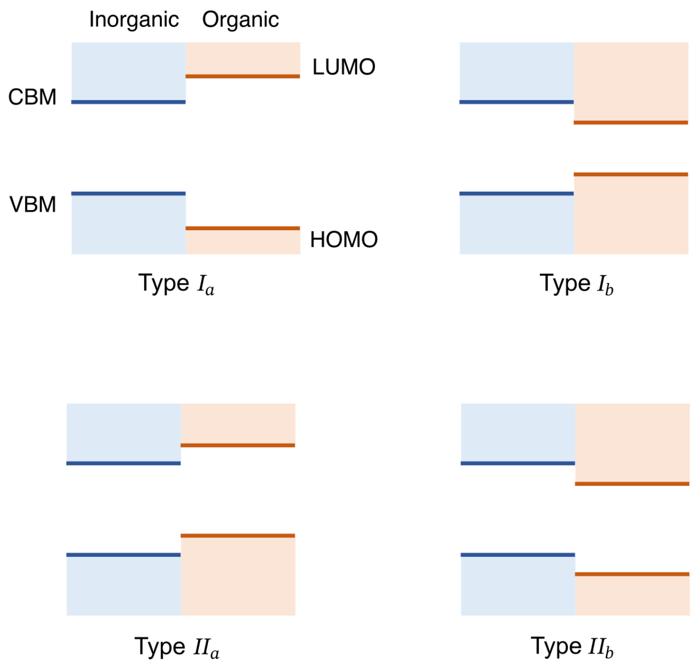

Figure S1: Schematic representation of energy level alignment types in 2D perovskites. In type I alignment, both low-energy electrons and holes are localized in the same component: type Ia in the inorganic layers and type Ib in the organic layers. In type II alignments, electrons and holes are separated between different components: in type IIa, electrons are localized in the inorganic layers and holes in the organic layers, whereas in type IIb, the reverse occurs. It should be noted that the designations “a” and “b” are sometimes interchanged in the literature depending on the component being emphasized (*31*). In other studies, only type I and type II are referenced without further categorization (*33*), and type Ib in our context is occasionally described as “reversed type I” (*34*). Notably, alignment type Ia is referred to as type Ib in some literature (*31,72*); here, we defined type Ia as the configuration where inorganic states serving as the band edges.

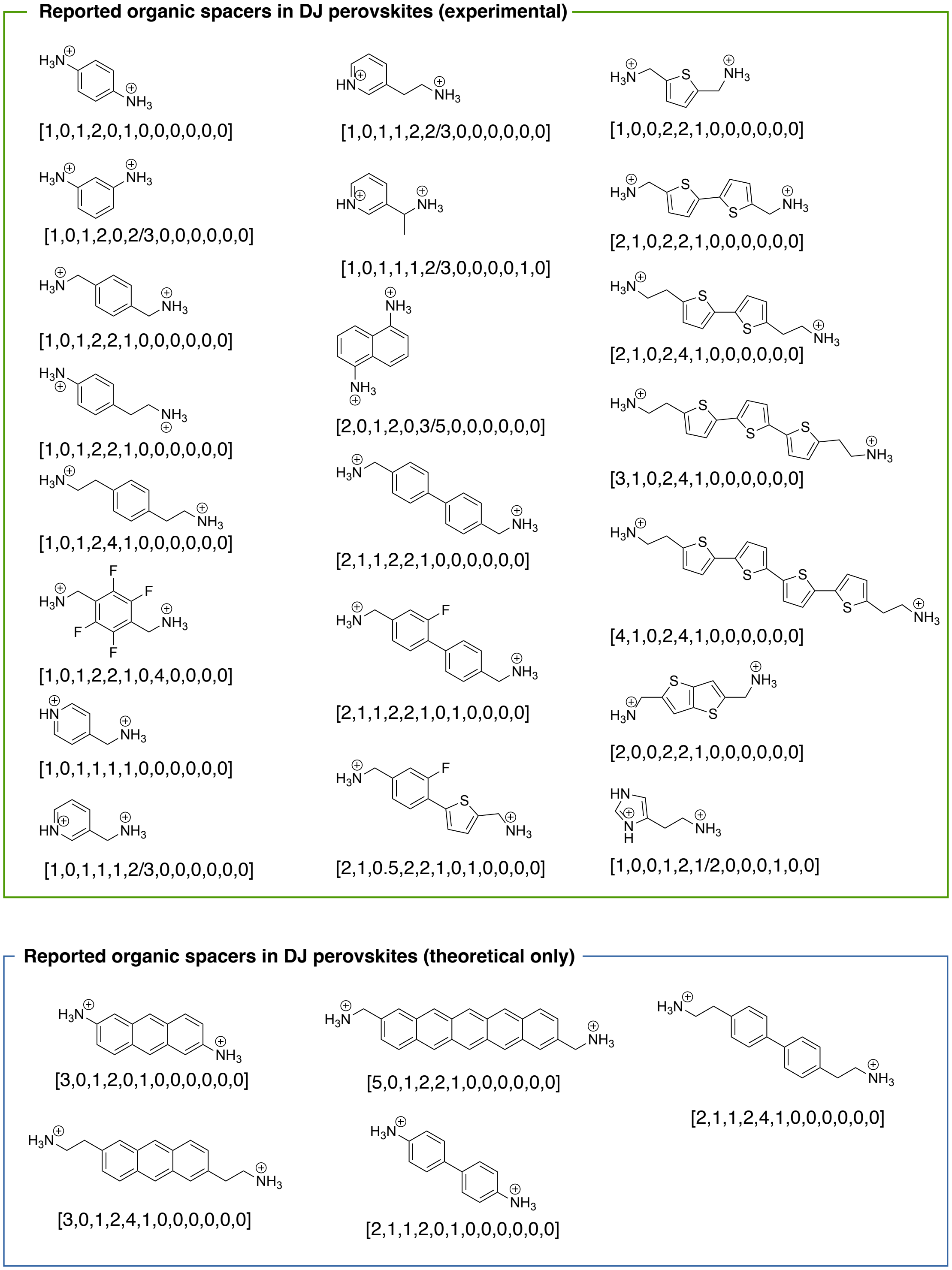

Figure S2: Reported organic spacers included in this study with their molecular fingerprint.

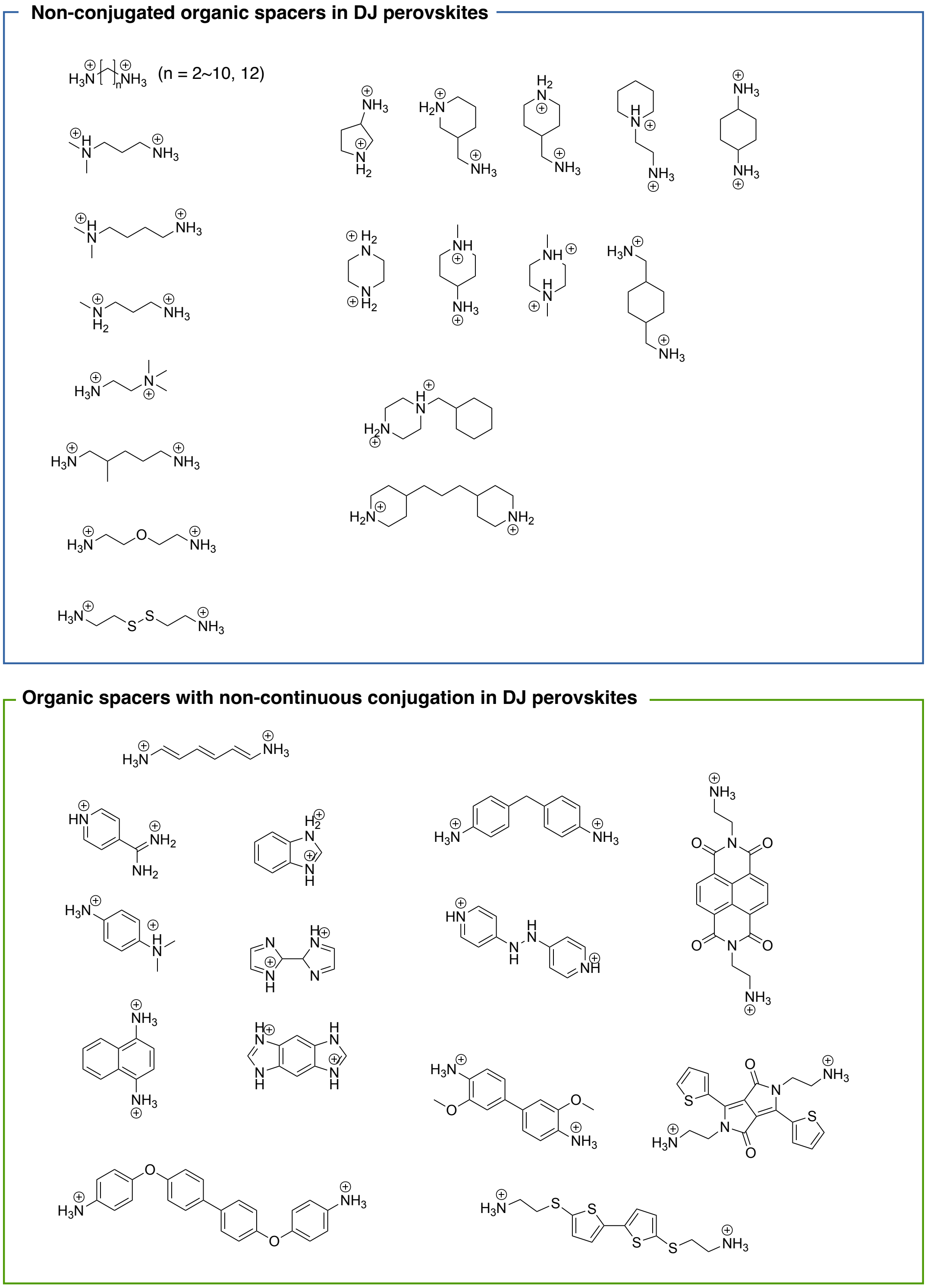

Figure S3: Organic spacers excluded from the scope of this study, for example ones with alkyl backbones or non-continuous conjugation.

Ammonium position descriptor

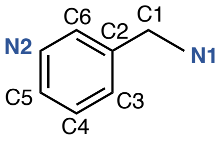

	N1	C1	C2	C3	C4	C5	C6	N2
N1	0	1	2	3	4	5	3	4
C1	1	0	1	2	3	4	2	3
C2	2	1	0	1	2	3	1	2
C3	3	2	1	0	1	2	2	3
C4	4	3	2	1	0	1	3	2
C5	5	4	3	2	1	0	2	1
C6	3	2	1	2	3	2	0	1
N2	4	3	2	3	2	1	1	0

Distance matrix

$$Descriptor \ = \frac{(d_{N1-N2}) - d_{primary \ \ ammonium}}{Max(d_{\pi Atom \ i - \pi Atom \ j})}$$

0 < Descriptor <= 1 (para position)

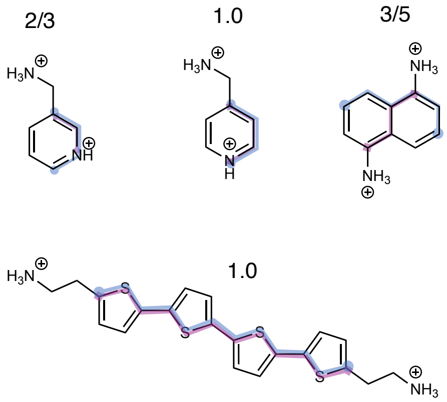

Figure S4: Illustration of the ammonium position descriptor. The ammonium position descriptor is derived from the molecular skeleton using a distance matrix. This descriptor is calculated as the ratio of the maximum distance along the conjugated backbone to the distance between the tethering ammonium group and the backbone. Representative organic spacers with their corresponding ammonium position descriptors are shown.

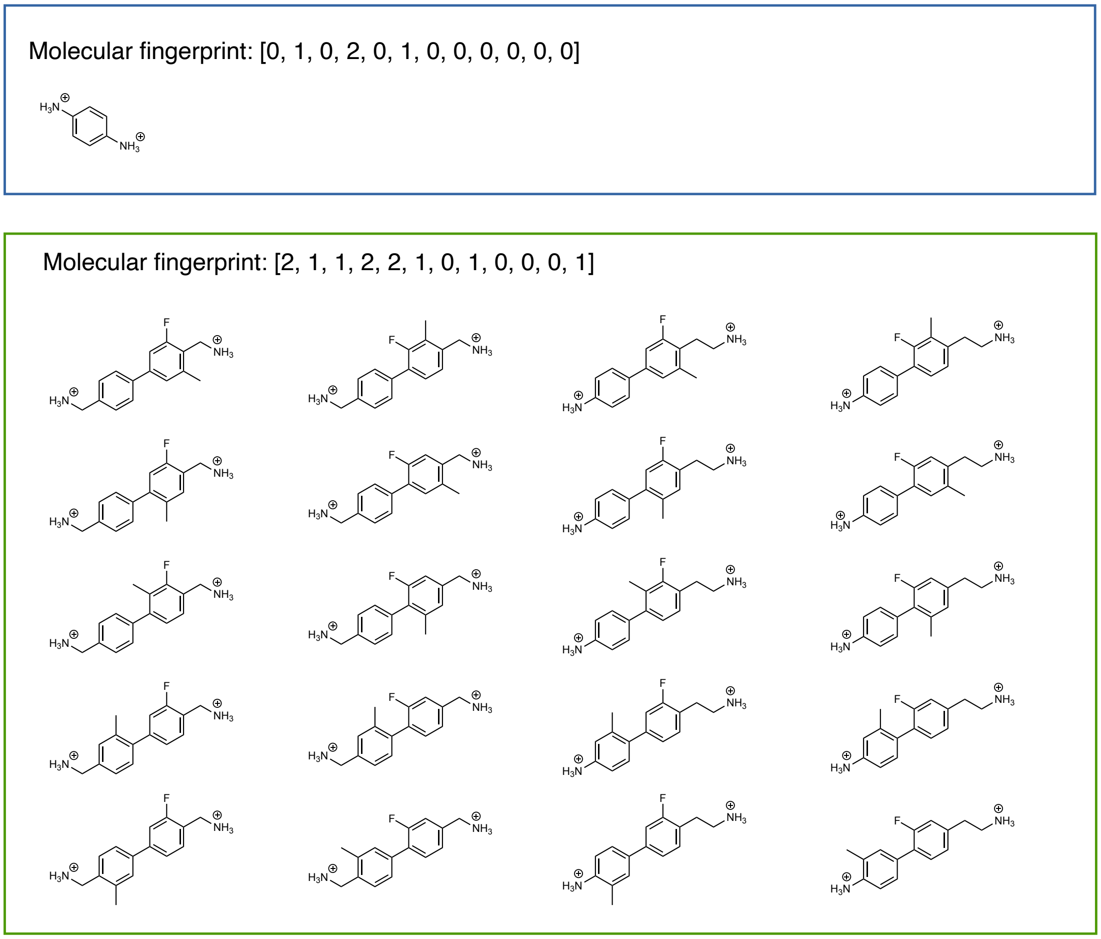

Figure S5: An illustration of one-to-one (top) and one-to-multiple (bottom) mappings between a molecular fingerprint and its corresponding organic spacer(s). The upper panel shows a one-to-one mapping where a fingerprint corresponds to a distinct organic spacer. The lower panel shows another example where multiple organic spacers (isomers) sharing the same fingerprint, including the molecule depicted in Fig. 2. These isomers may differ in features such as the position of heteroatom substitution, side chain placement, or the linker length between tethering ammonium groups. The variations may affect to a certain extent the chemical and physical properties of the cations, but the energy levels are almost the same. On the other hand, these molecules may have different levels of synthesis feasibility, which warrants elucidation based on future detailed analysis and experimental efforts.

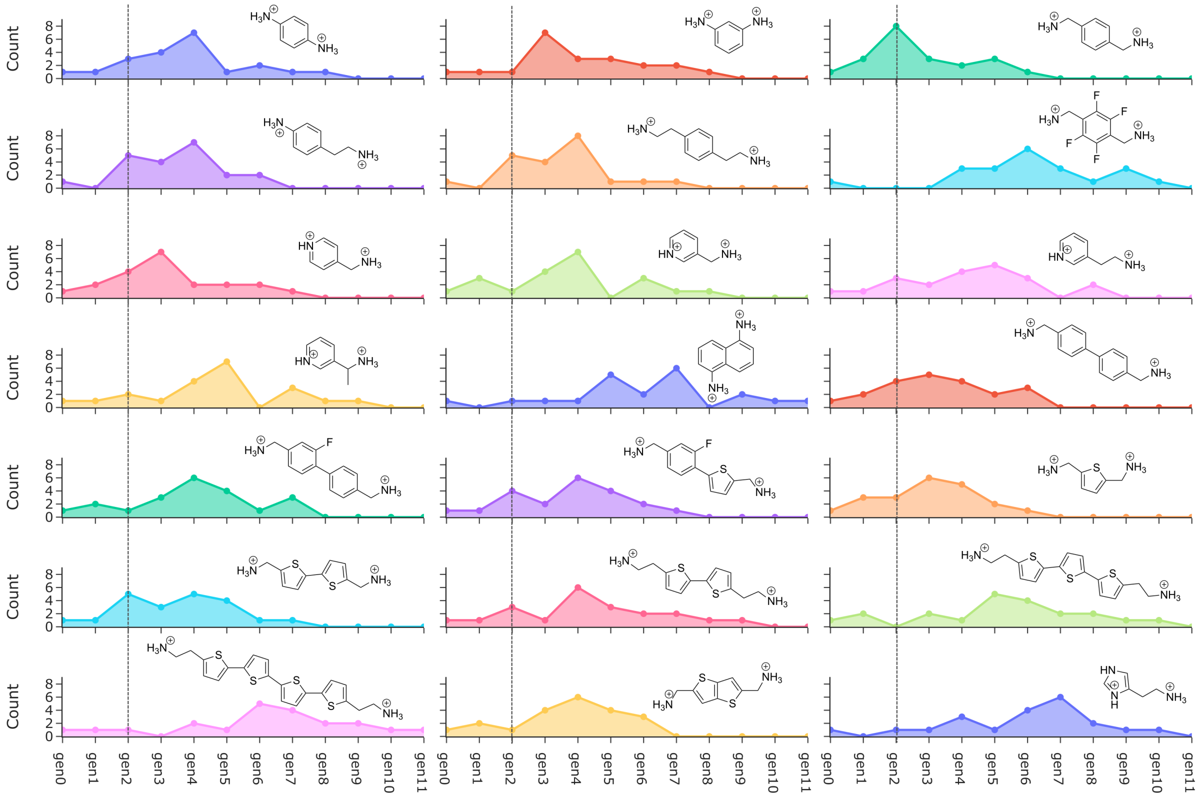

Figure S6: Rationale for selection of PDMA as the generation G_0 organic spacer. The distribution of existing spacers across generations when different candidates are selected as G_0 molecules is depicted. PDMA (top right) was selected because it results in most existing spacers appearing in early generations, demonstrating its structural simplicity and suitability for easy transformation into other molecular structures via morphing operations.

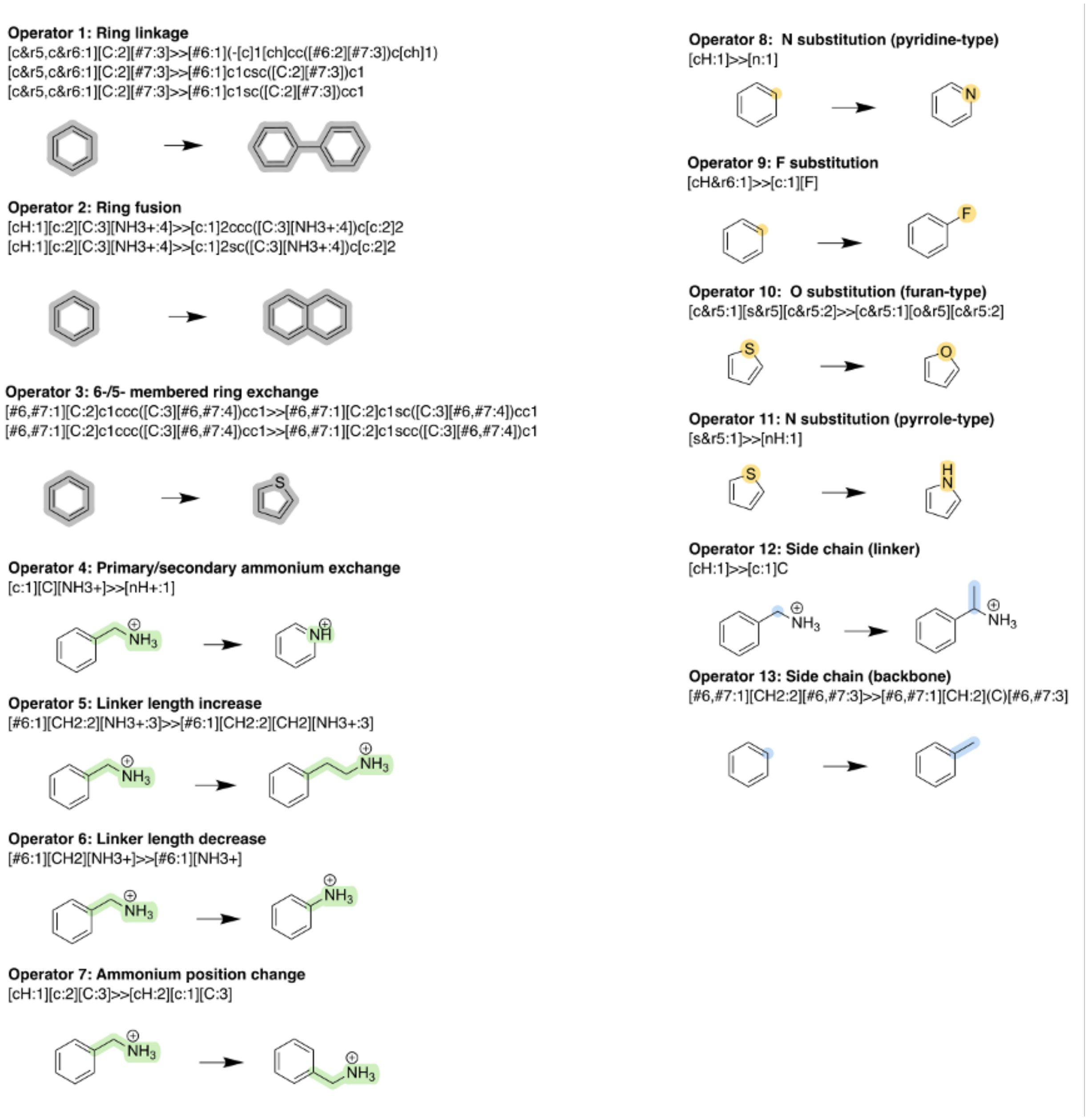

Figure S7: List of molecular morphing operators used in this study for generation of hypothetical organic spacers. A set of 13 morphing operators, encoded as 17 unique SMARTS patterns, was defined to ensure that each transformation adhered to established chemical constraints.

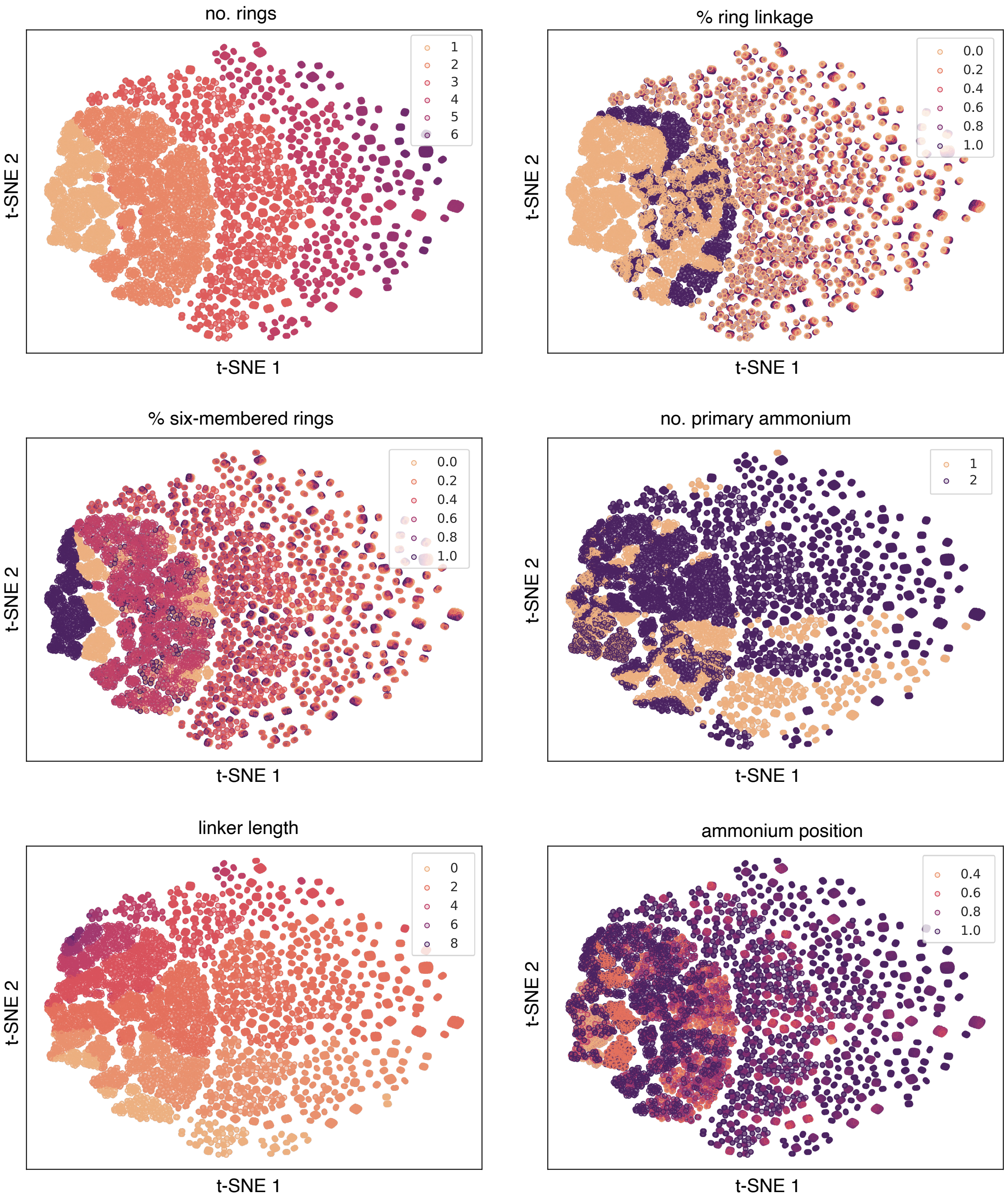

Figure S8: Visualization of the chemical space with respect to organic descriptors in molecular fingerprint (Part 1).

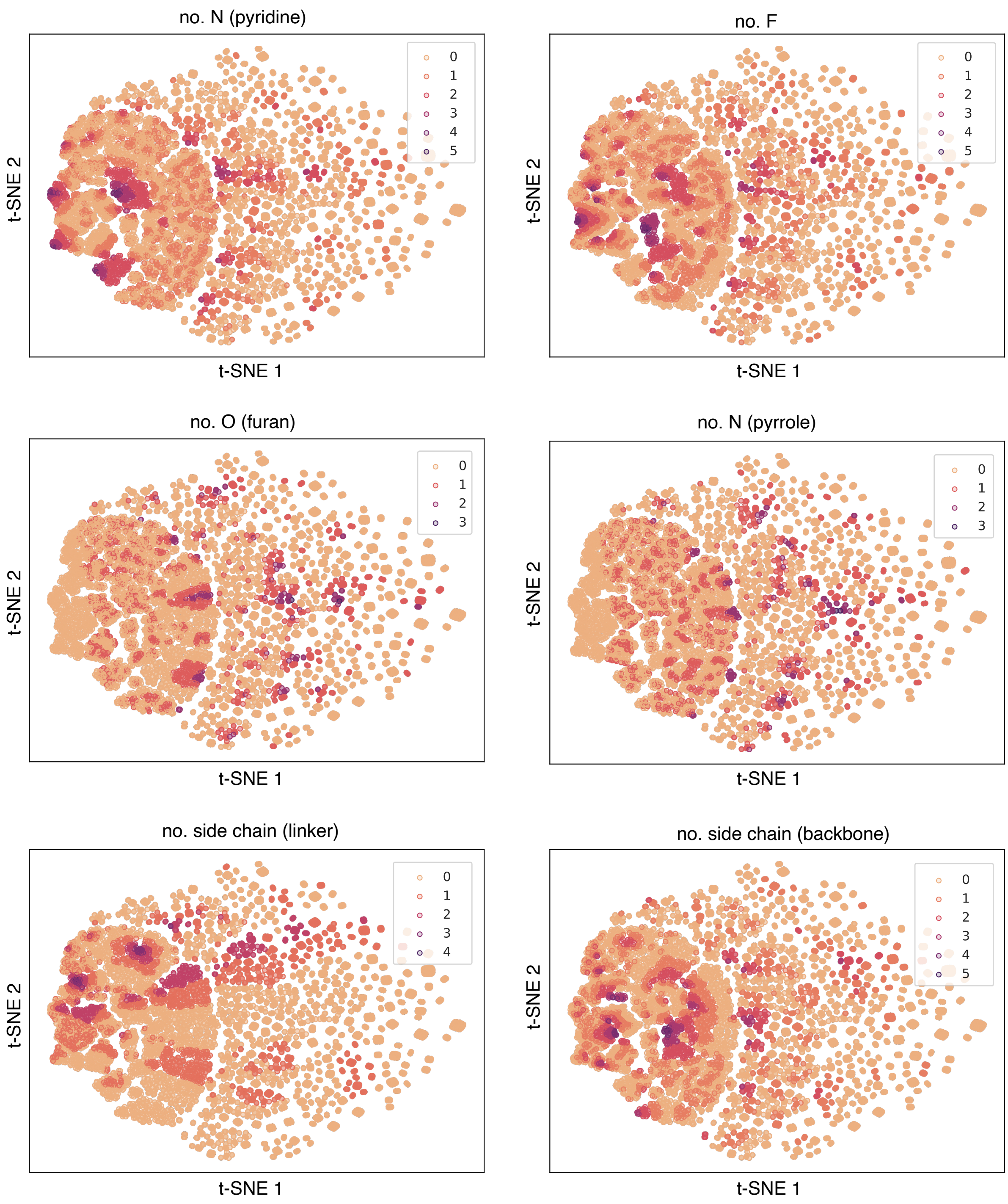

Figure S8: Visualization of the chemical space with respect to organic descriptors in molecular fingerprint (Part 2, continued).

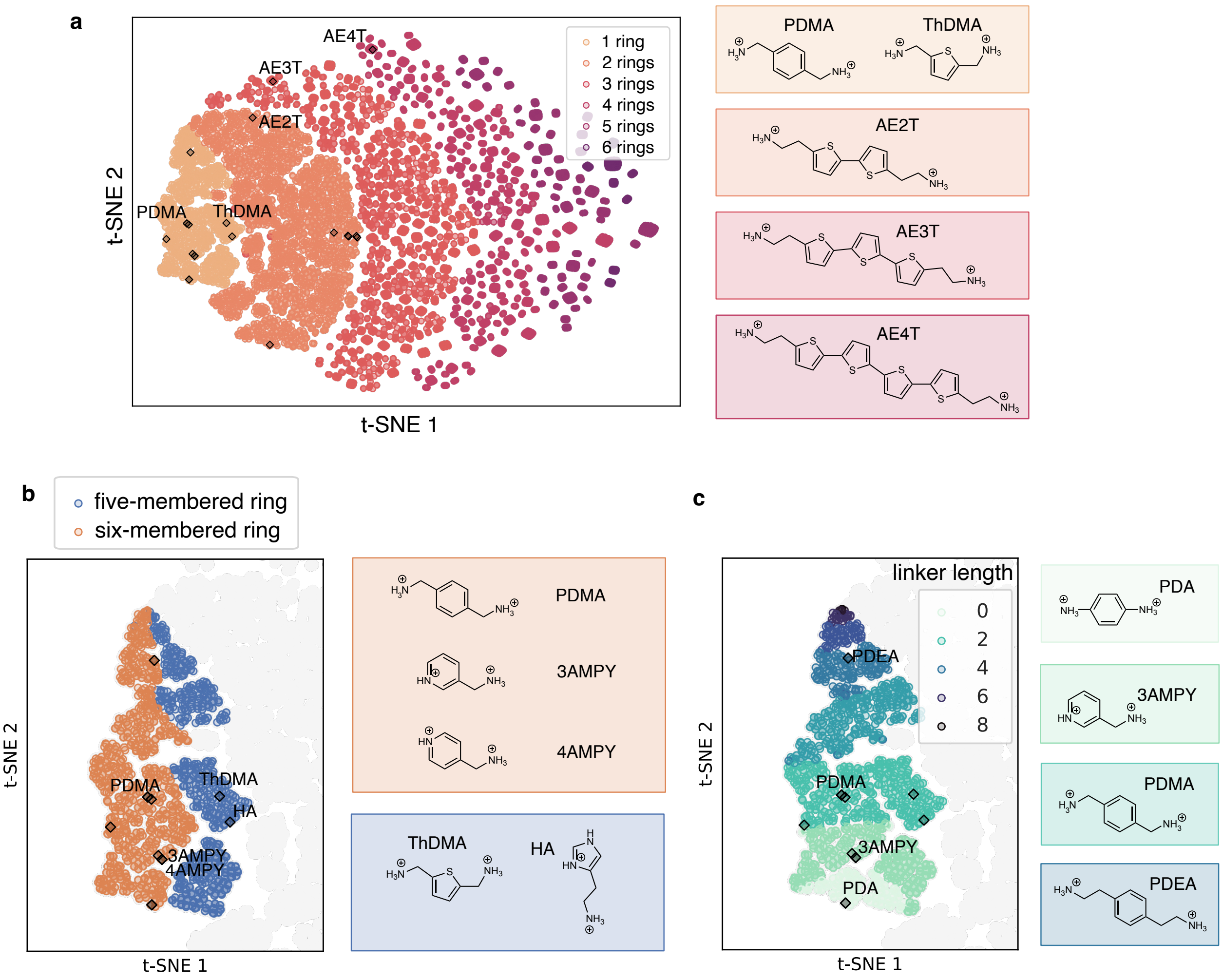

Figure S9: Chemical space visualization of existing spacers. **a** Complete chemical space. **b,c** Enlarged views highlighting spacers containing a single aromatic ring.

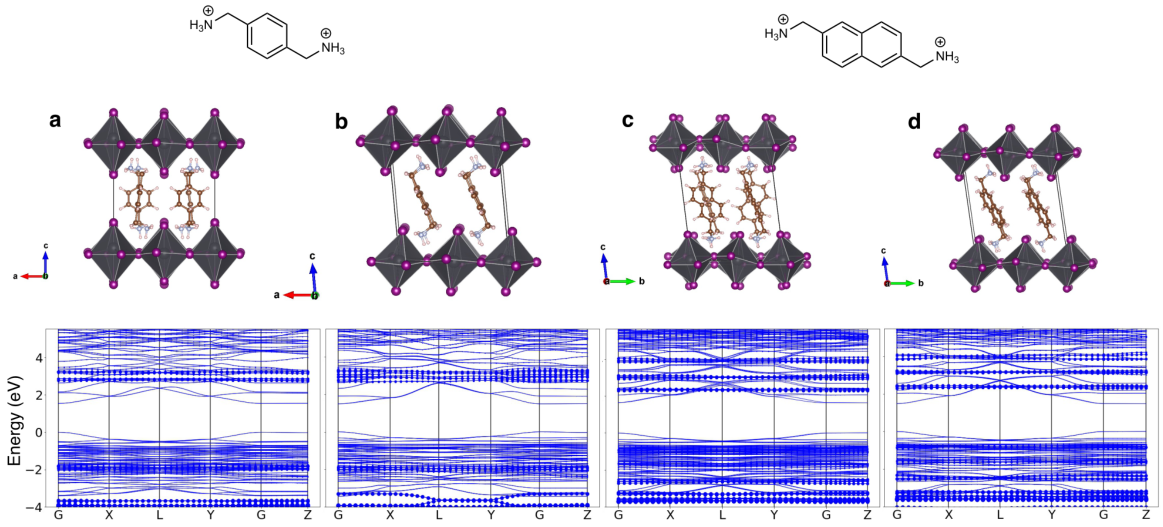

Figure S10: Crystal and band structure of DJ perovskites with different organic spacer packing arrangements. **a** and **c** show the out-of-phase (herringbone) arrangement of organic spacers. This configuration is commonly observed in reported 2D perovskites, and our DFT calculation indicate this type of packing leads to minimal dispersion of molecular orbitals. **b** and **d** show the in-phase arrangement, a configuration typically found in oligomer thiophene-based spacers. Our DFT calculations suggest that this configuration can alter the energy dispersion of certain molecular orbitals, due to the electronic coupling among tightly packed organic spacers (*32*). For the two spacers shown here, no significant change in the organic frontier orbital was observed, although one occupied orbital in **b** shows a enhanced dispersion of $\sim$ 0.7 eV. While one study reported that the out-of-phase configurations have a lower total energy (*50*), there is no consensus on the preferred packing, and the exact packing type should be confirmed by both DFT calculations and by experimental measurements whenever a new organic ligand is explored to synthesize 2D hybrid perovskites. In the current work, for consistency, we adopt the out-of-phase packing pattern across all DJ perovskites. We anticipate that molecular packing will have only a minor influence on the predicted energy-level-alignment types for most candidates. However, as suggested by previous experimental work(*57*), when the linker lengths are long and face to face π-π stacking present, additional factors such as in-plane orbital dispersion, organic–inorganic hybridization, and subtle shifts in the inorganic band edges may arise. These effects could potentially alter the energy-level alignment in certain cases and therefore warrant further investigation in future studies.

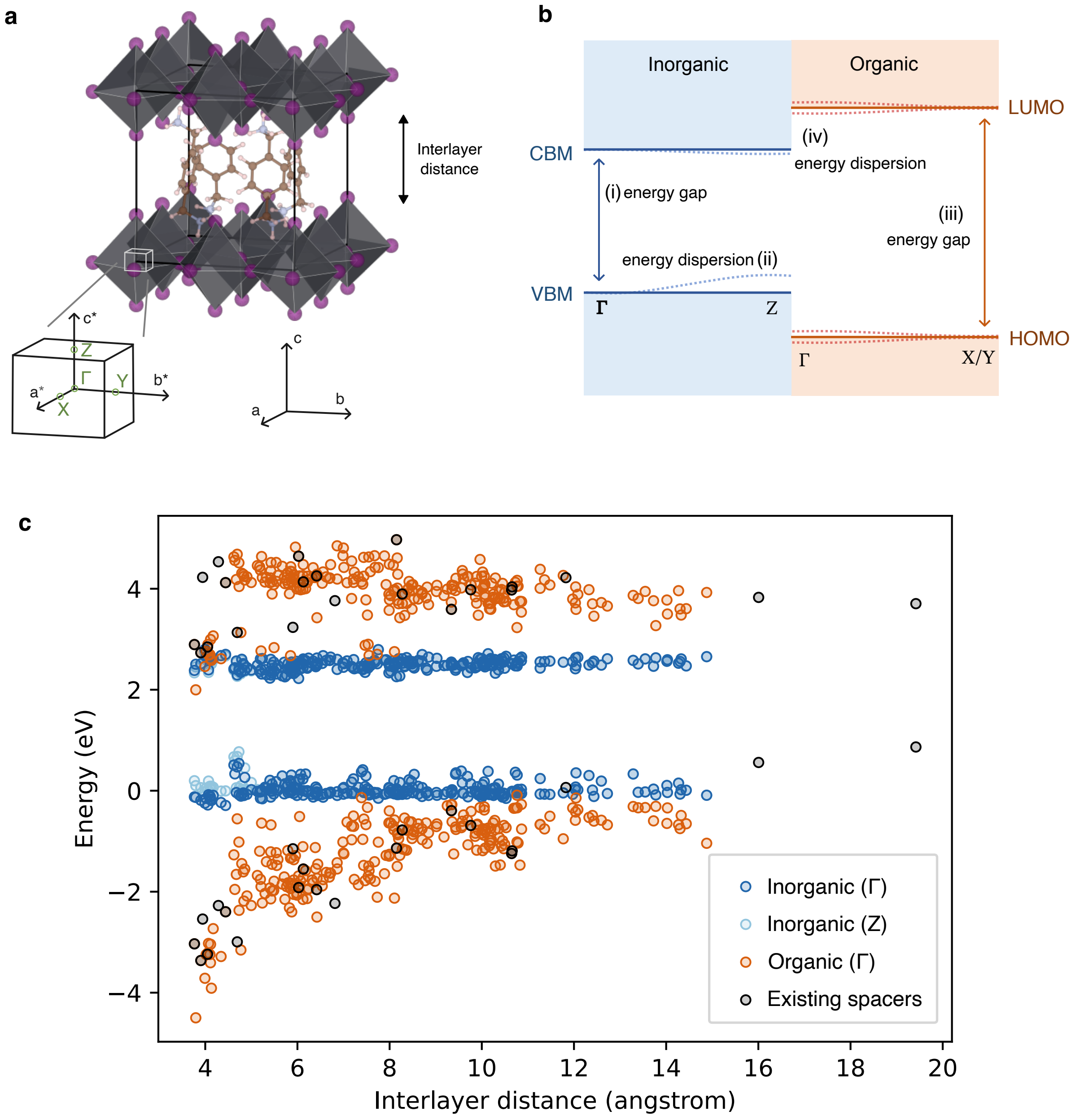

Figure S11: General consideration of energy level alignment in DJ perovskites. **a** model crystal structure of DJ perovskite constructed by inserting organic spacers between inorganic layers. **b** Four factors affecting the energy level alignment in DJ perovskites.: (i) energy gap at the inorganic band edge (Γ point), (ii) inorganic band dispersion (Γ to Z), (iii) energy gap between organic frontier orbitals, and (iv) frontier orbital dispersion. **c** Quantitative analysis of energy level alignment in 252 DJ-phase perovskites in $G_0 - G_2$ and existing spacers (experimentally reported), illustrating significant shifts in organic frontier levels (several eV) compared to inorganic levels (<1 eV).

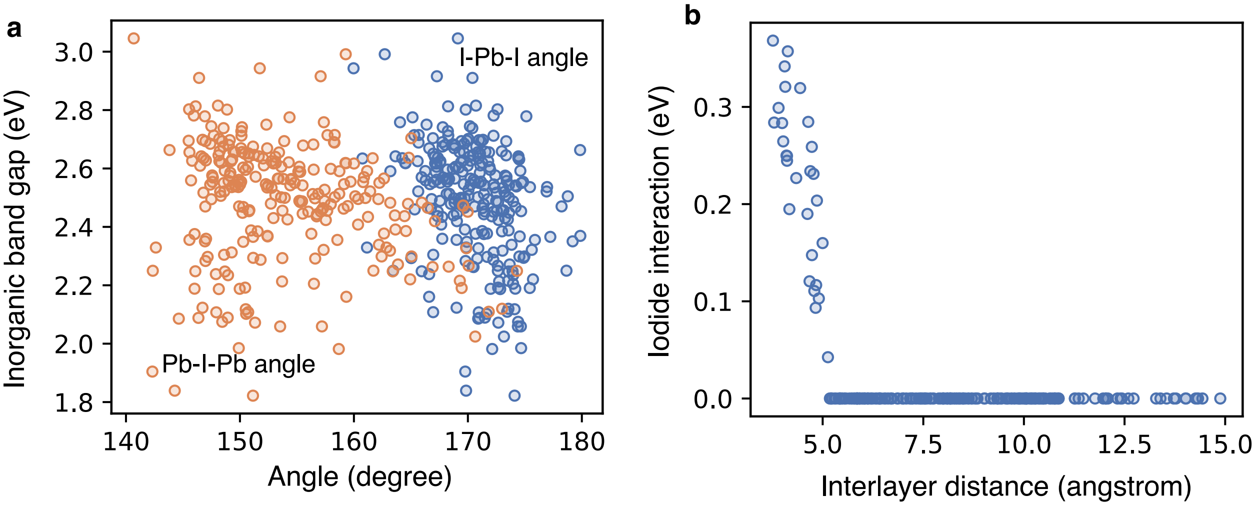

Figure S12: Influence of organic spacers on inorganic band edge. The modification of inorganic energy levels is driven by two primary factors. **a** Inorganic energy gap at the Γ point, influenced by octahedral distortions, including the tilting (Pb-I-Pb angle) and internal distortion (I-Pb-I angle) of the PbI_6 framework, leading to energy changes of approximately 1 eV. **b** energy dispersion, determined by interactions between neighbouring iodide atoms when the interlayer distance is below 5.0 Å, resulting in similar energy changes (approximately 1 eV). Dispersion is quantified by the energy difference between the Γ and Z points.

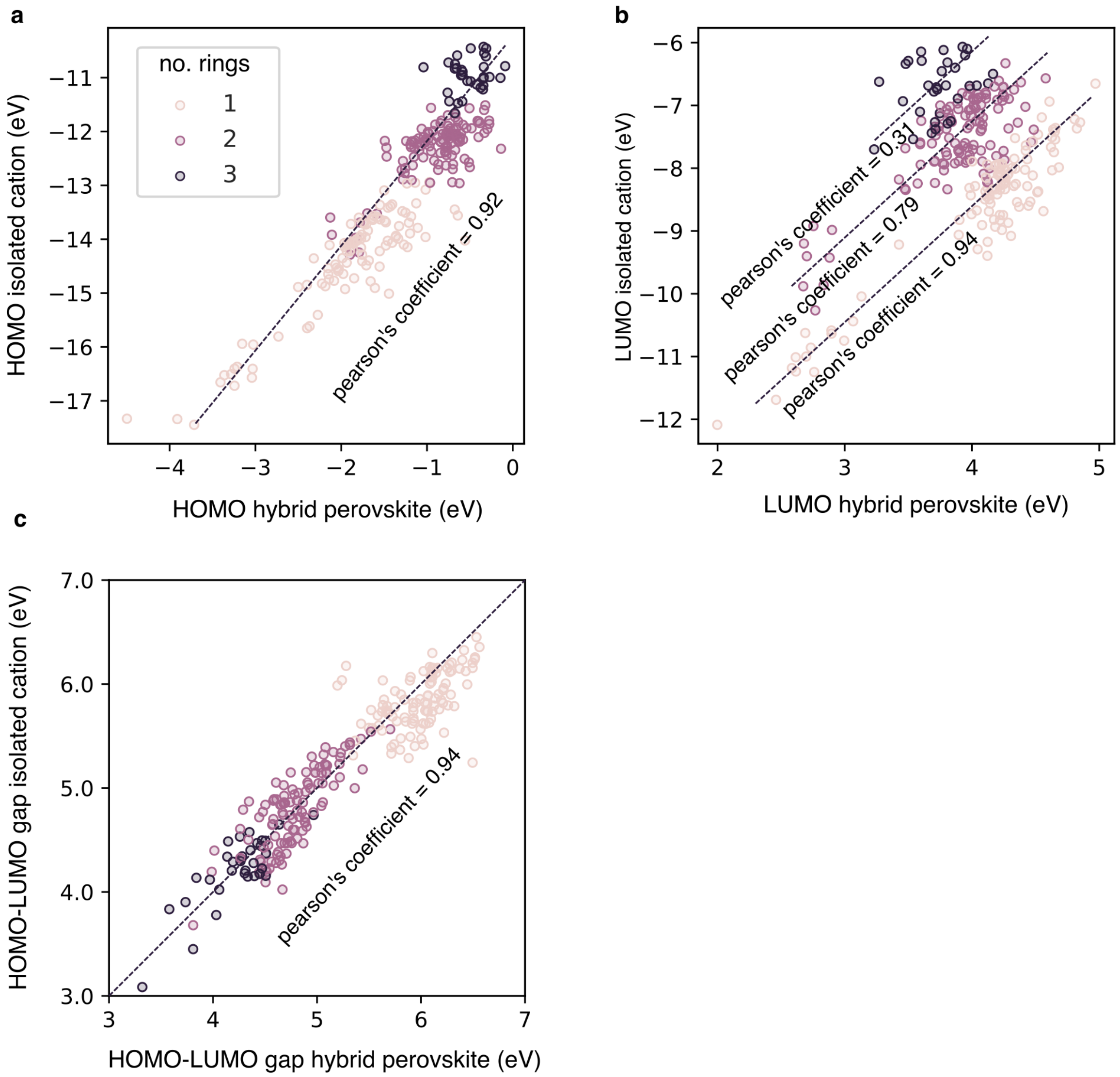

Figure S13: Correlation between organic frontier levels in hybrid perovskites and their isolated molecular forms. **a** HOMO levels show a strong linear correlation between hybrid perovskites and their corresponding isolated cations, with a Pearson correlation coefficient of 0.92. **b** LUMO levels also follow a linear trend within groups of organic spacers categorized by ring count. The correlation decreases as the ring count increases, with Pearson coefficients of 0.94 (one ring), 0.79 (two rings), and 0.31 (three rings). As discussed later, energy level alignment in multi-ring systems increasingly depends on full DFT validation due to the greater conformational variability of larger organic spacers. **c** The HOMO–LUMO gap remains consistent between the two calculation methods, with a correlation coefficient of 0.94.

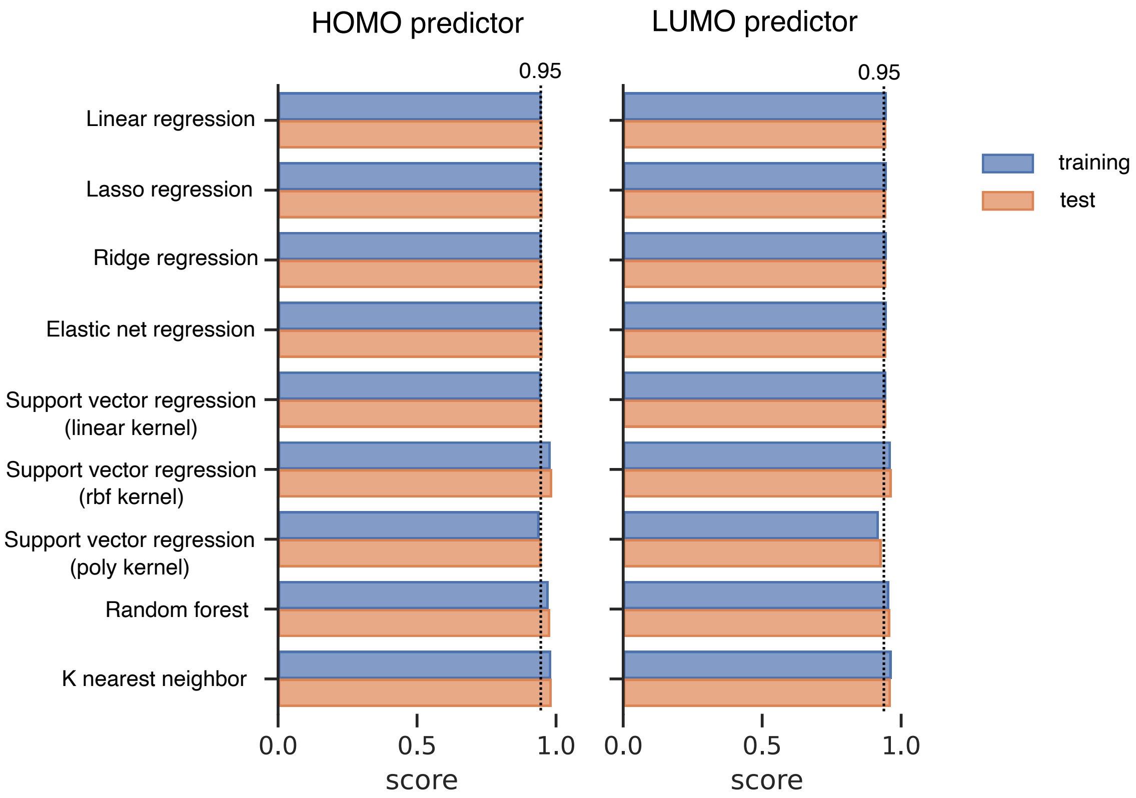

Figure S14: Comparison of training/test scores of various ML models for HOMO/LUMO prediction. No overfitting was detected, as evidenced by comparable performance on both training and test sets.

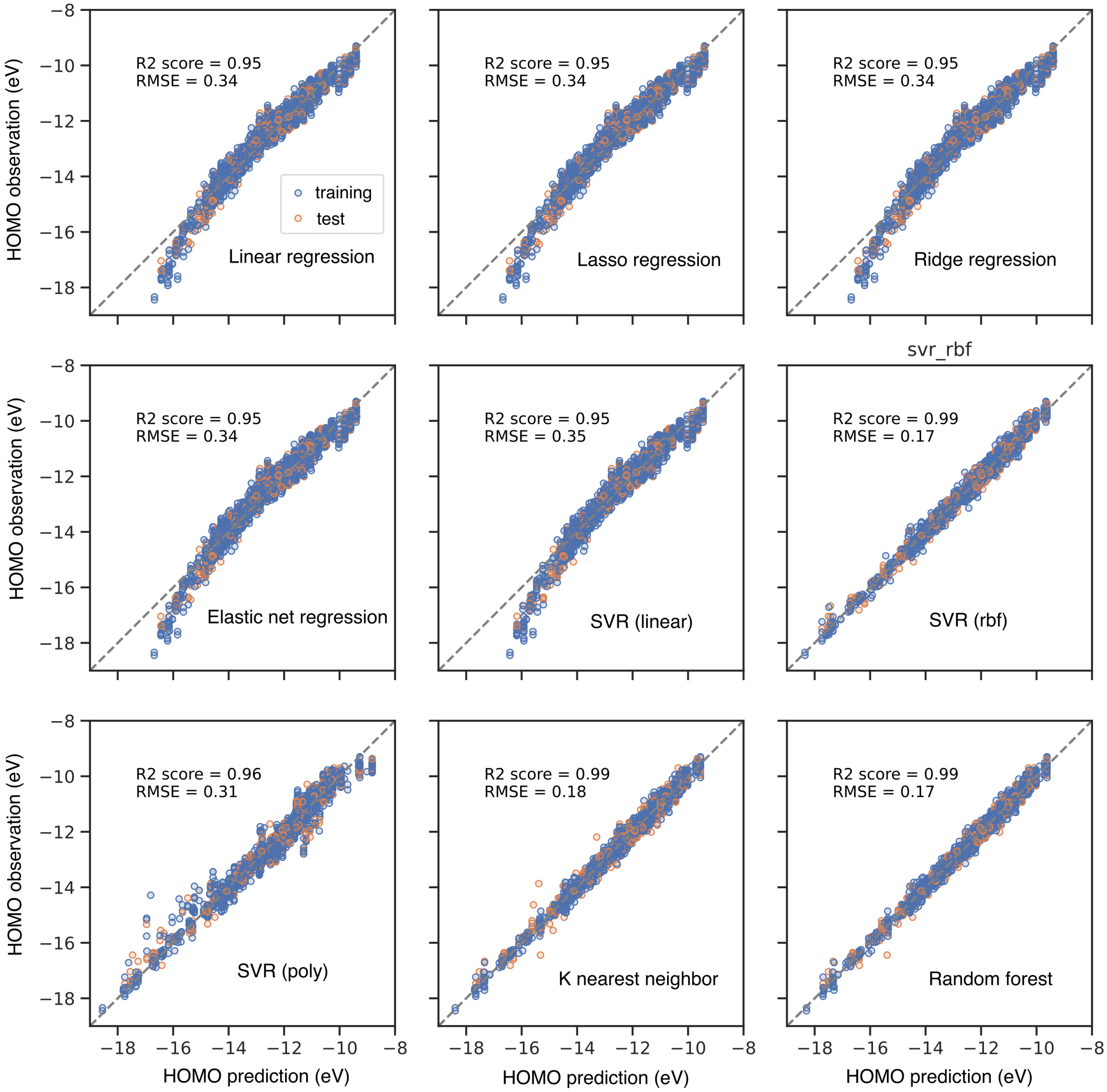

Figure S15: Predicted vs. true values for HOMO level across various ML models.

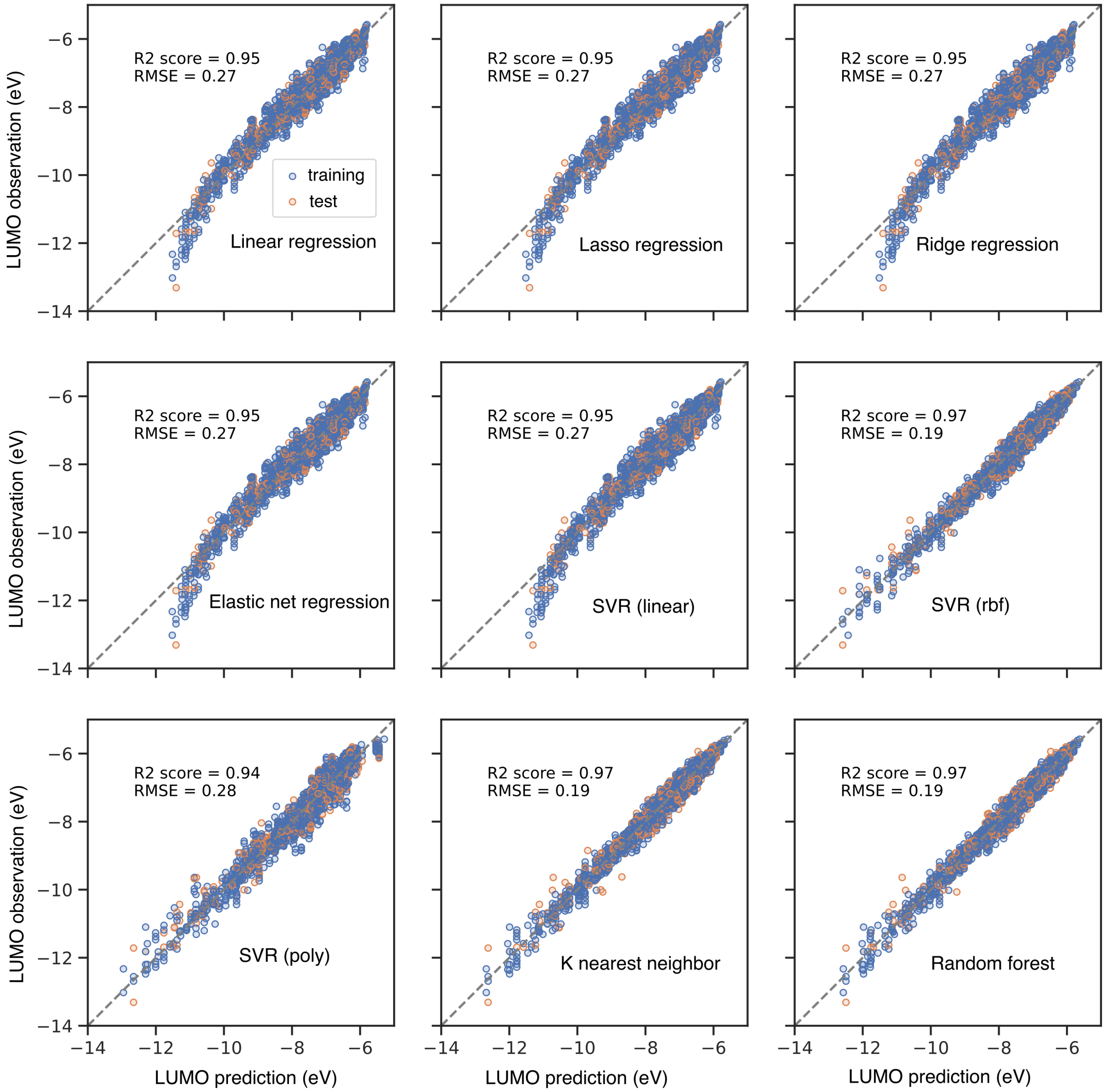

Figure S16: Predicted vs. true values for LUMO level across various ML models.

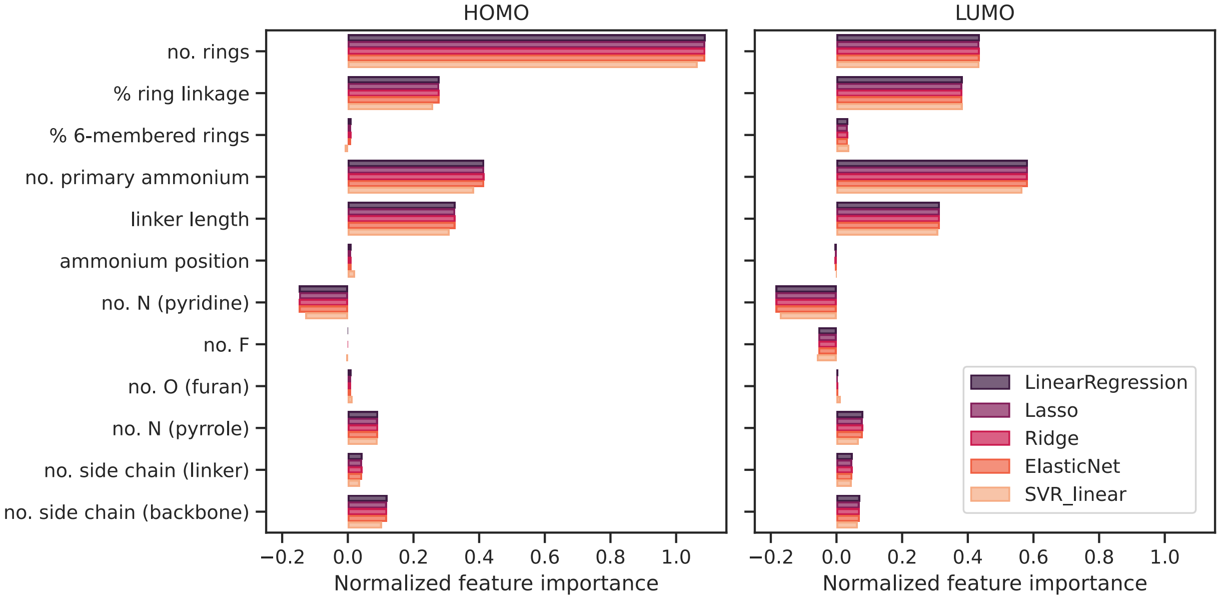

Figure S17: Normalized feature coefficients of all linear models used in this work. The normalized coefficients represent the change in the target variable resulting from a one-standard-deviation change in organic descriptors, enabling intuitive comparison of feature importance across different scales. All linear models exhibit similar performance in terms of feature coefficients, indicating comparable behaviour. LASSO regression was selected due to its L1 regularization, which favours solutions with fewer non-zero coefficients, effectively identifying key features and reducing model complexity.

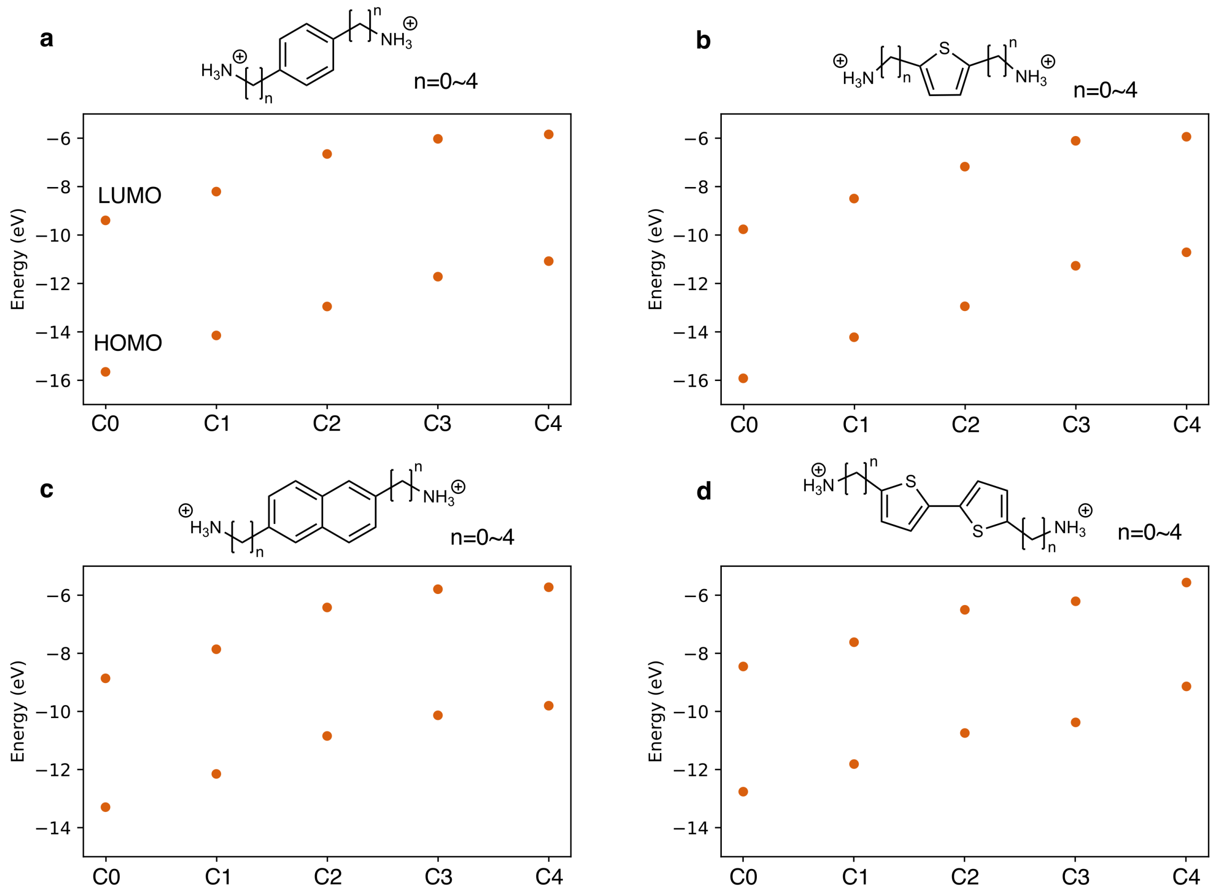

Figure S18: Effect of linker length on the frontier energy levels of four representative organic cations. HOMO and LUMO energies are shown for **a** benzene-based, **b** thiophene-based, **c** naphthalene-based, and **d** bi-thiophene-based backbones as the number of carbon atoms in the tethering ammonium is increased from C0 to C4. For most backbones, the upward shift in frontier energy levels induced by increasing linker length rapidly saturates when extending the linker to propyl (C3) and butyl (C4), except for bi-thiophene-based spacers. Overall, for most organic spacers, while increasing linker length influences the frontier levels of the organic cation, its impact progressively diminishes as the linker becomes longer.

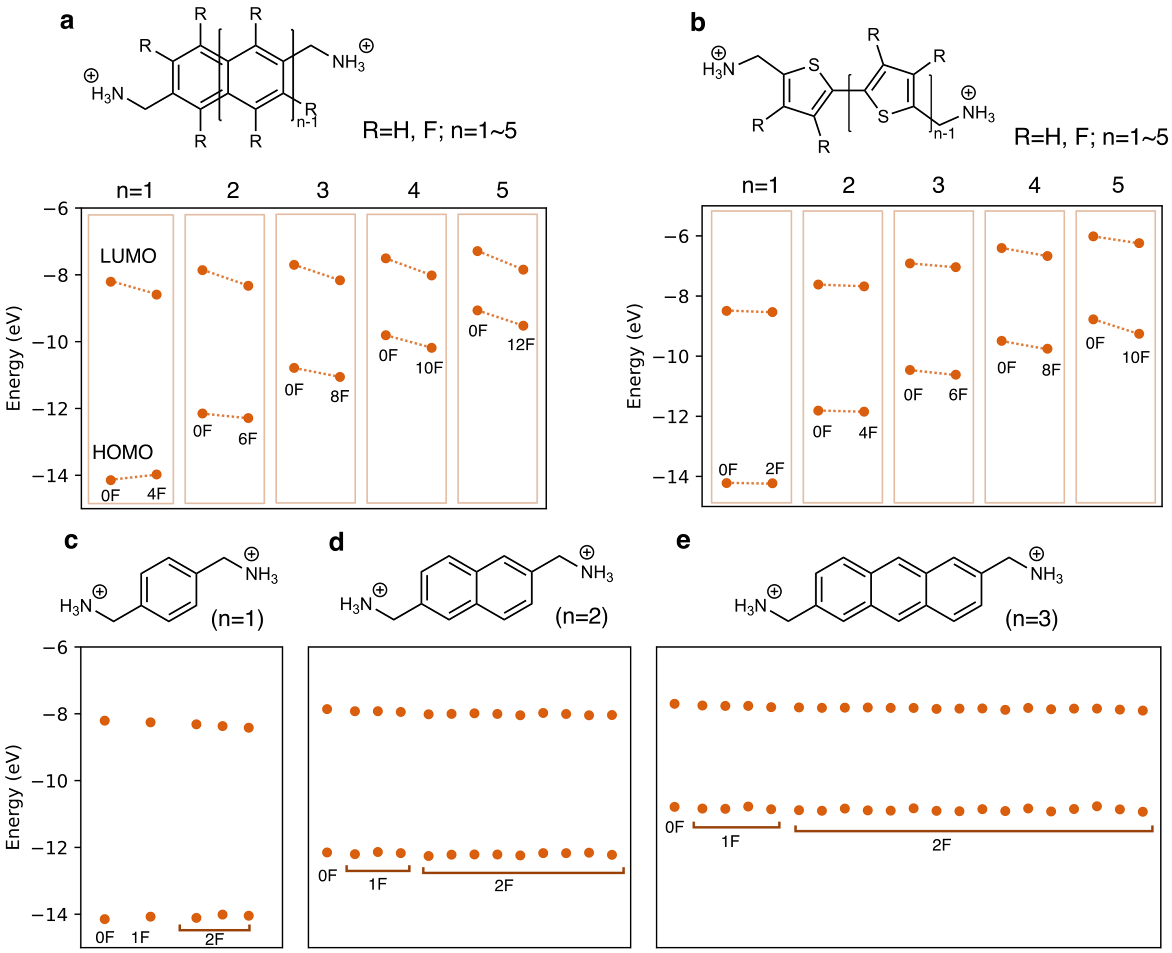

Figure S19: Effect of fluorination on the frontier energy levels of representative organic cations. Comparison of non-fluorinated and fully (peri-)fluorinated **a** acene-based, and **b** oligothiophene-based spacer cations with increasing conjugation length. **c-e** Structural isomers of acene-based spacer cations with fluorine substitution at different positions on the aromatic backbone. Single-site fluorination leads to only minor shifts in the HOMO and LUMO energies, whereas extensive fluorination results in a substantially larger lowering of both frontier levels, consistent with the previous report (*73*). Within this subset, no clear or systematic dependence of frontier energy levels on the backbone classes and substitution position was observed. These results suggest that fluorination can effectively tune frontier energy levels primarily when multiple fluorine atoms are introduced, while the effect of single-site fluorination is, on average, weak across the backbone classes considered.

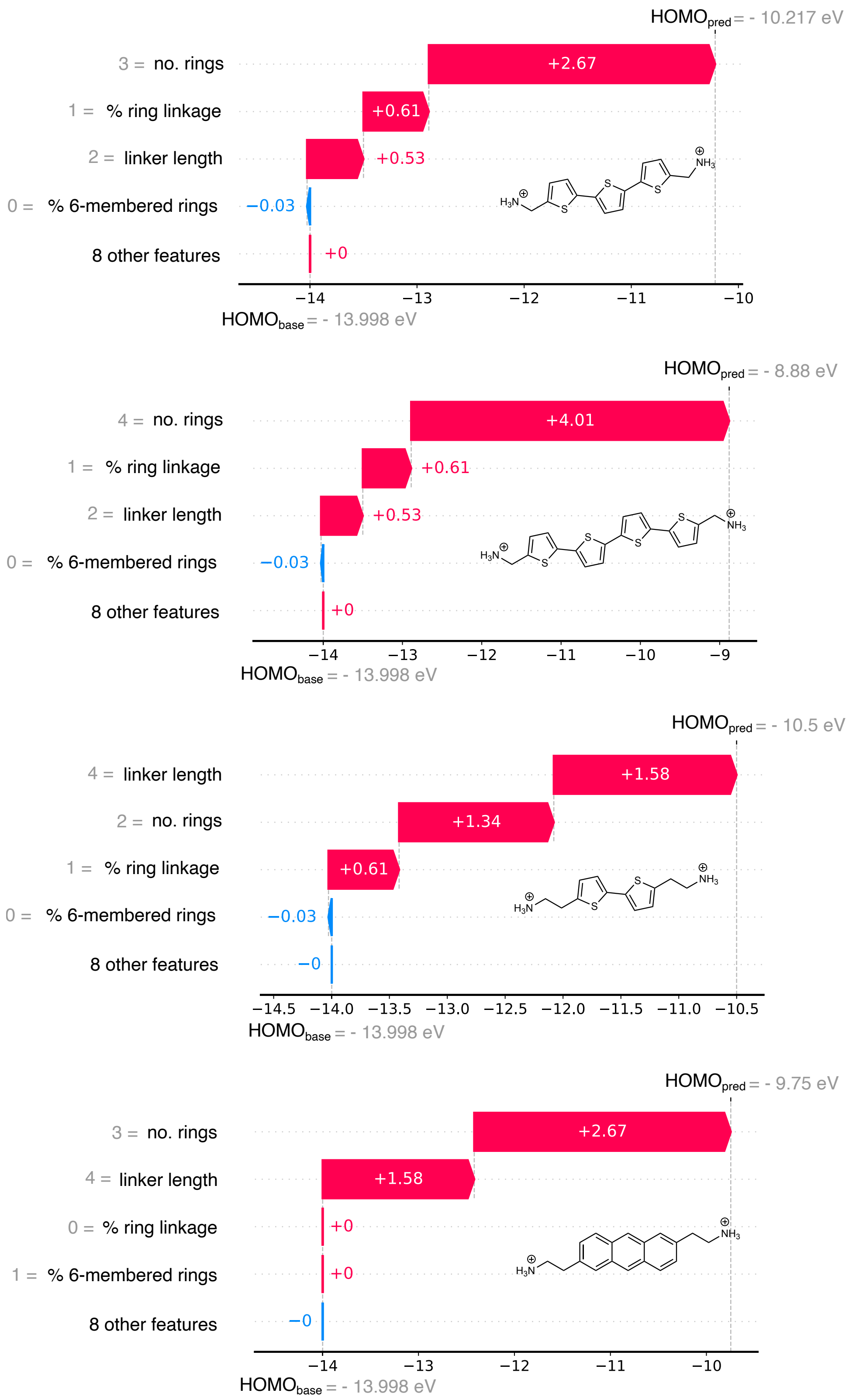

Figure S20: SHAP value analysis of representative organic spacers in type IIa. The SHAP values are calibrated using the G_0 molecule as a baseline.

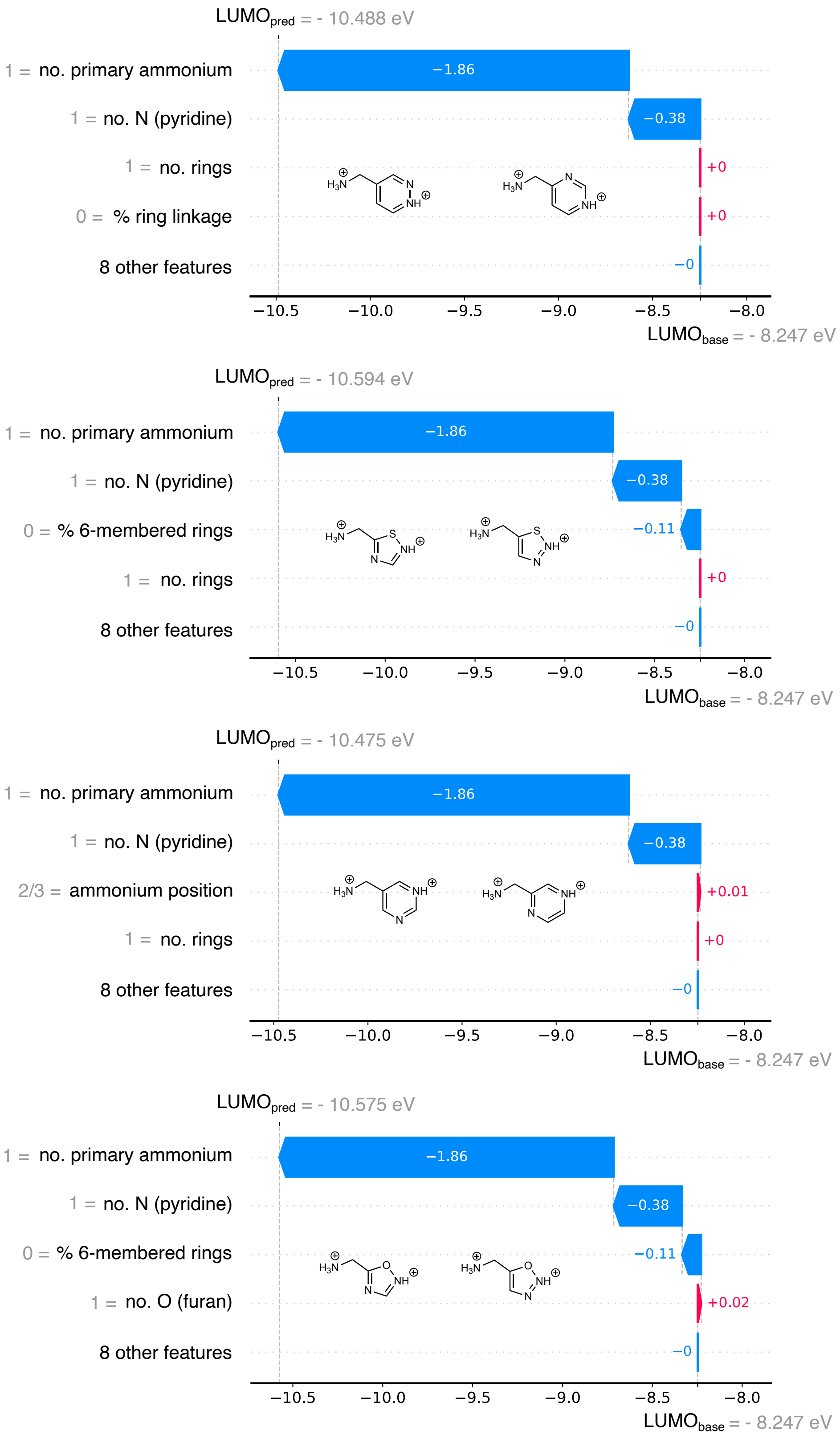

Figure S21: SHAP value analysis of representative organic spacers in type IIb. The SHAP values are calibrated using the G_0 molecule as a baseline.

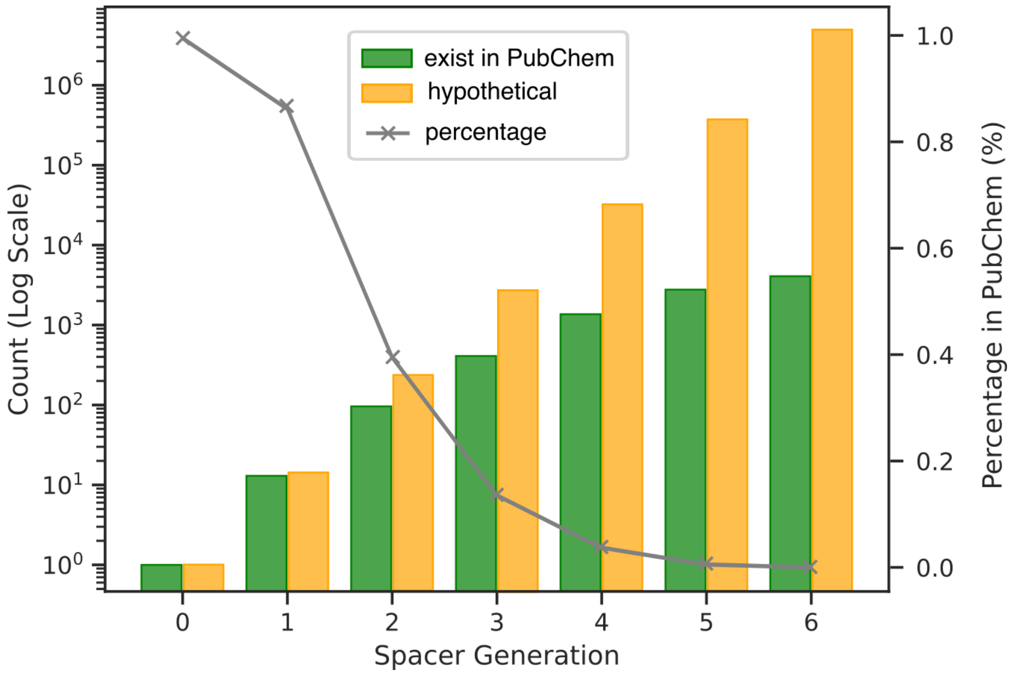

Figure S22: Number of generated organic spacers vs. spacers existing in PubChem in G0-G6.

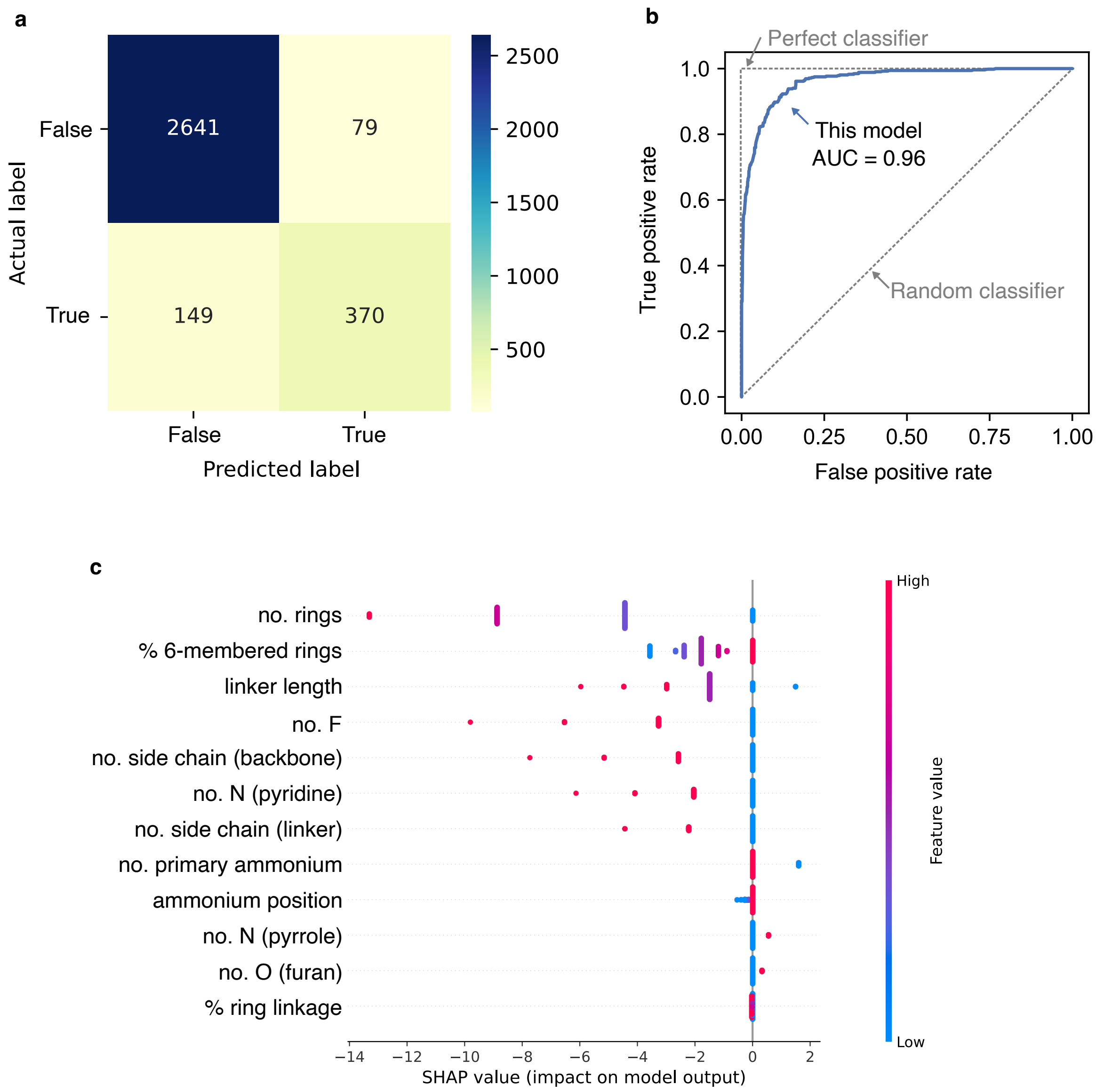

Figure S23: Machine learning analysis of the relationship between fingerprints and PubChem existence. A logistic regression model was employed to reveal the relationship between molecular fingerprints and the likelihood of existence in the PubChem database, achieving **a** an accuracy of 0.93 and **b** an AUC of 0.96. These metrics demonstrate the model's reliability in capturing this relationship. **c** Key influencing features include the number of aromatic rings, which has the most significant impact, followed by side chains and heteroatom substitutions such as fluorination and pyridine-type nitrogen.

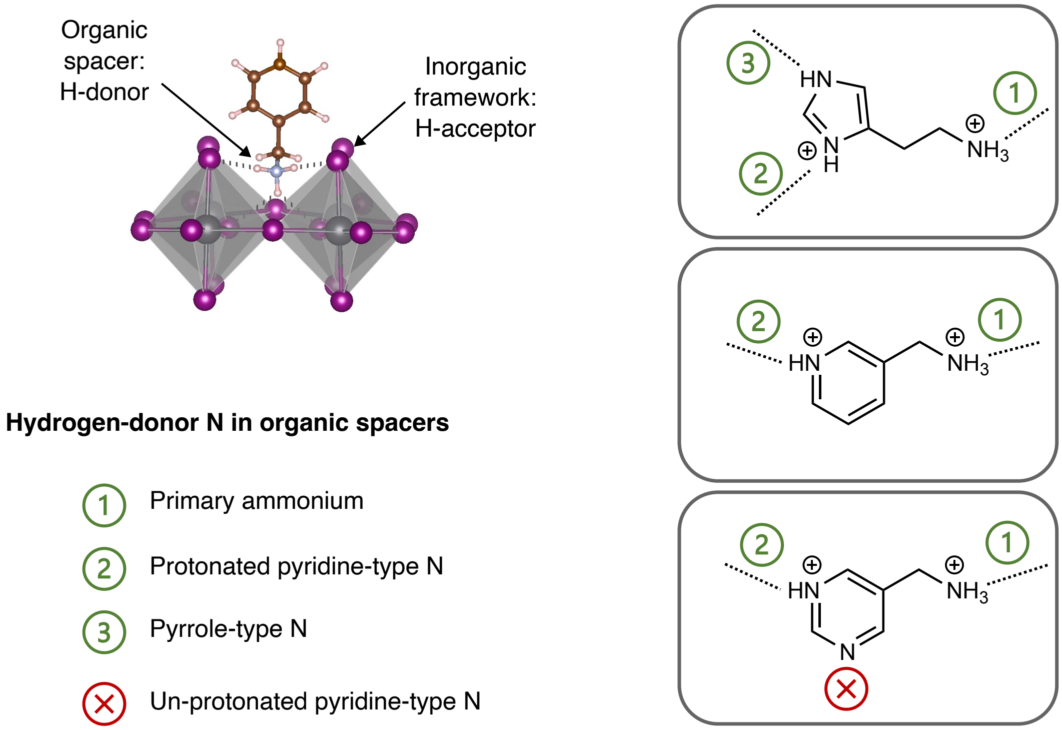

Figure S24: Hydrogen-donor nitrogen for hydrogen bond formation in 2D perovskite. This study involves four types of nitrogens present in organic spacers. Among these, only three can act as hydrogen donors: (1) Primary ammonium ($-NH_3^+$) (2) Protonated pyridine-type nitrogen ($-NH^+-$) (3) Pyrrole-type nitrogen ($-NH-$). Conversely, unprotonated pyridine-type nitrogen ($-N-$) cannot serve as a hydrogen donor, as it lacks a covalently bonded hydrogen.

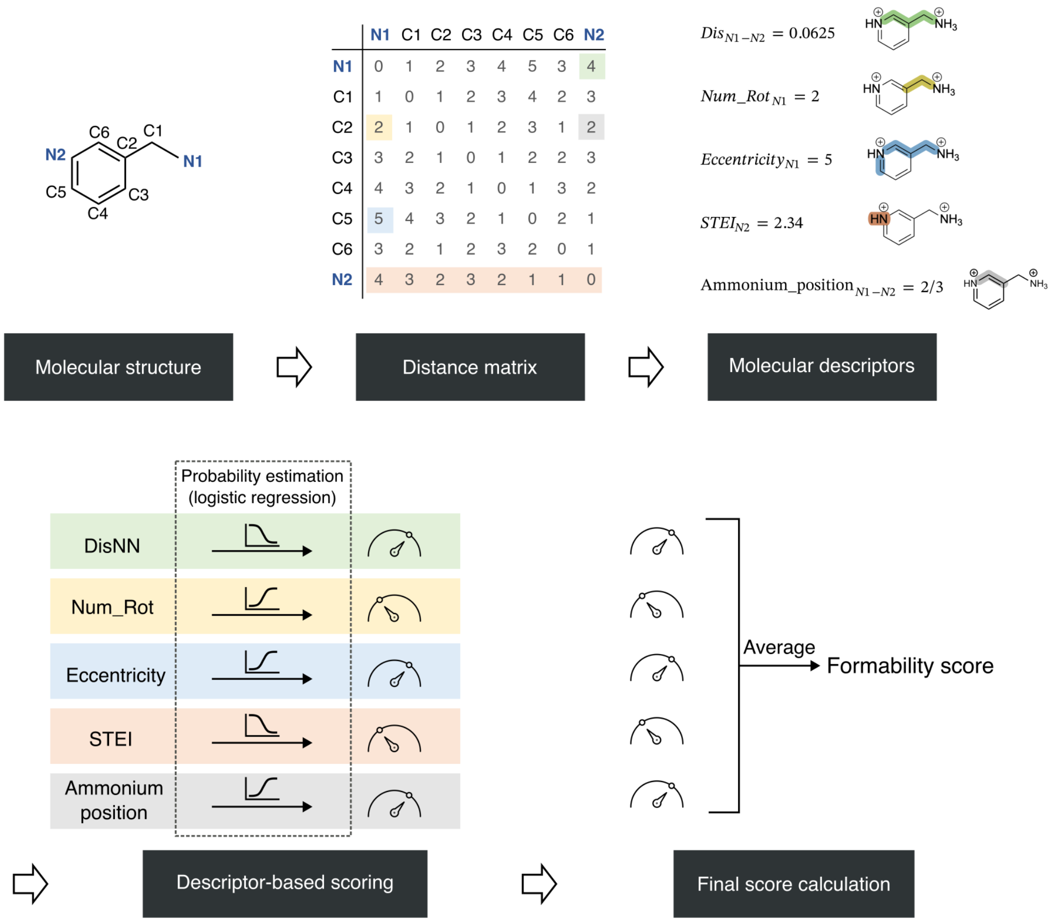

Figure S25: Calculation of formability scores for organic spacers. Organic spacers are first converted into distance matrices, which serve as simplified 2D representations of molecular structure. Five topological descriptors are then computed from these matrices, centered on hydrogen-donor nitrogen atoms within each molecule. Each descriptor is fitted to a logistic regression model to generate an individual score, with model parameters derived from previously reported positive and negative examples. The final formability score is obtained by averaging the individual descriptor scores.

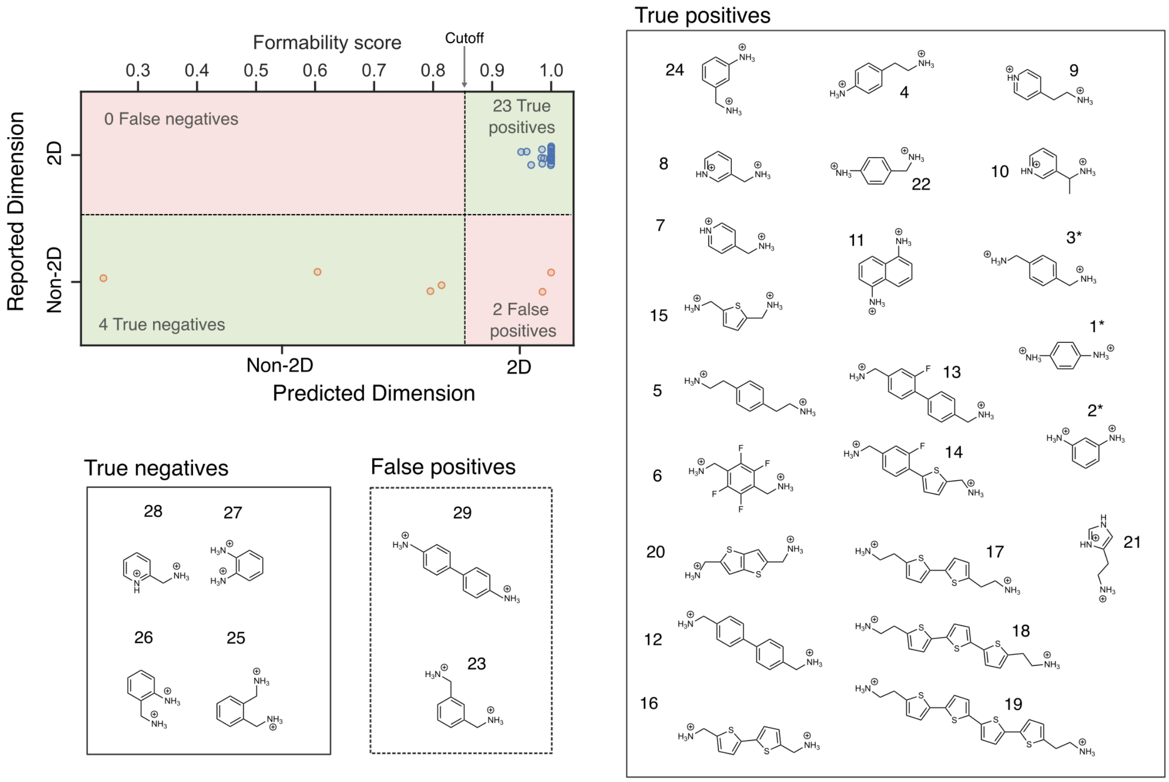

Figure S26: Accuracy of formability scores for reported organic spacers. Confusion matrix comparing the reported dimensionality of organic cations with their predicted outcomes based on formability scores. Among the true positives, three spacers (marked with *) have been reported to form both 2D and non-2D phases depending on reaction conditions. Two false positives–spacers reported as forming only non-2D phases–were predicted to form 2D phases. Due to the limited number of reported non-2D cases, data scarcity remains a challenge. These two false positives may, under carefully controlled conditions, still be capable of forming 2D phases.

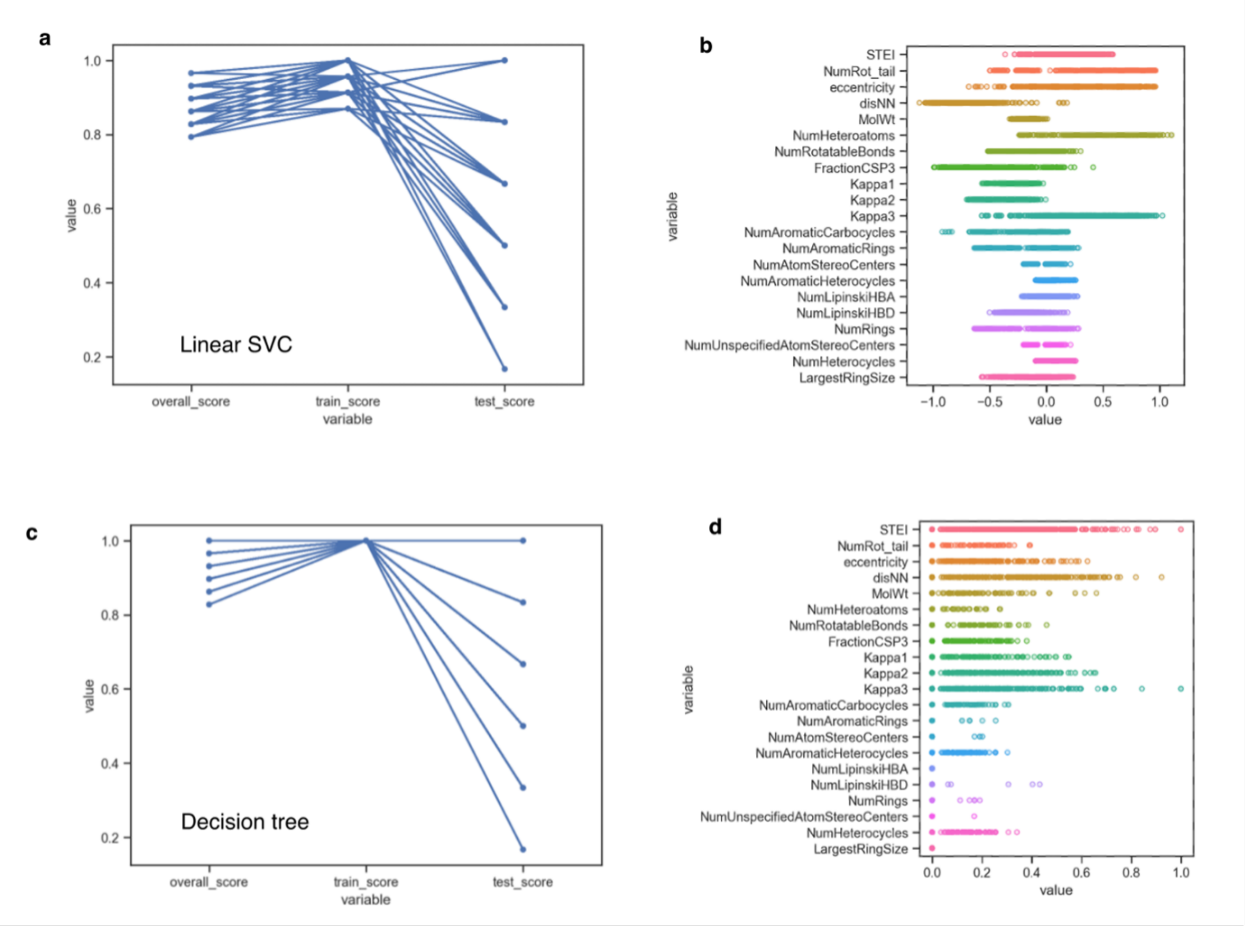

Figure S27: Comparison of our formability score scheme with machine learning methods.

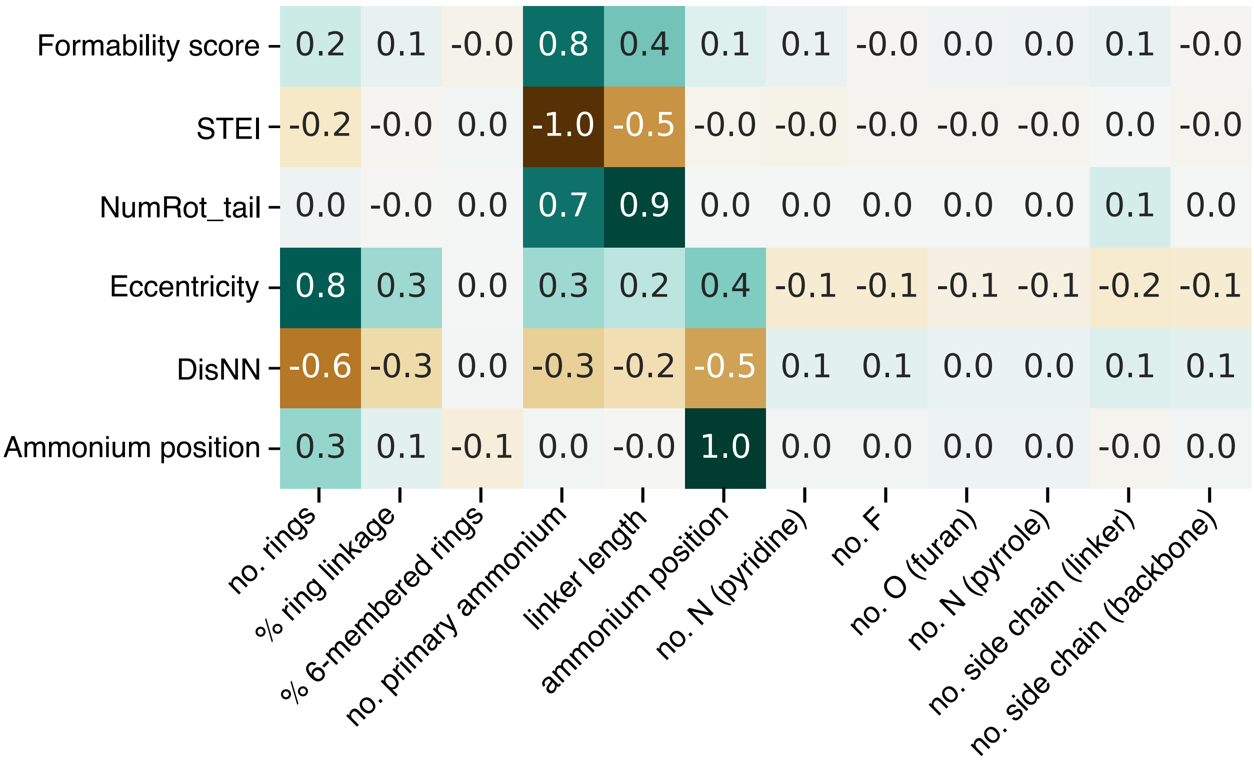

Figure S28: Pearson's correlation coefficient between formability score and molecular fingerprint.

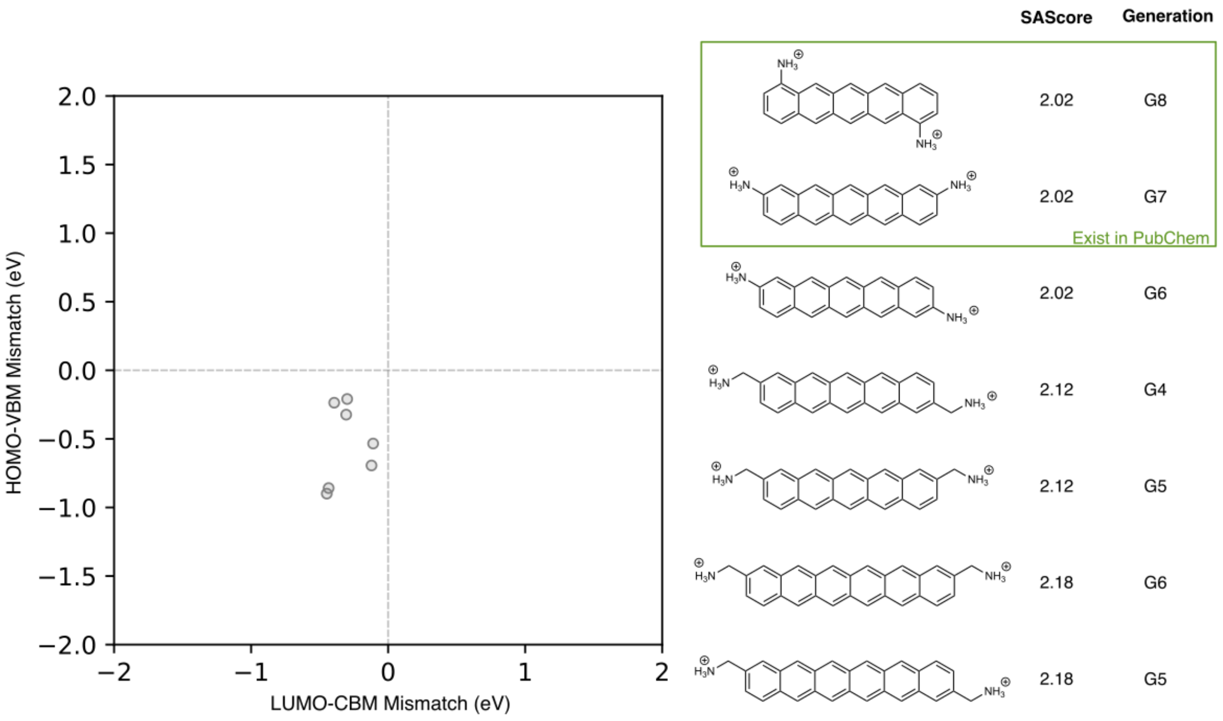

Figure S29: The structures of type I_b organic spacers. The top two organic spacers are the inverse designed final candidates. The other spacers are not listed in the PubChem database, but they are predicted to have high synthetic accessibility due to their relatively low synthesis accessibility score. In general, the *SA score* ranges from 1 to 10, with lower scores indicating easier to synthesis according to their structural complexity.

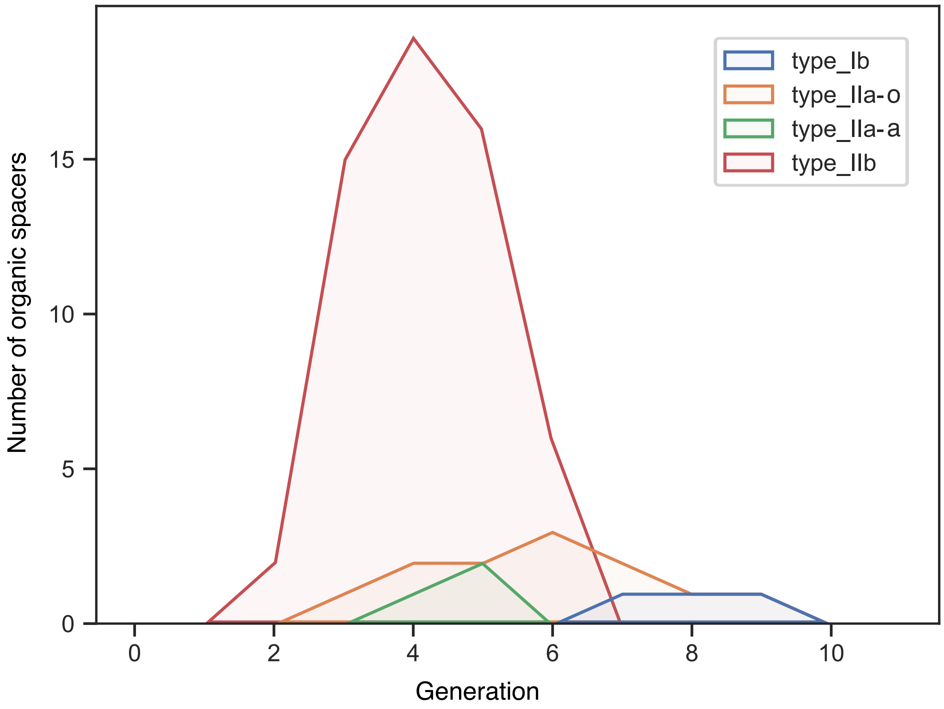

Figure S30: Number of organic spacers discovered in the inverse design phase for targeted energy level alignment type across generations $G_0 - G_{11}$. The number of discovered organic spacers for each fingerprint criterion follows a single-peak distribution across generations: Starts at zero in early generations ($G_0 - G_1$).; Peaks at an intermediate generation ($G_5 - G_9$); Diminishes to zero by G_{11}, indicating that beyond these generations, no additional chemically meaningful spacers are likely to exist within the defined fingerprint constraints.

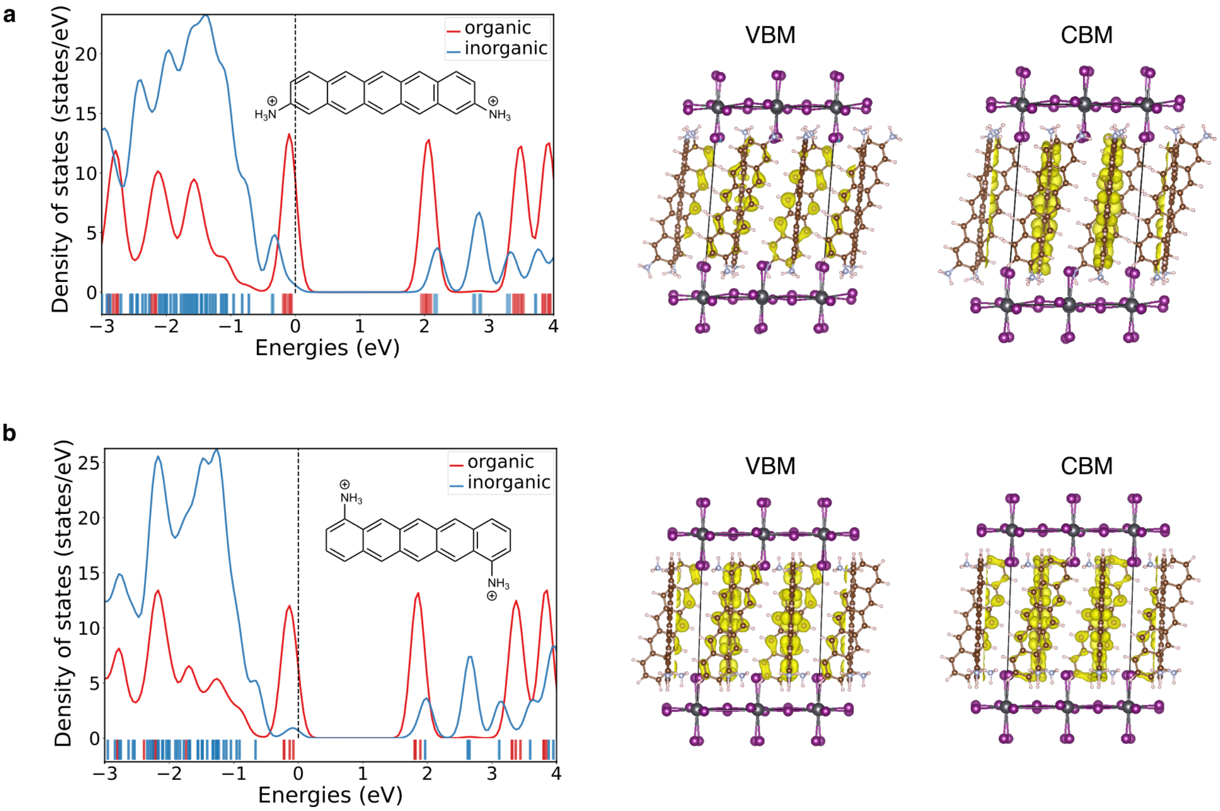

Figure S31: Electronic structure of final candidate selections for type I_b DJ perovskites.

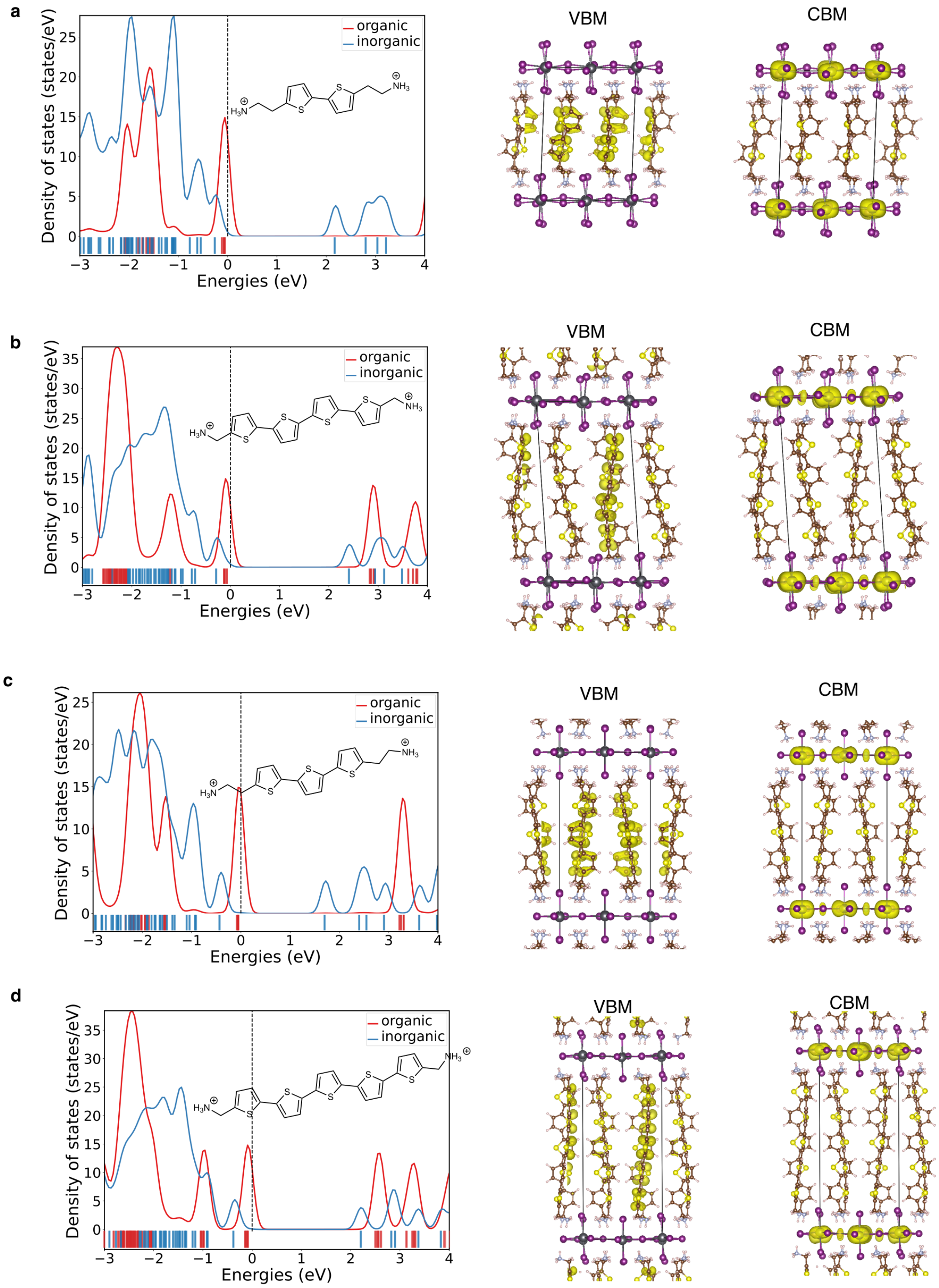

Figure S32: Electronic structure of inverse-designed type II_a DJ perovskites (Part 1).

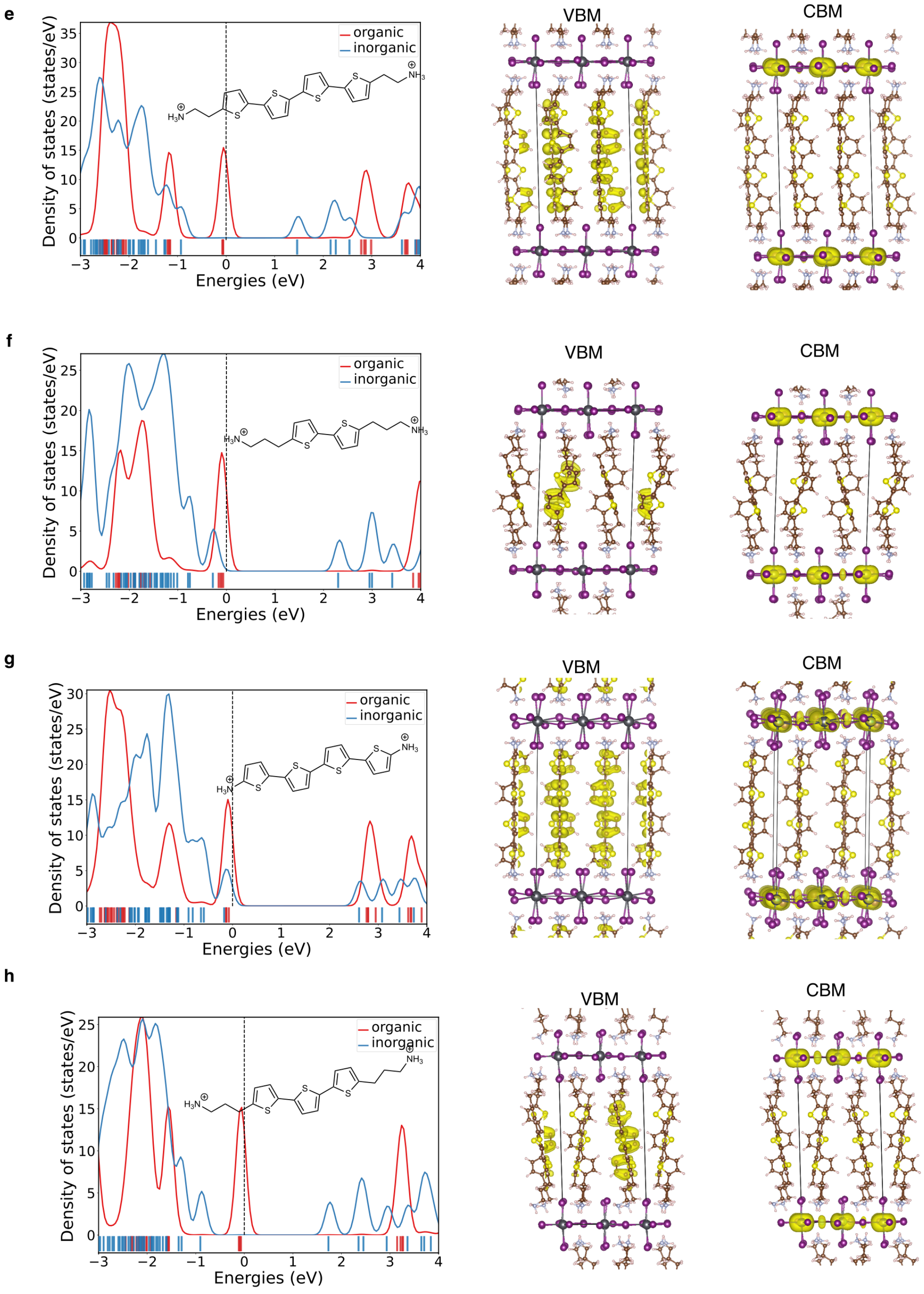

Figure S32: Electronic structure of inverse-designed type II_a DJ perovskites (Part 2, continued).

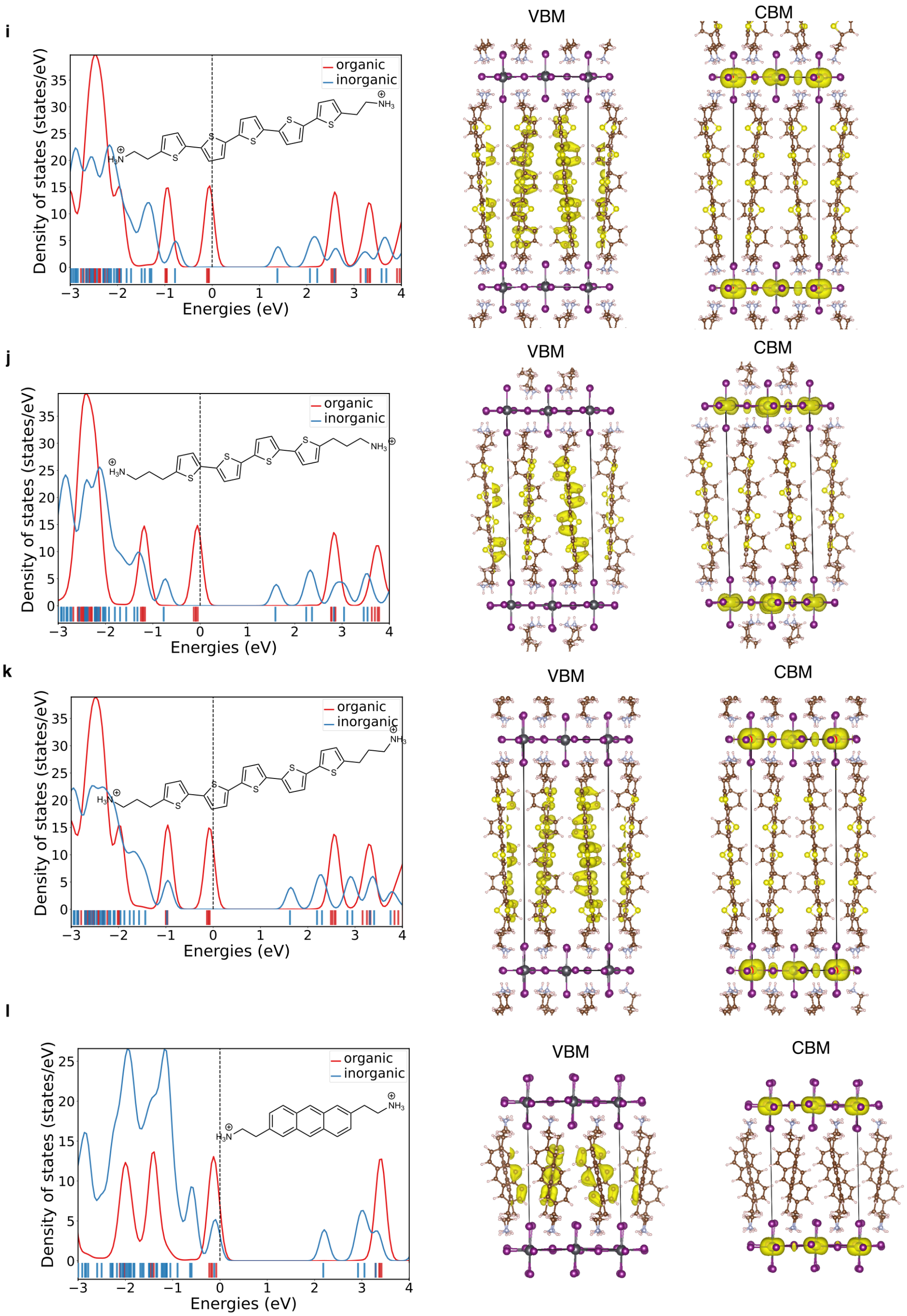

Figure S32: Electronic structure of inverse-designed type II_a DJ perovskites (Part 3, continued).

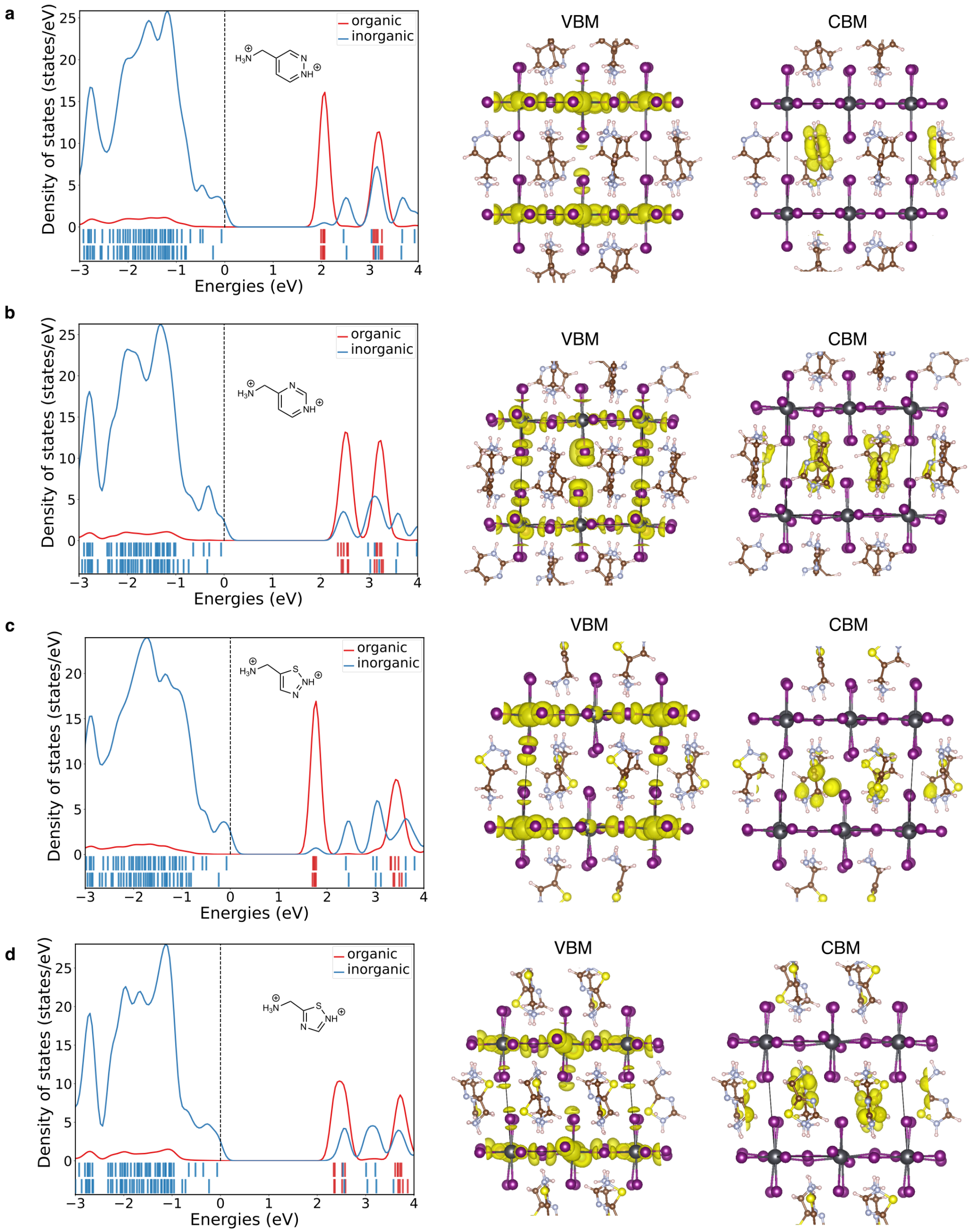

Figure S33: Electronic structure of inverse-designed type II_b DJ perovskites (Part 1).

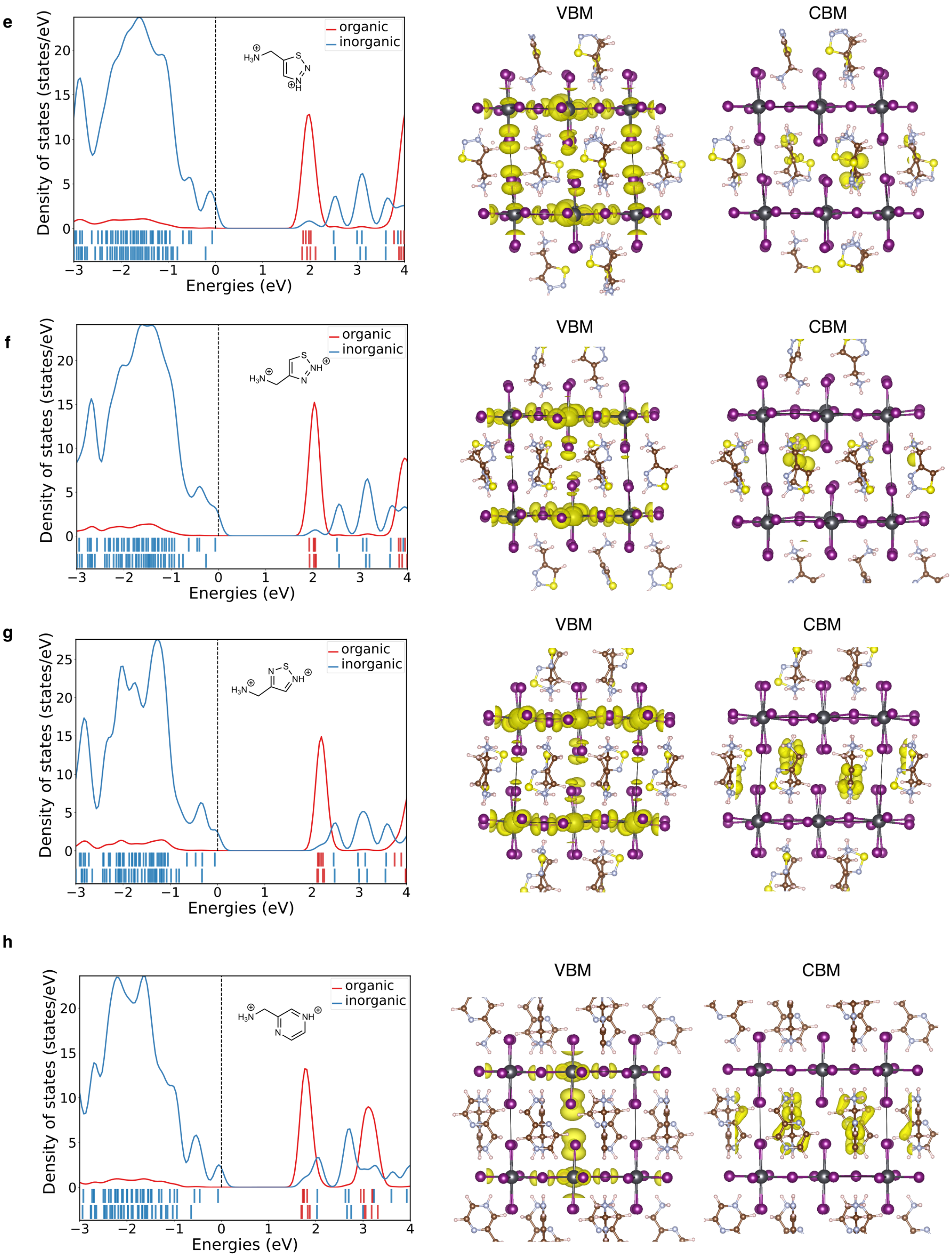

Figure S33: Electronic structure of inverse-designed type II_b DJ perovskites (Part 2, continued).

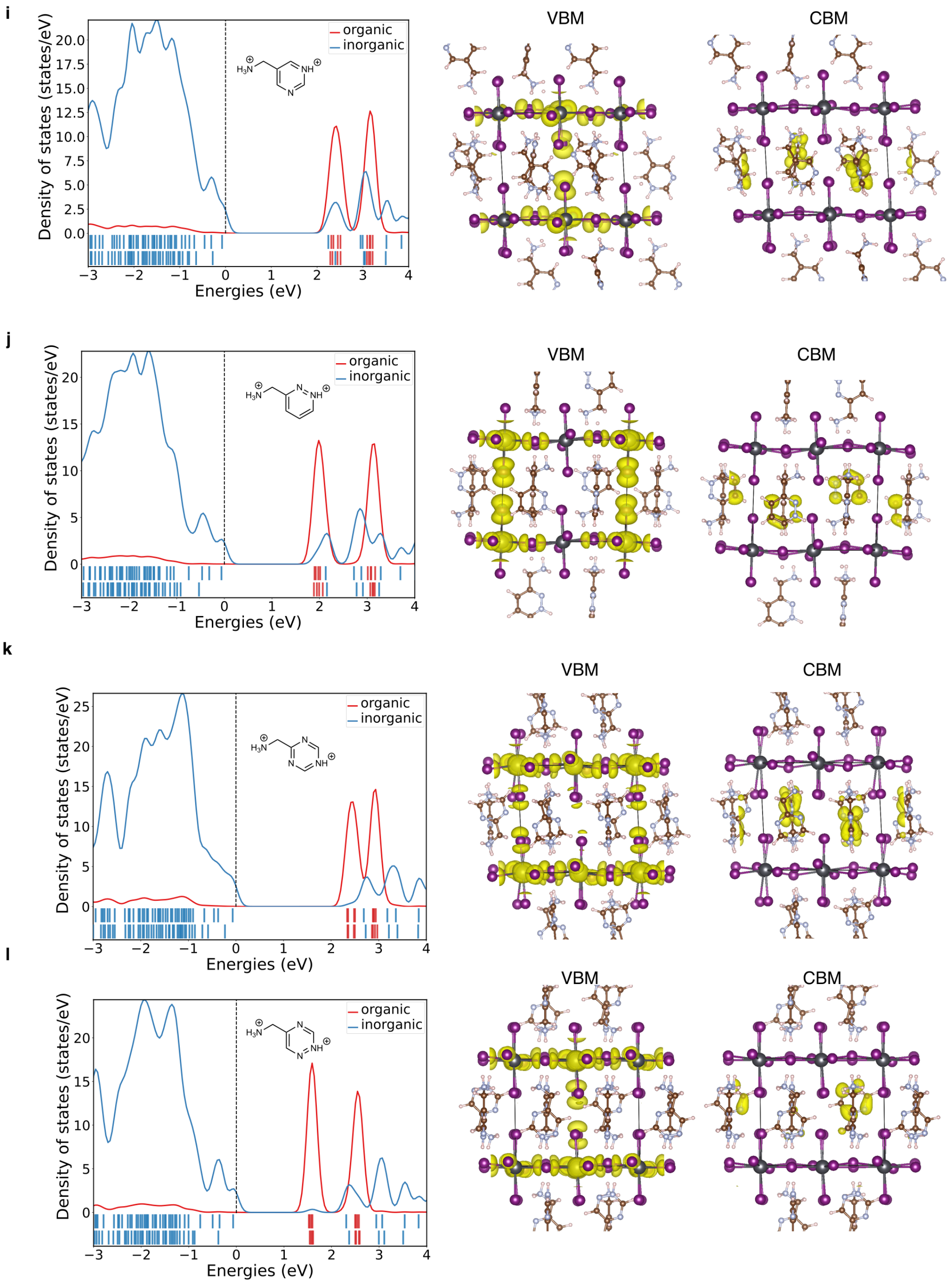

Figure S33: Electronic structure of inverse-designed type II_b DJ perovskites (Part 3, continued).

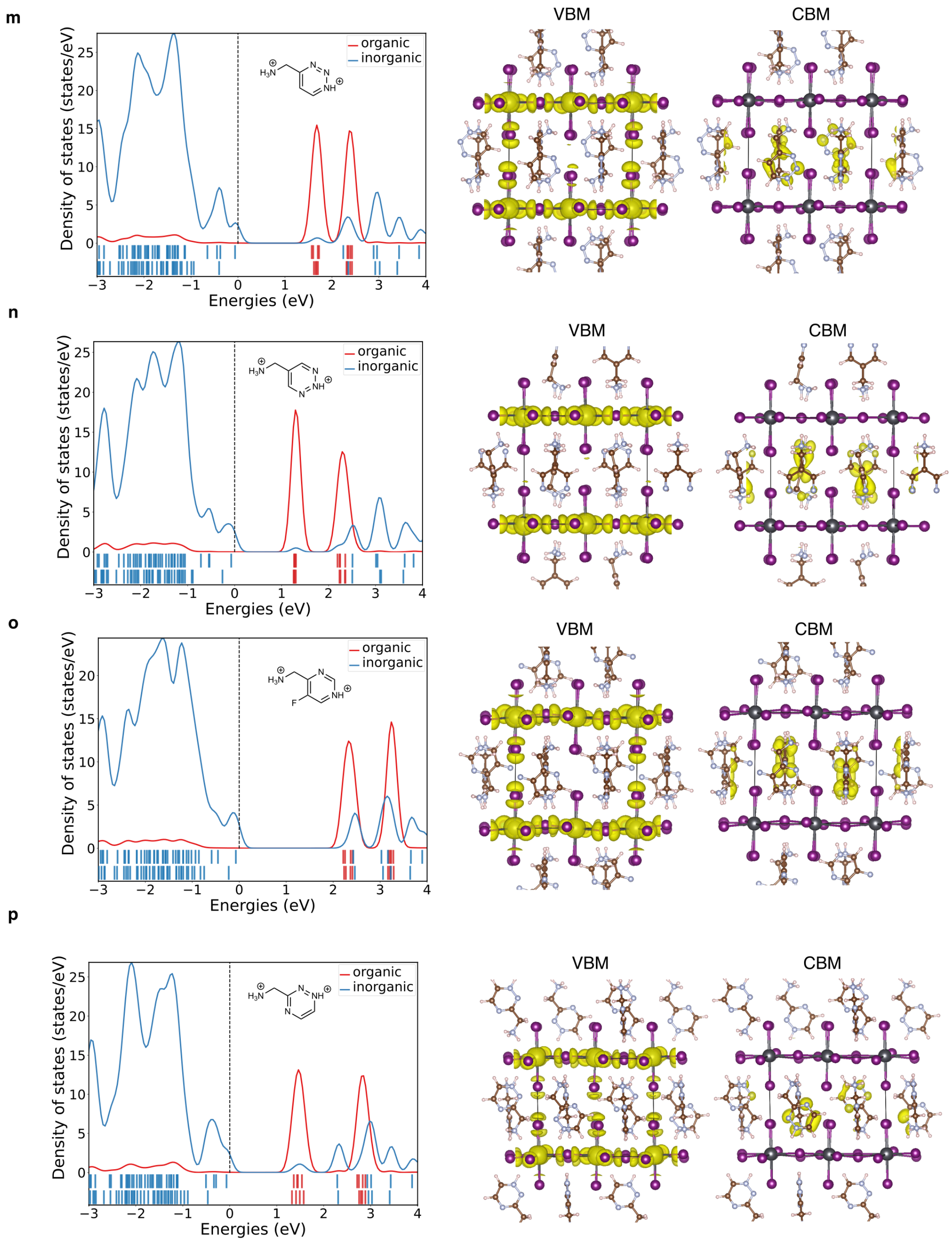

Figure S33: Electronic structure of inverse-designed type II_b DJ perovskites (Part 4, continued).

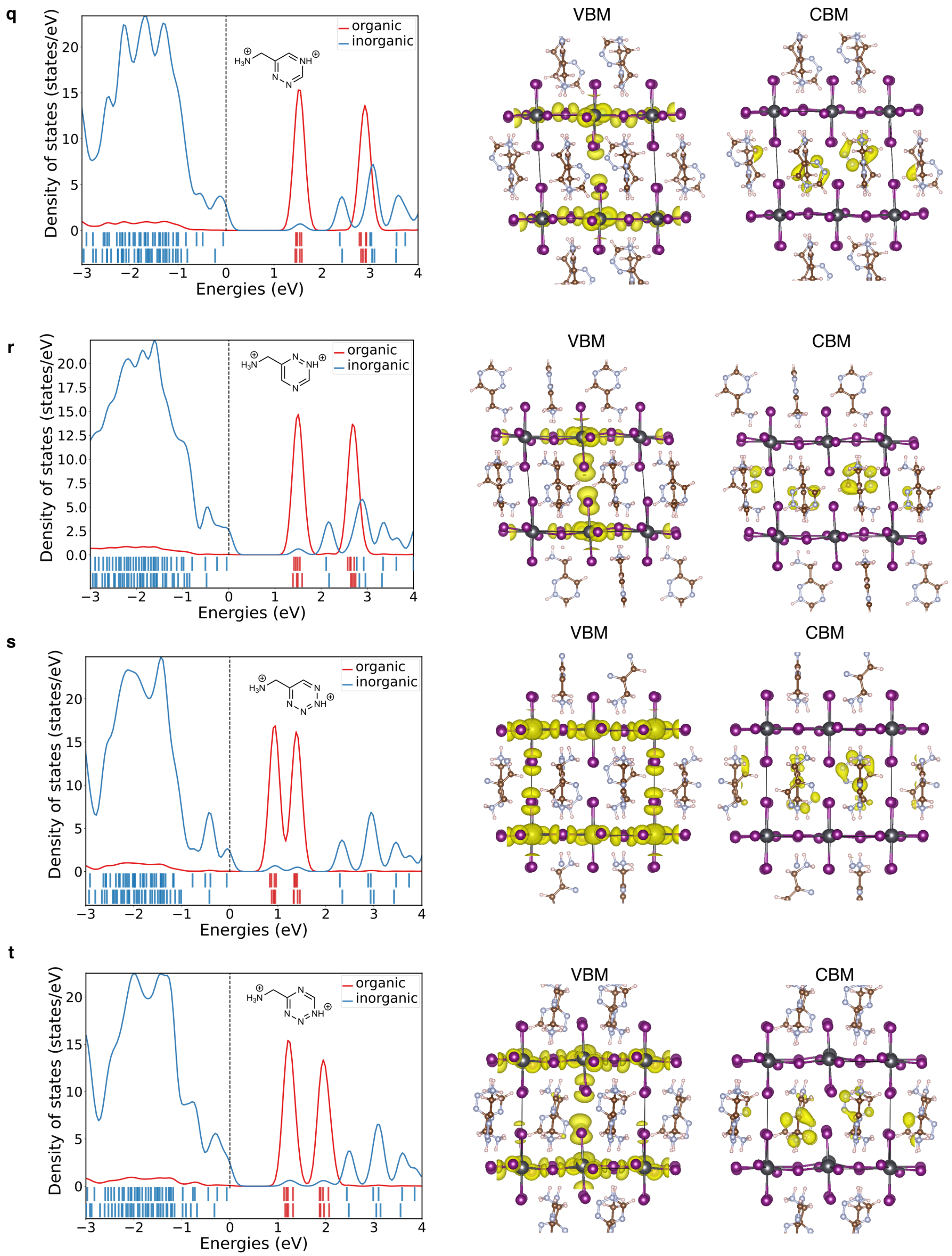

Figure S33: Electronic structure of inverse-designed type II_b DJ perovskites (Part 5, continued).

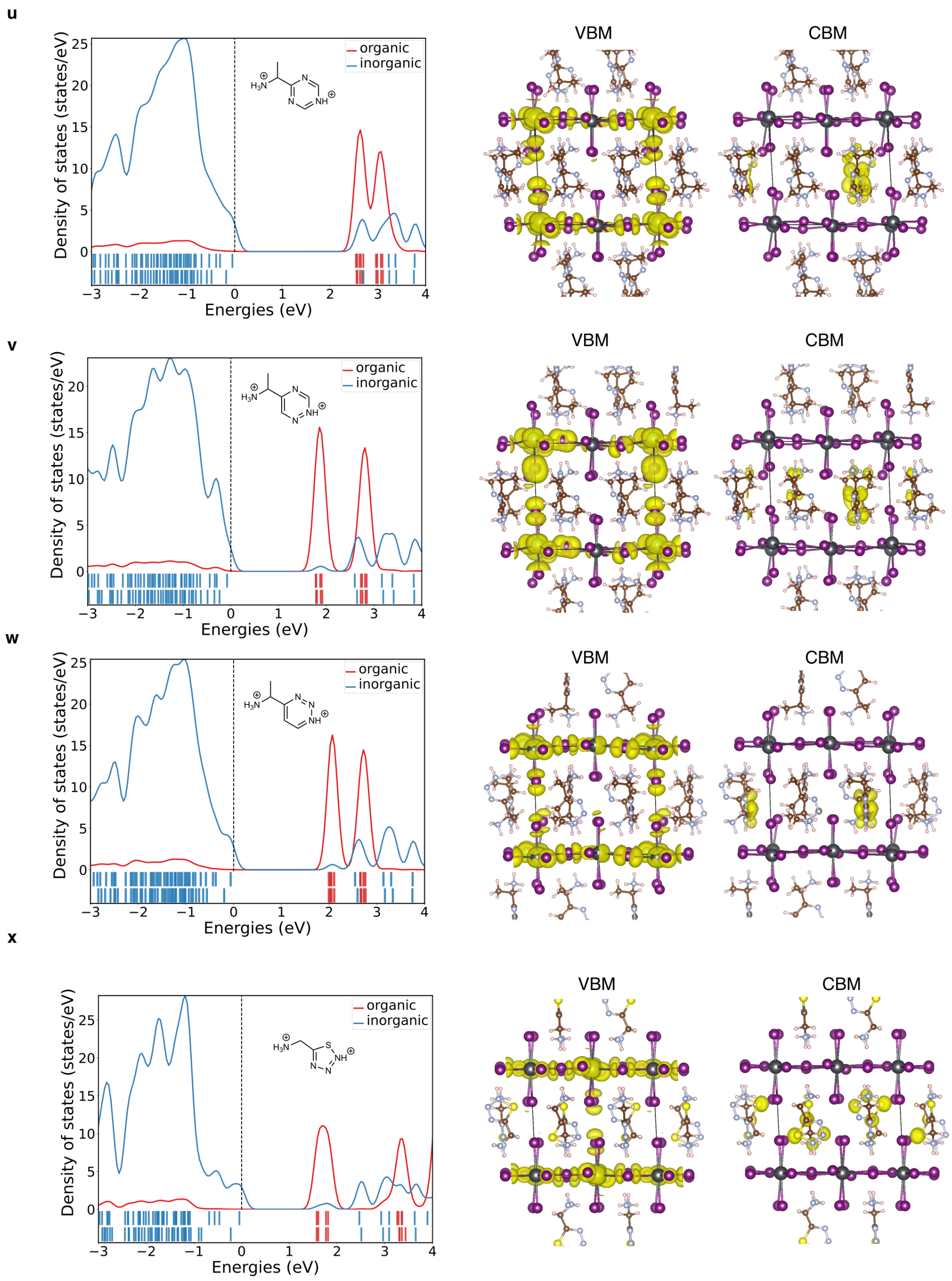

Figure S33: Electronic structure of inverse-designed type II_b DJ perovskites (Part 6, continued).

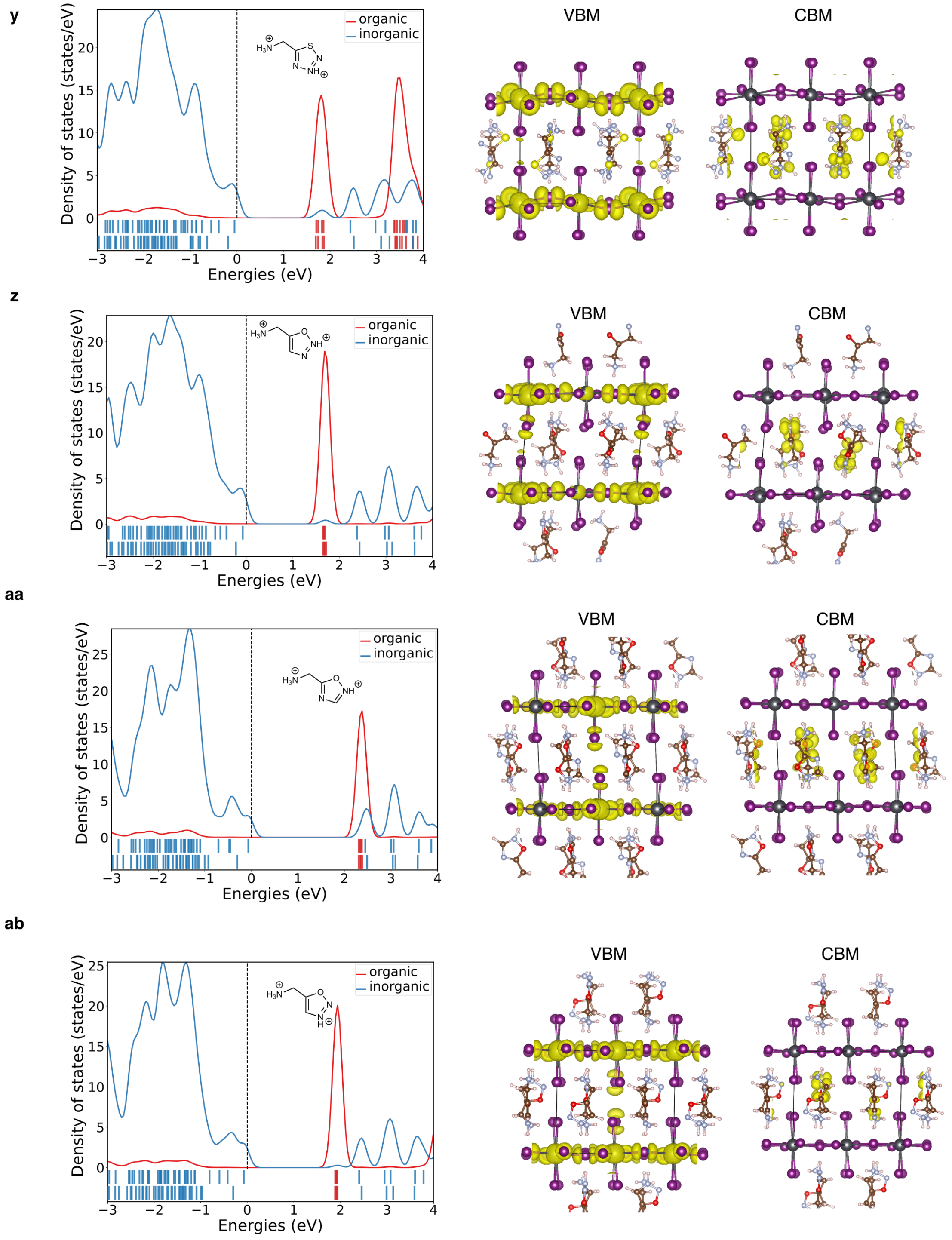

Figure S33: Electronic structure of inverse-designed type II_b DJ perovskites (Part 7, continued).

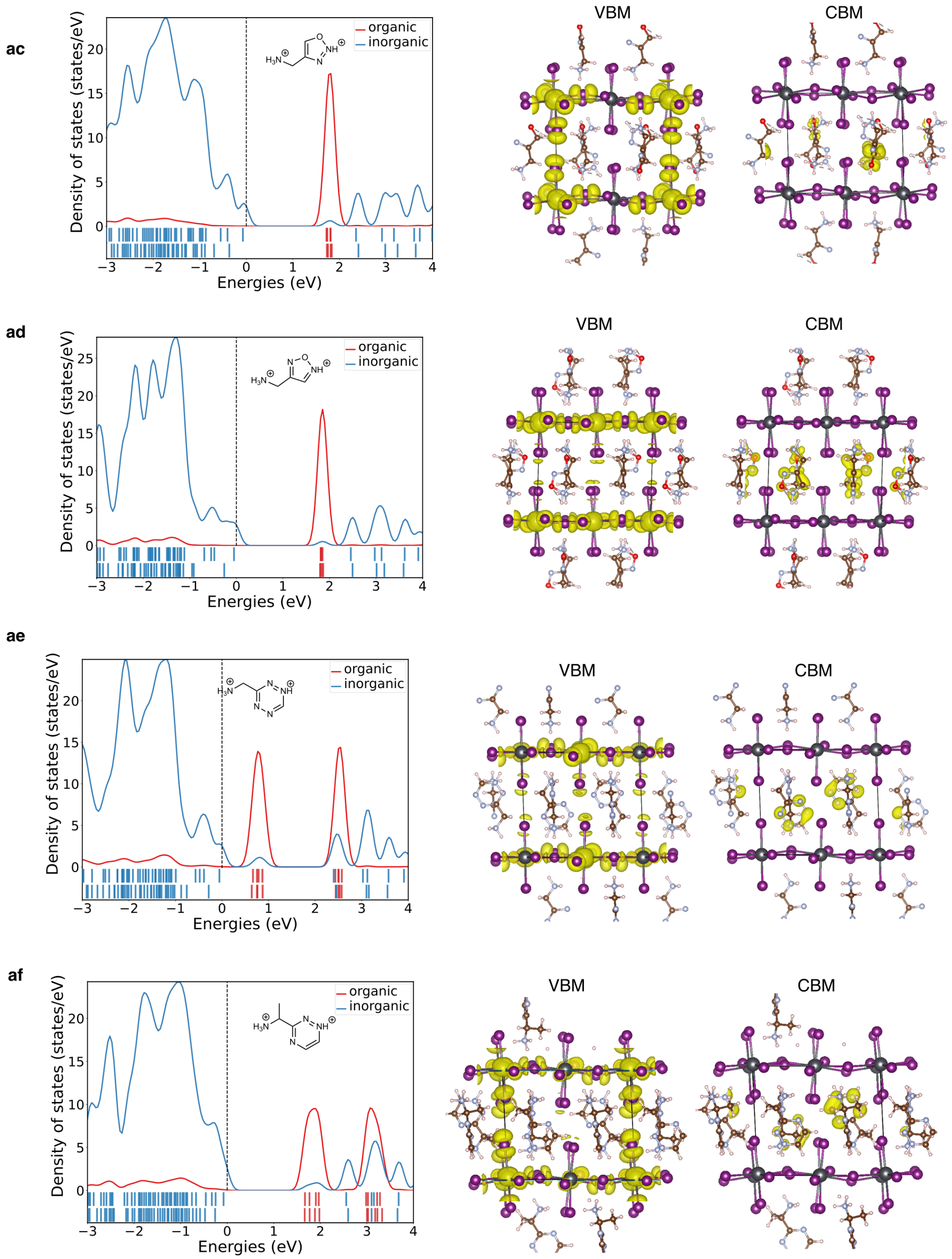

Figure S33: Electronic structure of inverse-designed type II_b DJ perovskites (Part 8, continued).

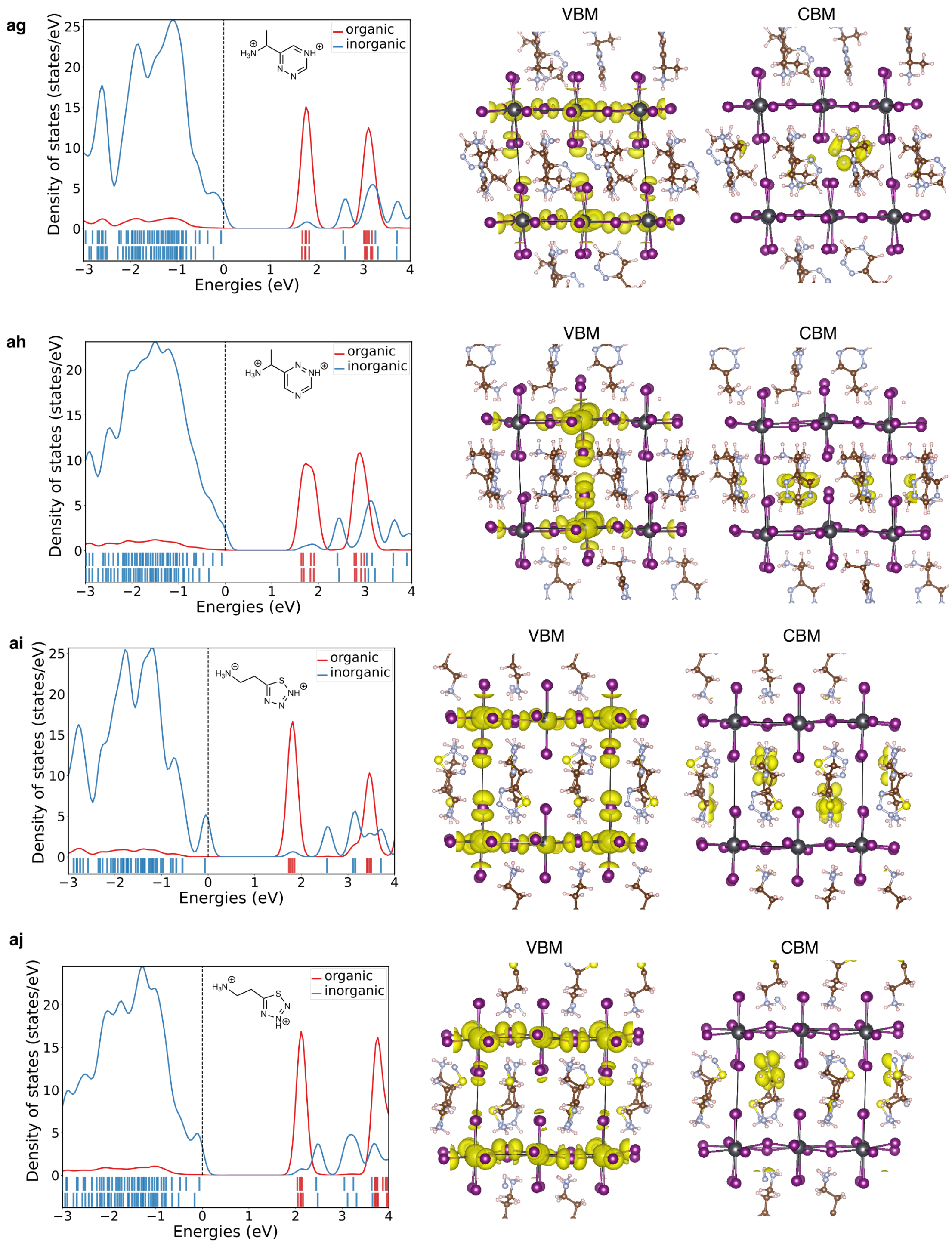

Figure S33: Electronic structure of inverse-designed type II_b DJ perovskites (Part 9, continued).

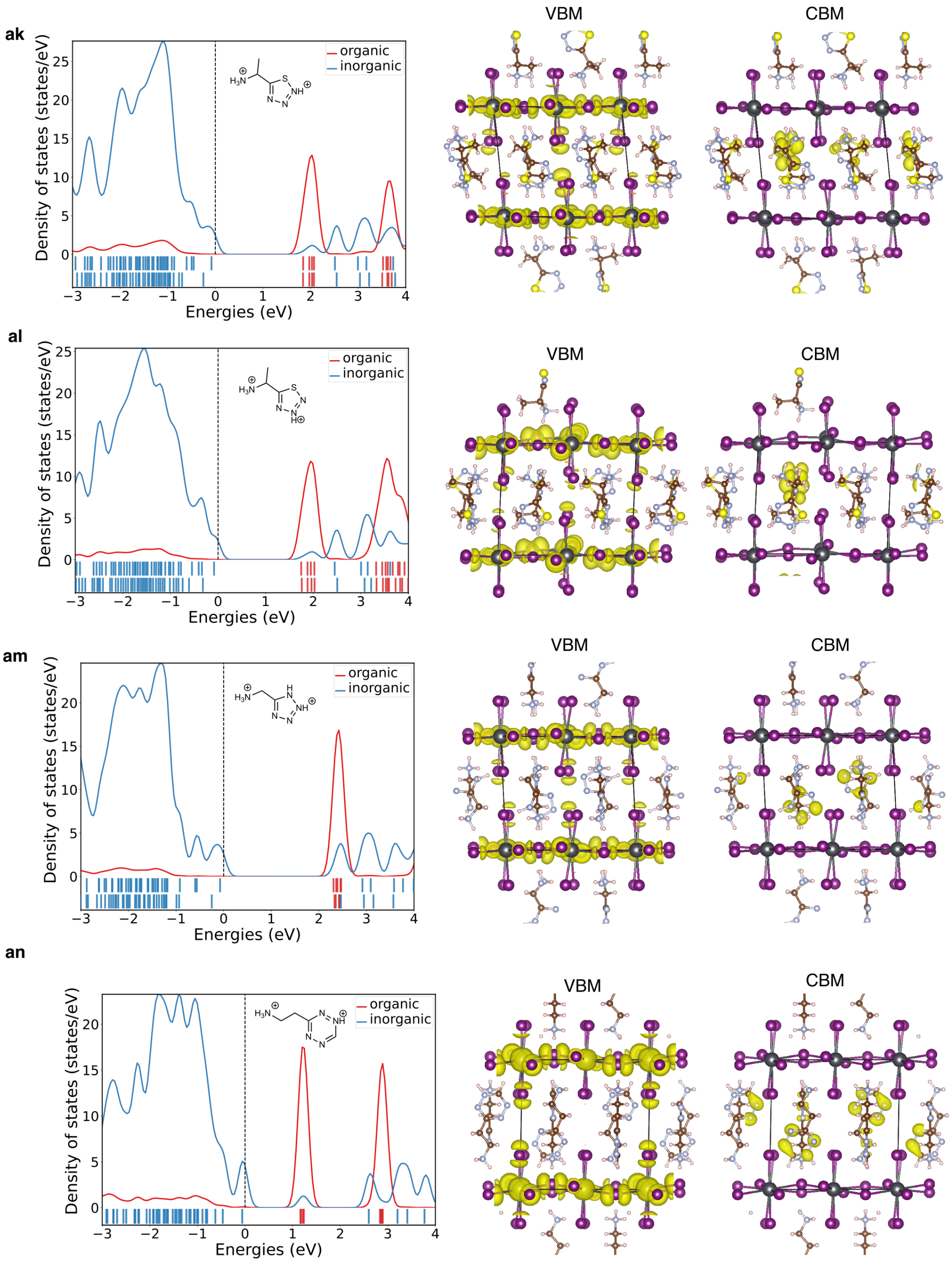

Figure S33: Electronic structure of inverse-designed type II_b DJ perovskites (Part 10, continued).

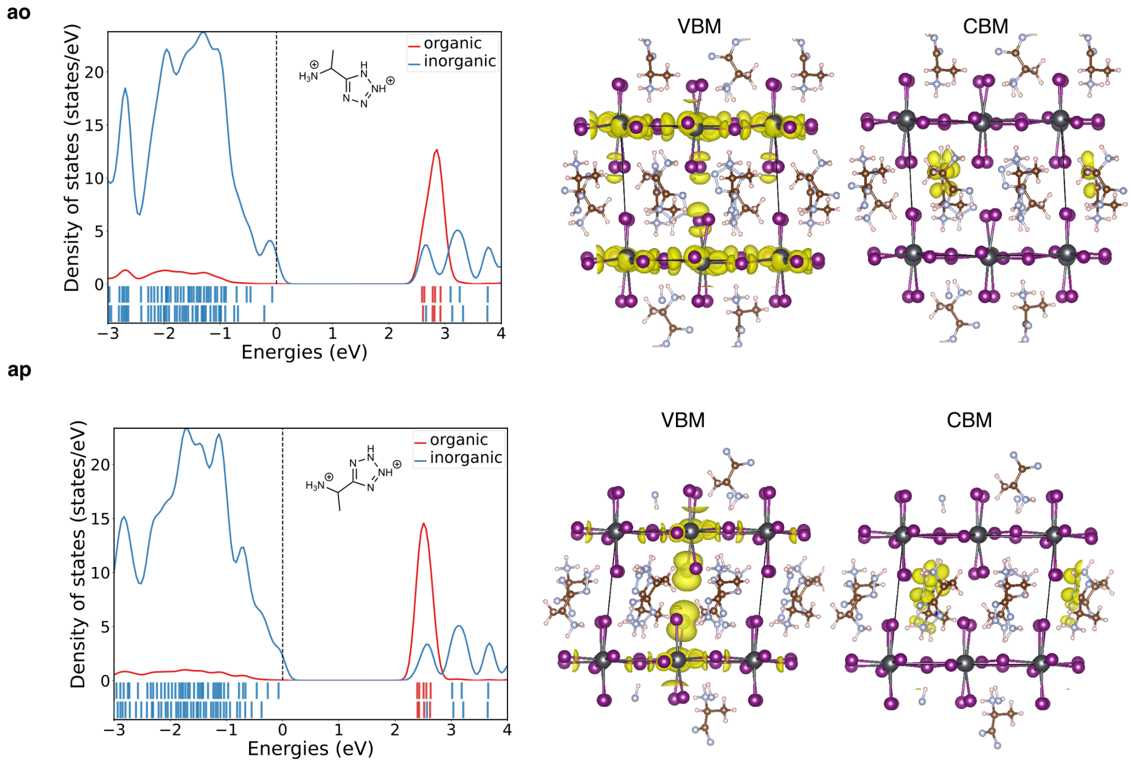

Figure S33: Electronic structure of inverse-designed type II_b DJ perovskites (Part 11, continued).

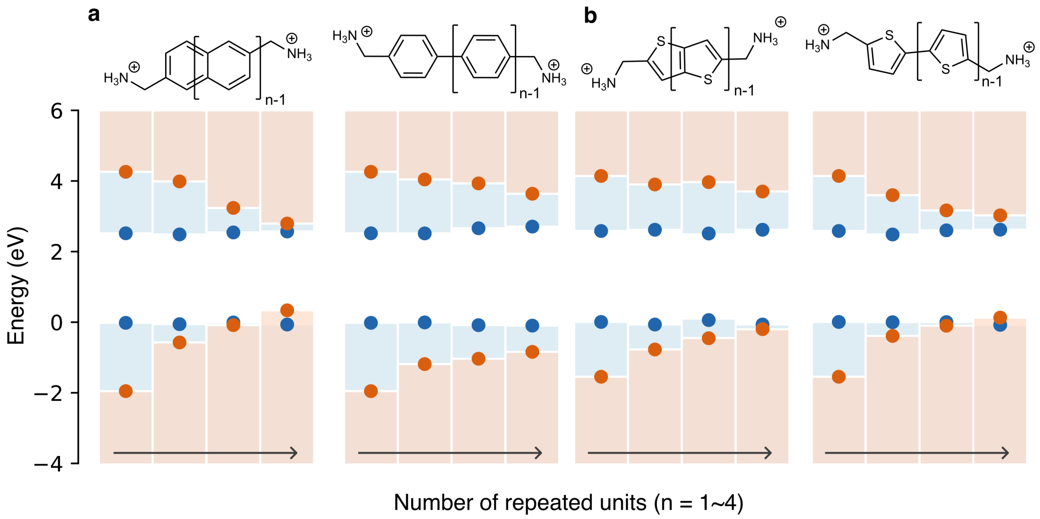

Figure S34: Reduction in HOMO-LUMO gap with increased π-conjugation, exemplified by **a** benzene-based and **b** thiophene-based spacers connected by fusion or linking.

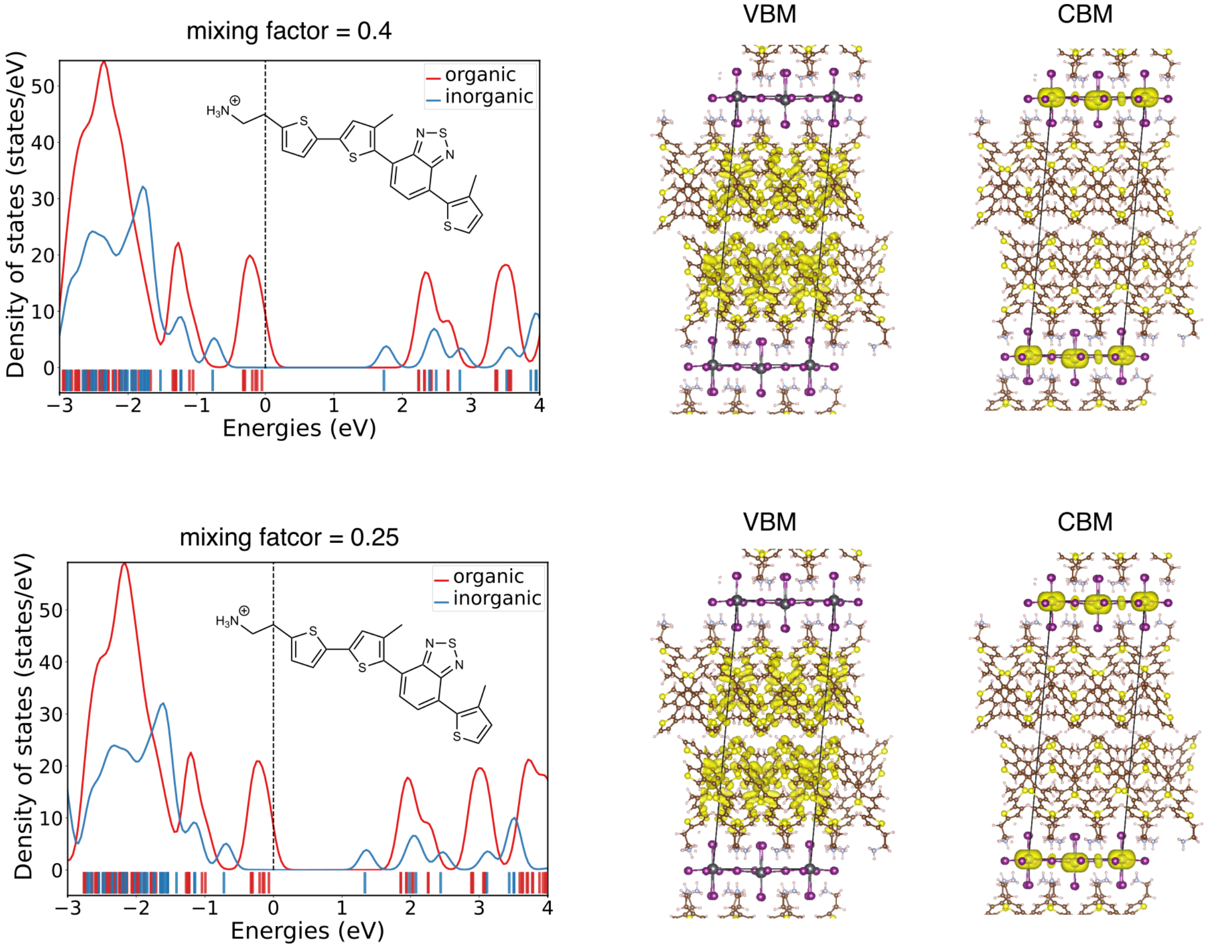

Figure S35: Energy level alignment of the previously reported RP-phase spacer (*33*) exhibiting type I_b alignment characteristic. Our calculations, performed using the same perovskite structure as in their study, result in a Type II_a alignment, regardless of whether we apply the systematic mixing factor of 0.4 used throughout our study or the 0.25 mixing factor employed in the previous work. The discrepancy in computational results is likely due to differences in software implementations and methodological choices. While direct comparisons across different simulation methodologies and experimental techniques remain challenging in 2D perovskite research (*49*), the relative trends within our calculations—conducted with a consistent set of computational parameters—are internally reliable.

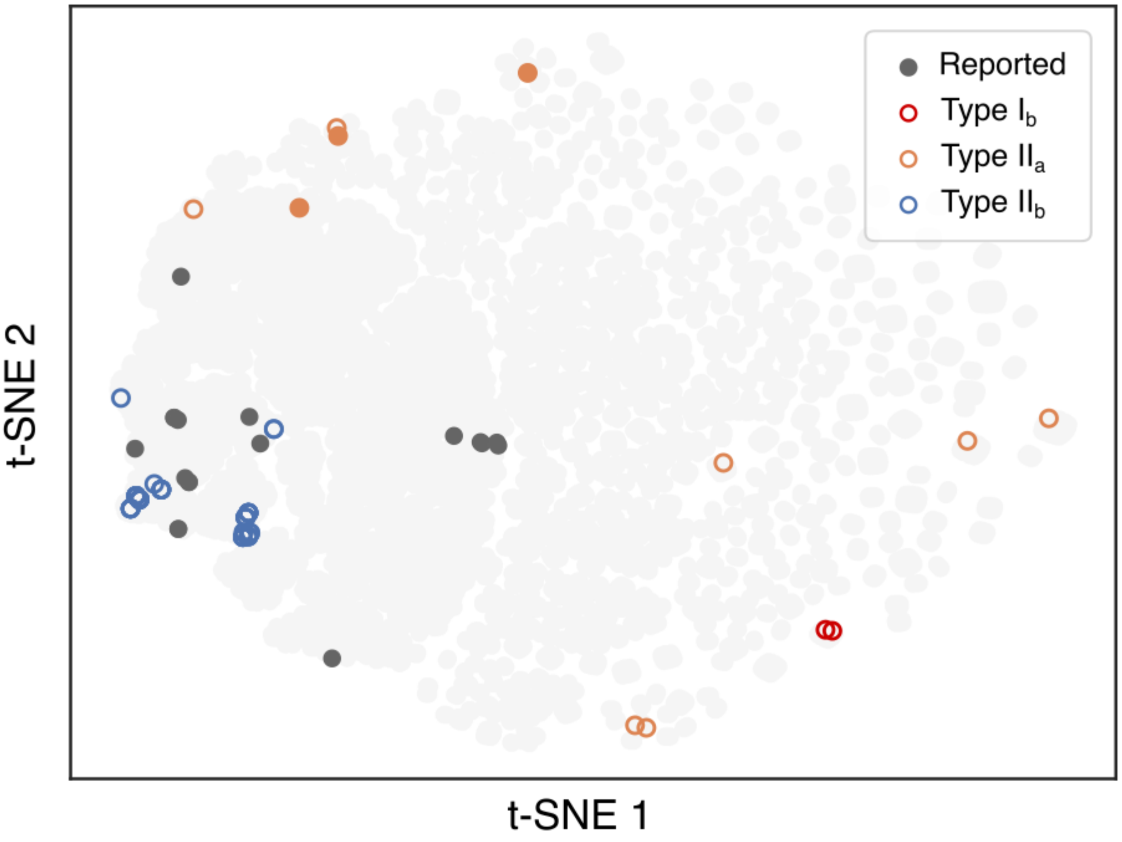

Figure S36: Chemical space visualization of inverse-designed DJ perovskites.

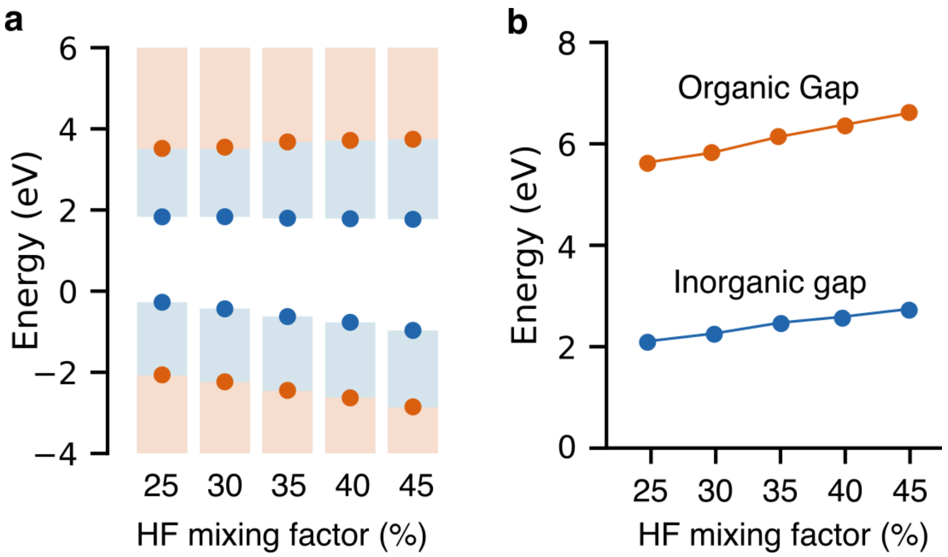

Figure S37: Effect of HF mixing factor on the calculated energy gap of organic and inorganic components in DJ perovskites. A range of mixing factors (25% to 45%) has been employed in earlier studies to match experimental bandgap values. In this work, we select a mixing factor of 40%, as it yields the smallest average error of 0.05 eV. The mixing factor impacts the organic levels slightly more than the inorganic levels, though both follow very similar trends. This suggests that the energy level alignment type is unlikely to vary significantly for most proposed DJ perovskites.

Table S1: Comparison of calculated bandgap values and reported experimental values.

**Compound**	**HSE06 + SOC**	**Experiment**
1	2.42	- (*54*)
2	2.26	2.34 (*54*)
3	2.39	2.42 (*74*)
4	2.56	2.44 (*75*), 2.42 (*47*)
5	2.42	2.46 (*75*), 2.43 (*76*)
6	2.57	2.58 (*76*)
7	2.38	2.52 (*62*)
8	2.56	2.42 (*62*)
9	2.56	2.39 (*62*)
10	2.79	- (*50*)
11	2.38	2.38 (*49*)

Table S2: Effect of HF mixing factor on the bandgap values of DJ perovskites.

**Compounds**	**Experiment**	**25%**	**30%**	**35%**	**40%**	**45%**
2	2.34 (*54*)	1.82	1.97	2.11	2.26	2.42
4	2.44 (*75*), 2.42 (*47*)	2.12	2.26	2.41	2.56	2.71

Table S3: Fingerprint criteria for targeted energy level alignment type.

	Type Ib	Type IIa		Type IIb
		oligothiophene-based	acene-based	
no. rings	[5, 7]	[2, 6]	[2, 4]	1
% ring linkage	0	1	0	0
% 6-membered rings	1	0	1	[0, 1]
no. primary ammonium	2	2	2	1
linker length	[0, 2]	[0, 6]	[0, 6]	[0, 3]
ammonium position	[0.5, 1]	1	1	(0, 1]
no. N (pyridine)	0	0	0	[0, 4]
no. F	0	0	0	[0, 2]
no. O (furan)	0	0	0	[0, 1]
no. N (pyrrole)	0	[0, 2]	0	[0, 1]
no. side chain (linker)	0	0	0	[0, 2]
no. side chain (backbone)	0	0	0	0
Number of molecules	2	11	1	42

Table S4: List of inverse-designed type I_b DJ perovskites.

	Molecular structure	Generation	Fingerprint	cid	Formability score	LogP
**a**	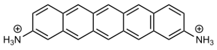	G7	[5,0,1,2,0,10/11,0,0,0,0,0,0]	85868421	0.99	5.46
**b**	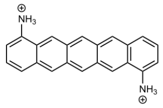	G8	[5,0,1,2,0,9/11,0,0,0,0,0,0]	154316963	0.99	5.46

Table S5: List of inverse-designed type II_a DJ perovskites. We used $LogP$ as a qualitative estimation for solubility. Some organic spacers, those above 6, may exhibit poor solubility, which could pose a challenge in synthesis. However, this also depends on the specific solutions chosen in synthesis.

	Molecular structure	Generation	Fingerprint	cid	Formability score	LogP
**a**	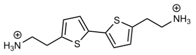	G4	[2,1,0,2,4,1,0,0,0,0,0,0]	170868983	1.00	2.48
**b**	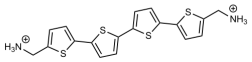	G4	[4,1,0,2,2,1,0,0,0,0,0,0]	392006	1.00	5.85
**c**	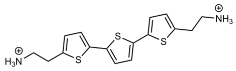	G5	[3,1,0,2,4,1,0,0,0,0,0,0]	85702348	1.00	4.21
**d**	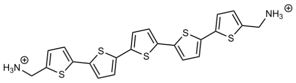	G5	[5,1,0,2,2,1,0,0,0,0,0,0]	10719177	1.00	7.58
**e**	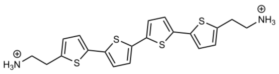	G6	[4,1,0,2,4,1,0,0,0,0,0,0]	10835744	1.00	5.94
**f**	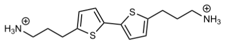	G6	[2,1,0,2,6,1,0,0,0,0,0,0]	85702352	1.00	3.26
**g**	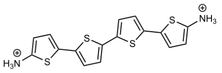	G6	[4,1,0,2,0,1,0,0,0,0,0,0]	142729566	0.99	6.10
**h**	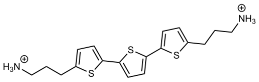	G7	[3,1,0,2,6,1,0,0,0,0,0,0]	85702353	1.00	4.99
**i**	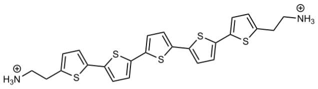	G7	[5,1,0,2,4,1,0,0,0,0,0,0]	85702350	1.00	7.66
**j**	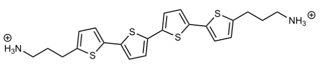	G8	[4,1,0,2,6,1,0,0,0,0,0,0]	85702356	1.00	6.72
**k**	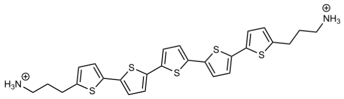	G9	[5,1,0,2,6,1,0,0,0,0,0,0]	85702358	1.00	8.44
**l**	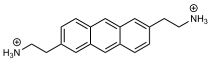	G4	[3,0,1,2,4,1,0,0,0,0,0,0]	138986756	1.00	3.00

Table S6: List of inverse-designed type II_b DJ perovskites. We use $LogP$ as a qualitative estimation for solubility of the organic spacer. Organic spacers with $LogP$ below -1 may post a challenge due to relatively high polarity. They tend to have poor solubility in nonpolar or less polar solvents.

Molecular structure	Generation	Fingerprint	Formability score	LogP
a, b 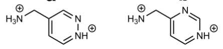	G2	[1,0,1,1,1,1,1,0,0,0,0,0]	0.98	-0.06
c, d, e, f, g 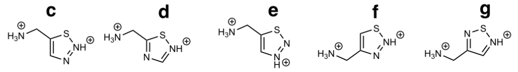	G3	[1,0,0,1,1,1,1,0,0,0,0,0]	0.97	0.00
h, i, j 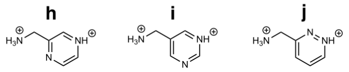	G3	[1,0,1,1,1,2/3,1,0,0,0,0,0]	0.96	-0.06
k, l, m, n 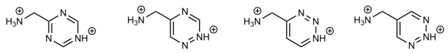	G3	[1,0,1,1,1,1,2,0,0,0,0,0]	0.98	-0.67
o 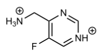	G3	[1,0,1,1,1,1,1,1,0,0,0,0]	0.97	0.07
p, q, r 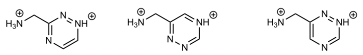	G4	[1,0,1,1,1,2/3,2,0,0,0,0,0]	0.96	-0.67
s, t 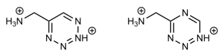	G4	[1,0,1,1,1,1,3,0,0,0,0,0]	0.98	-1.27
u, v, w 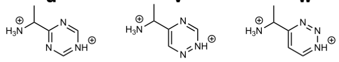	G4	[1,0,1,1,1,1,2,0,0,0,1,0]	0.98	-0.11
x, y 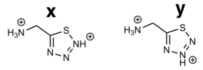	G4	[1,0,0,1,1,1,2,0,0,0,0,0]	0.97	-0.61
z, aa, ab, ac, ad 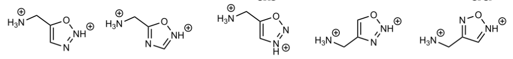	G4	[1,0,0,1,1,1,1,0,1,0,0,0]	0.97	-0.47
ae 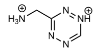	G5	[1,0,1,1,1,2/3,3,0,0,0,0,0]	0.96	-1.27
af, ag, ah 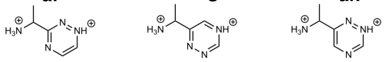	G5	[1,0,1,1,1,2/3,2,0,0,0,1,0]	0.95	-0.11
ai, aj 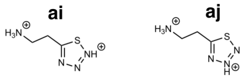	G5	[1,0,0,1,2,1,2,0,0,0,0,0]	1.00	-0.57
ak, al 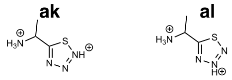	G5	[1,0,0,1,1,1,2,0,0,0,1,0]	0.96	-0.05
am 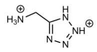	G5	[1,0,0,1,1,1,2,0,0,1,0,0]	0.97	-1.34
an 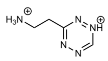	G6	[1,0,1,1,2,2/3,3,0,0,0,0,0]	0.99	-1.23
ao, ap 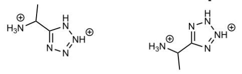	G6	[1,0,0,1,1,1,2,0,0,1,1,0]	0.96	-0.78

Table S7: Properties of selected inverse-designed final candidates for type I_b, II_a, and II_b energy level alignment.

Energy level alignment	Molecular structure	Fingerprint	Generation	cid	Formability score
Type Ib	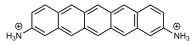	[5,0,1,2,0,10/11,0,0,0,0,0,0]	G7	85868421	0.99
	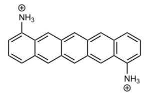	[5,0,1,2,0,9/11,0,0,0,0,0,0]	G8	154316963	0.99
Type IIa	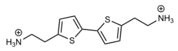	[2,1,0,2,4,1,0,0,0,0,0,0]	G4	170868983	1.00
	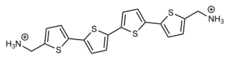	[4,1,0,2,2,1,0,0,0,0,0,0]	G4	392006	1.00
	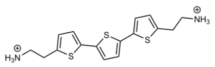	[3,1,0,2,4,1,0,0,0,0,0,0]	G5	85702348	1.00
	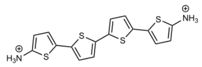	[4,1,0,2,0,1,0,0,0,0,0,0]	G6	142729566	0.99
	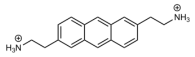	[3,0,1,2,4,1,0,0,0,0,0,0]	G4	138986756	1.00
Type IIb	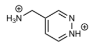	[1,0,1,1,1,1,1,0,0,0,0,0]	G2	15034997	0.98
	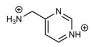	[1,0,1,1,1,1,1,0,0,0,0,0]	G2	12933348	0.98
	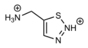	[1,0,0,1,1,1,1,0,0,0,0,0]	G3	54534495	0.97
	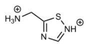	[1,0,0,1,1,1,1,0,0,0,0,0]	G3	82418955	0.97
	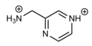	[1,0,1,1,1,2/3,1,0,0,0,0,0]	G3	266781	0.96
	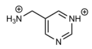	[1,0,1,1,1,2/3,1,0,0,0,0,0]	G3	18953632	0.96

REFERENCES

1. B. A. Koscher, R. B. Canty, M. A. McDonald, K. P. Greenman, C. J. McGill, C. L. Bilodeau, W. Jin, H. Wu, F. H. Vermeire, B. Jin, T. Hart, T. Kulesza, S. C. Li, T. S. Jaakkola, R. Barzilay, R. Gomez-Bombarelli, W. H. Green, K. F. Jensen, Autonomous, multiproperty-driven molecular discovery: From predictions to measurements and back. *Science* **382**, eadi1407 (2023).

2. P. Raccuglia, K. C. Elbert, P. D. Adler, C. Falk, M. B. Wenny, A. Mollo, M. Zeller, S. A. Friedler, J. Schrier, A. J. Norquist, Machine-learning-assisted materials discovery using failed experiments. *Nature* **533**, 73–76 (2016).

3. J. Peng, D. Schwalbe-Koda, K. Akkiraju, T. Xie, L. Giordano, Y. Yu, C. J. Eom, J. R. Lunger, D. J. Zheng, R. R. Rao, S. Muy, J. C. Grossman, K. Reuter, R. Gómez-Bombarelli, Y. Shao-Horn, Human- and machine-centred designs of molecules and materials for sustainability and decarbonization. *Nat. Rev. Mater.* **7**, 991–1009 (2022).

4. J. Xu, H. Chen, L. Grater, C. Liu, Y. Yang, S. Teale, A. Maxwell, S. Mahesh, H. Wan, Y. Chang, B. Chen, B. Rehl, S. M. Park, M. G. Kanatzidis, E. H. Sargent, Anion optimization for bifunctional surface passivation in perovskite solar cells. *Nat. Mater.* **22**, 1507–1514 (2023).

5. F. Strieth-Kalthoff, H. Hao, V. Rathore, J. Derasp, T. Gaudin, N. H. Angello, M. Seifrid, E. Trushina, M. Guy, J. Liu, X. Tang, M. Mamada, W. Wang, T. Tsagaantsooj, C. Lavigne, R. Pollice, T. C. Wu, K. Hotta, L. Bodo, S. Li, M. Haddadnia, A. Wołos, R. Roszak, C. T. Ser, C. Bozal-Ginesta, R. J. Hickman, J. Vestfrid, A. Aguilar-Granda, E. L. Klimareva, R. C. Sigerson, W. Hou, D. Gahler, S. Lach, A. Warzybok, O. Borodin, S. Rohrbach, B. Sanchez-Lengeling, C. Adachi, B. A. Grzybowski, L. Cronin, J. E. Hein, M. D. Burke, A. Aspuru-Guzik, Delocalized, asynchronous, closed-loop discovery of organic laser emitters. *Science* **384**, eadk9227 (2024).

6. Z. Rao, P.-Y. Tung, R. Xie, Y. Wei, H. Zhang, A. Ferrari, T. P. C. Klaver, F. Körmann, P. T. Sukumar, A. Kwiatkowski da Silva, Y. Chen, Z. Li, D. Ponge, J. Neugebauer, O. Gutfleisch, S. Bauer, D. Raabe, Machine learning–enabled high-entropy alloy discovery. *Science* **378**, 78–85 (2022).

7. A. Merchant, S. Batzner, S. S. Schoenholz, M. Aykol, G. Cheon, E. D. Cubuk, Scaling deep learning for materials discovery. *Nature* **624**, 80–85 (2023).

8. A. Zunger, Inverse design in search of materials with target functionalities. *Nat. Rev. Chem.* **2**, 0121 (2018).

9. B. Sanchez-Lengeling, A. Aspuru-Guzik, Inverse molecular design using machine learning: Generative models for matter engineering. *Science* **361**, 360–365 (2018).

10. Z. Yao, B. Sánchez-Lengeling, N. S. Bobbitt, B. J. Bucior, S. G. H. Kumar, S. P. Collins, T. Burns, T. K. Woo, O. K. Farha, R. Q. Snurr, A. Aspuru-Guzik, Inverse design of nanoporous crystalline reticular materials with deep generative models. *Nat. Mach. Intell.* **3**, 76–86 (2021).

11. B. Kim, S. Lee, J. Kim, Inverse design of porous materials using artificial neural networks. *Sci. Adv.* **6**, eaax9324 (2020).

12. J. Wu, L. Torresi, M. Hu, P. Reiser, J. Zhang, J. S. Rocha-Ortiz, L. Wang, Z. Xie, K. Zhang, B.-w. Park, A. Barabash, Y. Zhao, J. Luo, Y. Wang, L. Lüer, L.-L. Deng, J. A. Hauch, D. M. Guldi, M. E. Pérez-Ojeda, S. I. Seok, P. Friederich, C. J. Brabec, Inverse design workflow discovers hole-transport materials tailored for perovskite solar cells. *Science* **386**, 1256–1264 (2024).

13. Z. Ren, S. I. P. Tian, J. Noh, F. Oviedo, G. Xing, J. Li, Q. Liang, R. Zhu, A. G. Aberle, S. Sun, X. Wang, Y. Liu, Q. Li, S. Jayavelu, K. Hippalgaonkar, Y. Jung, T. Buonassisi, An invertible crystallographic representation for general inverse design of inorganic crystals with targeted properties. *Matter* **5**, 314–335 (2022).

14. X. Li, J. M. Hoffman, M. G. Kanatzidis, The 2D halide perovskite rulebook: How the spacer influences everything from the structure to optoelectronic device efficiency. *Chem. Rev.* **121**, 2230–2291 (2021).

15. E. Shi, Y. Gao, B. P. Finkenauer, A. H. Akriti, L. D. Coffey, Two-dimensional halide perovskite nanomaterials and heterostructures. *Chem. Soc. Rev.* **47**, 6046–6072 (2018).

16. J. Sun, K. Wang, K. Ma, J. Y. Park, Z. Y. Lin, B. M. Savoie, L. Dou, Emerging two-dimensional organic semiconductor-incorporated perovskites horizontal line—A fascinating family of hybrid electronic materials. *J. Am. Chem. Soc.* **145**, 20694–20715 (2023).

17. F. Zhang, S. Y. Park, C. Yao, H. Lu, S. P. Dunfield, C. Xiao, S. Ulicna, X. Zhao, L. Du Hill, X. Chen, X. Wang, L. E. Mundt, K. H. Stone, L. T. Schelhas, G. Teeter, S. Parkin, E. L. Ratcliff, Y. L. Loo, J. J. Berry, M. C. Beard, Y. Yan, B. W. Larson, K. Zhu, Metastable Dion-Jacobson 2D structure enables efficient and stable perovskite solar cells. *Science* **375**, 71–76 (2022).

18. S. Sidhik, Y. Wang, M. De Siena, R. Asadpour, A. J. Torma, T. Terlier, K. Ho, W. Li, A. B. Puthirath, X. Shuai, A. Agrawal, B. Traore, M. Jones, R. Giridharagopal, P. M. Ajayan, J. Strzalka, D. S. Ginger, C. Katan, M. A. Alam, J. Even, M. G. Kanatzidis, A. D. Mohite, Deterministic fabrication of 3D/2D perovskite bilayer stacks for durable and efficient solar cells. *Science* **377**, 1425–1430 (2022).

19. Y. Li, K. Yang, High-throughput computational design of halide perovskites and beyond for optoelectronics. *WIREs Comput. Mol. Sci.* **11**, e1500 (2020).

20. Y. Shang, Y. Liao, Q. Wei, Z. Wang, B. Xiang, Y. Ke, W. Liu, Z. Ning, Highly stable hybrid perovskite light-emitting diodes based on Dion-Jacobson structure. *Sci. Adv.* **5**, eaaw8072 (2019).

21. Y. Dong, X. Dong, D. Lu, M. Chen, N. Zheng, R. Wang, Q. Li, Z. Xie, Y. Liu, Orbital interactions between the organic semiconductor spacer and the inorganic layer in Dion-Jacobson perovskites enable efficient solar cells. *Adv. Mater.* **35**, e2205258 (2023).

22. Z. Xu, D. Lu, X. Dong, M. Chen, Q. Fu, Y. Liu, Highly efficient and stable Dion-Jacobson perovskite solar cells enabled by extended pi-conjugation of organic spacer. *Adv. Mater.* **33**, e2105083 (2021).

23. W. Li, S. Sidhik, B. Traore, R. Asadpour, J. Hou, H. Zhang, A. Fehr, J. Essman, Y. Wang, J. M. Hoffman, I. Spanopoulos, J. J. Crochet, E. Tsai, J. Strzalka, C. Katan, M. A. Alam, M. G. Kanatzidis, J. Even, J. C. Blancon, A. D. Mohite, Light-activated interlayer contraction in

two-dimensional perovskites for high-efficiency solar cells. *Nat. Nanotechnol.* **17**, 45–52 (2022).

24. F. Zhang, D. H. Kim, H. Lu, J. S. Park, B. W. Larson, J. Hu, L. Gao, C. Xiao, O. G. Reid, X. Chen, Q. Zhao, P. F. Ndione, J. J. Berry, W. You, A. Walsh, M. C. Beard, K. Zhu, Enhanced charge transport in 2D perovskites via fluorination of organic cation. *J. Am. Chem. Soc.* **141**, 5972–5979 (2019).

25. S. Guo, Y. Li, Y. Mao, W. Tao, K. Bu, T. Fu, C. Zhao, H. Luo, Q. Hu, H. Zhu, E. Shi, W. Yang, L. Dou, X. Lü, Reconfiguring band-edge states and charge distribution of organic semiconductor–incorporated 2D perovskites via pressure gating. *Sci. Adv.* **8**, eadd1984 (2022).

26. J. Hu, I. W. H. Oswald, S. J. Stuard, M. M. Nahid, N. Zhou, O. F. Williams, Z. Guo, L. Yan, H. Hu, Z. Chen, X. Xiao, Y. Lin, Z. Yang, J. Huang, A. M. Moran, H. Ade, J. R. Neilson, W. You, Synthetic control over orientational degeneracy of spacer cations enhances solar cell efficiency in two-dimensional perovskites. *Nat. Commun.* **10**, 1276 (2019).

27. Y. Liu, H. Zhou, Y. Ni, J. Guo, R. Lu, C. Li, X. Guo, Revealing stability origin of Dion-Jacobson 2D perovskites with different-rigidity organic cations. *Joule* **7**, 1016–1032 (2023).

28. J. Y. Park, R. Song, J. Liang, L. Jin, K. Wang, S. Li, E. Shi, Y. Gao, M. Zeller, S. J. Teat, P. Guo, L. Huang, Y. S. Zhao, V. Blum, L. Dou, Thickness control of organic semiconductor-incorporated perovskites. *Nat. Chem.* **15**, 1745–1753 (2023).

29. E. Shi, B. Yuan, S. B. Shiring, Y. Gao, Y. Akriti, C. Guo, M. Su, P. Lai, J. Yang, B. M. Kong, Y. Savoie, L. D. Yu, Two-dimensional halide perovskite lateral epitaxial heterostructures. *Nature* **580**, 614–620 (2020).

30. J. W. Lee, S. Tan, S. I. Seok, Y. Yang, N. G. Park, Rethinking the A cation in halide perovskites. *Science* **375**, eabj1186 (2022).

31. C. Liu, W. Huhn, K. Z. Du, A. Vazquez-Mayagoitia, D. Dirkes, W. You, Y. Kanai, D. B. Mitzi, V. Blum, Tunable semiconductors: Control over carrier states and excitations in layered hybrid organic-inorganic perovskites. *Phys. Rev. Lett.* **121**, 146401 (2018).

32. Q. Dai, H. Li, G. Sini, J. L. Bredas, Evolution of the nature of excitons and electronic couplings in hybrid 2D perovskites as a function of organic cation π-conjugation. *Adv. Funct. Mater.* **32**, 2108662 (2021).

33. Y. Gao, E. Shi, S. Deng, S. B. Shiring, J. M. Snaider, C. Liang, B. Yuan, R. Song, S. M. Janke, A. Liebman-Pelaez, P. Yoo, M. Zeller, B. W. Boudouris, P. Liao, C. Zhu, V. Blum, Y. Yu, B. M. Savoie, L. Huang, L. Dou, Molecular engineering of organic-inorganic hybrid perovskites quantum wells. *Nat. Chem.* **11**, 1151–1157 (2019).

34. K. Wang, Z. Y. Lin, A. De, C. A. Kocoj, W. Shao, H. Yang, Z. He, A. H. Coffey, C. B. Fruhling, Y. Tang, D. Varadharajan, C. Zhu, Y. S. Zhao, A. Boltasseva, V. M. Shalaev, P. Guo, B. M. Savoie, L. Dou, Two-dimensional-lattice-confined single-molecule-like aggregates. *Nature* **633**, 567–574 (2024).

35. R. Lyu, C. E. Moore, T. Liu, Y. Yu, Y. Wu, Predictive design model for low-dimensional organic-inorganic halide perovskites assisted by machine learning. *J. Am. Chem. Soc.* **143**, 12766–12776 (2021).

36. Y. Wu, C. F. Wang, M. G. Ju, Q. Jia, Q. Zhou, S. Lu, X. Gao, Y. Zhang, J. Wang, Universal machine learning aided synthesis approach of two-dimensional perovskites in a typical laboratory. *Nat. Commun.* **15**, 138 (2024).

37. Z. Y. Lin, J. Sun, S. B. Shiring, L. Dou, B. M. Savoie, Design rules for two-dimensional organic semiconductor-incorporated perovskites (OSiP) gleaned from thousands of simulated structures. *Angew. Chem. Int. Ed. Engl.* **62**, e202305298 (2023).

38. Y. Wu, S. Lu, Q. Zhou, M. G. Ju, X. C. Zeng, J. Wang, Two-dimensional perovskites with tunable room-temperature phosphorescence. *Adv. Funct. Mater.* **32**, 2204579 (2022).

39. M. Bhatt, P. K. Nayak, D. Ghosh, Data-driven design of electroactive spacer molecules to tune charge carrier dynamics in layered halide perovskite heterostructures. *ACS Nano* **18**, 24484–24494 (2024).

40. E. Mahal, D. Roy, S. S. Manna, B. Pathak, Machine learning-driven prediction of band-alignment types in 2D hybrid perovskites. *J. Mater. Chem. A* **11**, 23547–23555 (2023).

41. J. Moon, W. Beker, M. Siek, J. Kim, H. S. Lee, T. Hyeon, B. A. Grzybowski, Active learning guides discovery of a champion four-metal perovskite oxide for oxygen evolution electrocatalysis. *Nat. Mater.* **23**, 108–115 (2024).

42. Y. Li, K. Yang, High-throughput computational design of organic–inorganic hybrid halide semiconductors beyond perovskites for optoelectronics. *Energ. Environ. Sci.* **12**, 2233–2243 (2019).

43. A. M. Virshup, J. Contreras-Garcia, P. Wipf, W. Yang, D. N. Beratan, Stochastic voyages into uncharted chemical space produce a representative library of all possible drug-like compounds. *J. Am. Chem. Soc.* **135**, 7296–7303 (2013).

44. H. Li, H. Zheng, T. Yue, Z. Xie, S. Yu, J. Zhou, T. Kapri, Y. Wang, Z. Cao, H. Zhao, A. Kemelbay, J. He, G. Zhang, P. F. Pieters, E. A. Dailing, J. R. Cappiello, M. Salmeron, X. Gu, T. Xu, P. Wu, Y. Li, K. B. Sharpless, Y. Liu, Machine learning-accelerated discovery of heat-resistant polysulfates for electrostatic energy storage. *Nat. Energy* **10**, 90–100 (2025).

45. X. Li, Y. Che, L. Chen, T. Liu, K. Wang, L. Liu, H. Yang, E. O. Pyzer-Knapp, A. I. Cooper, Sequential closed-loop Bayesian optimization as a guide for organic molecular metallophotocatalyst formulation discovery. *Nat. Chem.* **16**, 1286–1294 (2024).

46. C. Kunkel, J. T. Margraf, K. Chen, H. Oberhofer, K. Reuter, Active discovery of organic semiconductors. *Nat. Commun.* **12**, 2422 (2021).

47. Y. Li, J. V. Milic, A. Ummadisingu, J. Y. Seo, J. H. Im, H. S. Kim, Y. Liu, M. I. Dar, S. M. Zakeeruddin, P. Wang, A. Hagfeldt, M. Gratzel, Bifunctional organic spacers for

formamidinium-based hybrid Dion-Jacobson two-dimensional perovskite solar cells. *Nano Lett.* **19**, 150–157 (2019).

48. M. Zhong, K. Tran, Y. Min, C. Wang, Z. Wang, C. T. Dinh, P. De Luna, Z. Yu, A. S. Rasouli, P. Brodersen, S. Sun, O. Voznyy, C. S. Tan, M. Askerka, F. Che, M. Liu, A. Seifitokaldani, Y. Pang, S. C. Lo, A. Ip, Z. Ulissi, E. H. Sargent, Accelerated discovery of CO_2 electrocatalysts using active machine learning. *Nature* **581**, 178–183 (2020).

49. W. A. Dunlap-Shohl, E. T. Barraza, A. Barrette, S. Dovletgeldi, G. Findik, D. J. Dirkes, C. Liu, M. K. Jana, V. Blum, W. You, K. Gundogdu, A. D. Stiff-Roberts, D. B. Mitzi, Tunable internal quantum well alignment in rationally designed oligomer-based perovskite films deposited by resonant infrared matrix-assisted pulsed laser evaporation. *Mater. Horiz.* **6**, 1707–1716 (2019).

50. M. K. Jana, C. Liu, S. Lidin, D. J. Dirkes, W. You, V. Blum, D. B. Mitzi, Resolving rotational stacking disorder and electronic level alignment in a 2D oligothiophene-based lead iodide perovskite. *Chem. Mater.* **31**, 8523–8532 (2019).

51. J. P. Perdew, K. Burke, M. Ernzerhof, Generalized gradient approximation made simple. *Phys. Rev. Lett.* **77**, 3865–3868 (1996).

52. A. Jain, S. P. Ong, G. Hautier, W. Chen, W. D. Richards, S. Dacek, S. Cholia, D. Gunter, D. Skinner, G. Ceder, K. A. Persson, Commentary: The materials project: A materials genome approach to accelerating materials innovation. *APL Mater.* **1**, 011002 (2013).

53. J. Heyd, G. E. Scuseria, M. Ernzerhof, Hybrid functionals based on a screened Coulomb potential. *J. Chem. Phys.* **118**, 8207–8215 (2003).

54. X. Li, W. Ke, B. Traore, P. Guo, I. Hadar, M. Kepenekian, J. Even, C. Katan, C. C. Stoumpos, R. D. Schaller, M. G. Kanatzidis, Two-dimensional Dion-Jacobson hybrid lead iodide perovskites with aromatic diammonium cations. *J. Am. Chem. Soc.* **141**, 12880–12890 (2019).

55. L. Mao, E. E. Morgan, A. Li, R. M. Kennard, M. J. Hong, Y. Liu, C. J. Dahlman, J. G. Labram, M. L. Chabinyc, R. Seshadri, Layered hybrid lead iodide perovskites with short interlayer distances. *ACS Energy Lett.* **7**, 2801–2806 (2022).

56. X. Ni, H. Li, J.-L. Brédas, Enhanced organic-inorganic electronic coupling in two-dimensional hybrid perovskites through molecular engineering of dipolar pyrene-based cations. *ACS Mater. Lett.* **6**, 3436–3442 (2024).

57. Y. Boeije, F. Lie, M. Dubajic, E. Garip, A. Maufort, R. I. Biega, S. Lenaers, M. Sauty, P. Ghosh, A. Radic, A. Loher, P. La Magna, H. Salway, A. Ashoka, X. W. Chua, Q. Gu, K. Van Hecke, L. Lutsen, D. Vanderzande, A. Rao, W. T. M. Van Gompel, L. Leppert, S. D. Stranks, Molecular engineering of interlayer exciton delocalization in 2D perovskites. *J. Am. Chem. Soc.* **147**, 31541–31557 (2025).

58. C. Lee, W. Yang, R. G. Parr, Development of the Colle-Salvetti correlation-energy formula into a functional of the electron density. *Phys. Rev. B* **37**, 785–789 (1988).

59. K. T. Butler, D. W. Davies, H. Cartwright, O. Isayev, A. Walsh, Machine learning for molecular and materials science. *Nature* **559**, 547–555 (2018).

60. H. Bronstein, C. B. Nielsen, B. C. Schroeder, I. McCulloch, The role of chemical design in the performance of organic semiconductors. *Nat. Rev. Chem.* **4**, 66–77 (2020).

61. H. Zhang, R. Wang, L. Yang, Z. Hu, H. Liu, Y. Liu, Modulating the dipole moment of secondary ammonium spacers for efficient 2D Ruddlesden-Popper perovskite solar cells. *Angew. Chem. Int. Ed. Engl.* **63**, e202318206 (2024).

62. R. Zhao, R. P. Sabatini, T. Zhu, S. Wang, A. Morteza Najjarian, A. Johnston, A. J. Lough, S. Hoogland, E. H. Sargent, D. S. Seferos, Rigid conjugated diamine templates for stable Dion-Jacobson-type two-dimensional perovskites. *J. Am. Chem. Soc.* **143**, 19901–19908 (2021).

63. J. Jang, G. H. Gu, J. Noh, J. Kim, Y. Jung, Structure-based synthesizability prediction of crystals using partially supervised learning. *J. Am. Chem. Soc.* **142**, 18836–18843 (2020).

64. Z. Fang, X. Hou, Y. Zheng, Z. Yang, K. C. Chou, G. Shao, M. Shang, W. Yang, T. Wu, First-principles optimization of out-of-plane charge transport in Dion-Jacobson $CsPbI_3$ perovskites with π-conjugated aromatic spacers. *Adv. Funct. Mater.* **31**, 2102330 (2021).

65. G. Skoraczynski, M. Kitlas, B. Miasojedow, A. Gambin, Critical assessment of synthetic accessibility scores in computer-assisted synthesis planning. *J. Chem.* **15**, 6 (2023).

66. Y. Liu, J. Guo, H. Zhou, C. Li, X. Guo, Correlating pi-pi stacking of aromatic diammoniums with stability and dimensional reduction of Dion-Jacobson 2D perovskites. *J. Am. Chem. Soc.* **146**, 8198–8205 (2024).

67. Z. Luo, T. Xu, C. Zhang, C. Yang, Side-chain engineering of nonfullerene small-molecule acceptors for organic solar cells. *Energ. Environ. Sci.* **16**, 2732–2758 (2023).

68. H. Jiang, W. Hu, The emergence of organic single-crystal electronics. *Angew. Chem. Int. Ed. Engl.* **59**, 1408–1428 (2020).

69. D. Weininger, SMILES, a chemical language and information system. 1. Introduction to methodology and encoding rules. *J. Chem. Inf. Comput. Sci.* **28**, 31–36 (1988).

70. G. Kresse, J. Furthmüller, Efficient iterative schemes for ab initio total-energy calculations using a plane-wave basis set. *Phys. Rev. B* **54**, 11169–11186 (1996).

71. F. Pedregosa, G. Varoquaux, A. Gramfort, V. Michel, B. Thirion, O. Grisel, M. Blondel, P. Prettenhofer, R. Weiss, V. Dubourg, J. Vanderplas, A. Passos, D. Cournapeau, M. Brucher, M. Perrot, É. Duchesnay, Scikit-learn: Machine learning in Python. *J. Mach. Learn. Res.* **12**, 2825–2830 (2011).

72. B. Traore, L. Pedesseau, L. Assam, X. Che, J. C. Blancon, H. Tsai, W. Nie, C. C. Stoumpos, M. G. Kanatzidis, S. Tretiak, A. D. Mohite, J. Even, M. Kepenekian, C. Katan, Composite

nature of layered hybrid perovskites: Assessment on quantum and dielectric confinements and band alignment. *ACS Nano* **12**, 3321–3332 (2018).

73. B. Milian Medina, D. Beljonne, H. J. Egelhaaf, J. Gierschner, Effect of fluorination on the electronic structure and optical excitations of pi-conjugated molecules. *J. Chem. Phys.* **126**, 111101 (2007).

74. L. Gao, X. Li, B. Traore, Y. Zhang, J. Fang, Y. Han, J. Even, C. Katan, K. Zhao, S. Liu, M. G. Kanatzidis, *m*-Phenylenediammonium as a new spacer for Dion-Jacobson two-dimensional perovskites. *J. Am. Chem. Soc.* **143**, 12063–12073 (2021).

75. A. Ducinskas, G. C. Fish, M. A. Hope, L. Merten, D. Moia, A. Hinderhofer, L. C. Carbone, J. E. Moser, F. Schreiber, J. Maier, J. V. Milic, M. Gratzel, The role of alkyl chain length and halide counter ion in layered Dion-Jacobson perovskites with aromatic spacers. *J. Phys. Chem. Lett.* **12**, 10325–10332 (2021).

76. M. Almalki, A. Ducinskas, L. C. Carbone, L. Pfeifer, L. Piveteau, W. Luo, E. Lim, P. A. Gaina, P. A. Schouwink, S. M. Zakeeruddin, J. V. Milic, M. Gratzel, Nanosegregation in arene-perfluoroarene pi-systems for hybrid layered Dion-Jacobson perovskites. *Nanoscale* **14**, 6771–6776 (2022).
